# N-Alkylation of functionalized amines with alcohols using a copper–gold mixed photocatalytic system

**DOI:** 10.1038/s41598-018-25293-z

**Published:** 2018-05-02

**Authors:** Lyu-Ming Wang, Yuna Morioka, Kellie Jenkinson, Andrew E. H. Wheatley, Susumu Saito, Hiroshi Naka

**Affiliations:** 1 Graduate School of Science and Research Center for Materials Science, Nagoya University, Chikusa, Nagoya 464-8602 Japan; 2 Department of Chemistry, University of Cambridge, Lensfield Road, Cambridge, CB2 1EW UK

## Abstract

Direct functionalization of amino groups in complex organic molecules is one of the most important key technologies in modern organic synthesis, especially in the synthesis of bio-active chemicals and pharmaceuticals. Whereas numerous chemical reactions of amines have been developed to date, a selective, practical method for functionalizing complex amines is still highly demanded. Here we report the first late-stage N-alkylation of pharmaceutically relevant amines with alcohols at ambient temperature. This reaction was achieved by devising a mixed heterogeneous photocatalyst *in situ* prepared from Cu/TiO_2_ and Au/TiO_2_. The mixed photocatalytic system enabled the rapid N-alkylation of pharmaceutically relevant molecules, the selective mono- and di-alkylation of primary amines, and the non-symmetrical dialkylation of primary amines to hetero-substituted tertiary amines.

## Introduction

**Figure 1:**
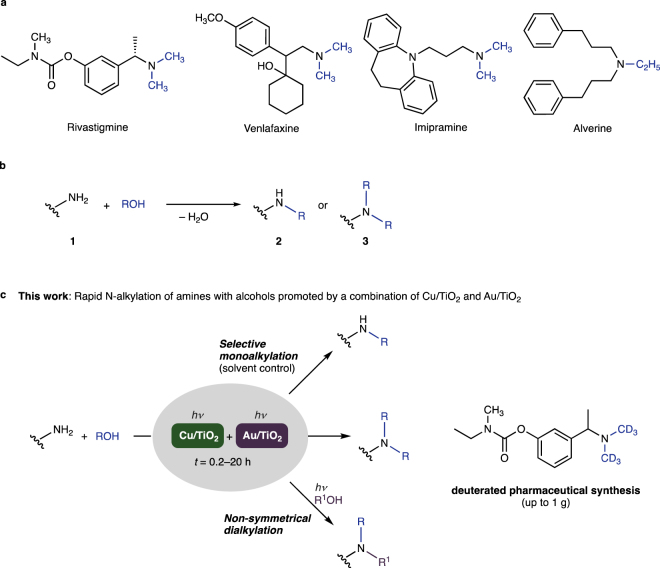
A mixture of two photocatalysts enables rapid N-alkylation of amines with alcohols under light irradiation. (**a**) Representative pharmaceuticals with N-alkylated groups: Rivastigmine (dementia), Venlafaxine (depression), Imipramine (depression), Alverine (gastropathy). (**b**) General scheme of N-alkylation of amines employing alcohols as alkylation reagents. (**c**) Outline of this study.

New synthetic strategies for producing complex organic molecules (*e.g*. bio-active natural products and pharmaceuticals) have been continuously demanded because only a limited number of chemical reactions have been available for selectively converting molecules bearing various functional groups into desired compounds^1^. Alkylamines represent an important class of functionality in valuable yet complex molecules^2^. Representative examples are shown in Fig. 1a. The alkylamino functionality is essential for drug design, as they often improve the oil-water partition coefficient (log *P*), reduce their toxicity, and increase their bioavailability (prodrugs)^3^.

The N-alkylation of primary amines (**1**) with alcohols (ROH) is a highly efficient method for preparing secondary alkylamines (**2**) and tertiary alkylamines (**3**, Fig. 1b)^4–10^. The most frequently used catalysts for this reaction include transition metal complexes (*e.g*. Ru^11–15^, Ir^16–20^, Fe^21–25^, Co^26,27^, and Mn^28,29^), and heterogeneous catalysts^30–35^. We have recently contributed to the development of molecular iron and phosphazene catalysts^21,36^. These methods allow green access to various amines without producing stoichiometric waste other than water. However, because they invariably require high reaction temperatures (typically >80 °C), their application to the late-stage functionalization^37^ of thermally unstable amines remains to be explored.

The photocatalytic N-alkylation of amines using alcohols is potentially a powerful method for functionalizing complex amines because this reaction generally proceeds at room temperature^38–43^. We have recently demonstrated that Ag/TiO_2_ promotes the chemoselective N-methylation of amines^44^. This photocatalyst enabled, for the first time, the gram-scale synthesis of tertiary amines by means of photocatalytic N-methylation at room temperature. However, the functionalization of complex amines by means of photocatalytic N-alkylation with alcohols remains unexplored simply because of the lack of a suitable method: the above-mentioned photocatalytic methods suffer from a limited scope in terms both of amine substrates and of alcohol reagents. Moreover, previously reported systems require the use of a large excess (>140 equiv) of alcohols and long reaction times (>4 h for completion of 0.2-mmol scale reactions). Thus, the benefits of developing an effective method for the room-temperature functionalization of complex amines are undeniable and warrant a thorough exploration of metal-loaded TiO_2_ systems. Herein we report the first late-stage N-alkylation of pharmaceutically relevant amines with alcohols at ambient temperature (Fig. 1c). The strategy of mixing two metal-loaded photocatalysts resulted in a synergistic increase in reaction rate for the N-alkylation of pharmaceutically relevant molecules. The selectivity with respect to the mono- and di-alkylation of primary amines was solvent-controlled. Facile synthesis of rivastigmine and alverine as well as venlafaxine-*d*_6_ and imipramine-*d*_3_ was demonstrated using methanol, ethanol, or deuterated methanol as alkylating reagent.

## Results

### Development of Cu–Au mixed photocatalytic system

**Figure 2:**
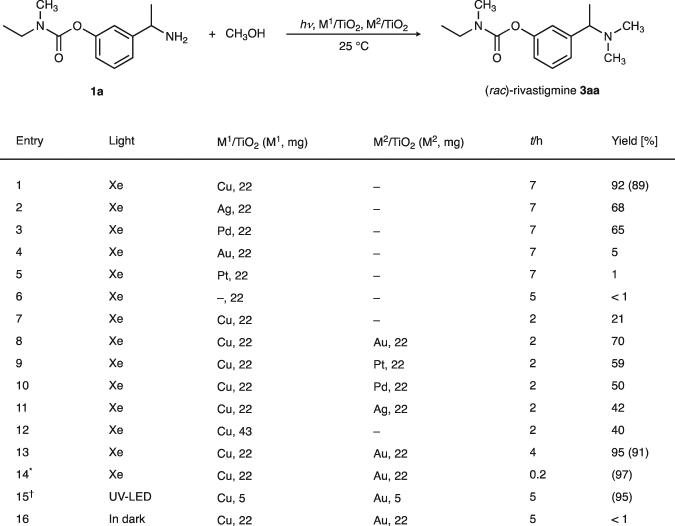
Light-induced dimethylation of **1a** with methanol leading to (*rac*)-rivastigmine (**3aa**). Typical conditions (entry 1): **1a** (1.0 mmol), Cu/TiO_2_ (22 mg), CH_3_OH (10 mL, 250 mmol, 250 equiv), 300 W Xe lamp with a UV-cold mirror (*λ* = 300–470 nm), Ar (1 atm), 25 °C. Metal content in 22 mg photocatalysts: Cu/TiO_2_ (5 wt% Cu, 16 µmol Cu), Au/TiO_2_ (5 wt% Au, 5.1 µmol Au), Pt/TiO_2_ (5 wt% Pt, 3.2 µmol Pt), Pd/TiO_2_ (5 wt% Pd, 9.5 µmol Pd), and Ag/TiO_2_ (4 wt% Ag, 8.5 µmol Ag) as determined by ICP-AES. Yields of **3aa** were determined by GC/MS or ^1^H NMR using 2,2-dimethylpropan-1-ol as an internal standard. Isolated yields are in parentheses. ^*^Conditions: **1a** (50 µmol), CH_3_OH (5 mL), 50 °C. ^†^Conditions: **1a** (0.2 mmol), CH_3_OH (2 mL), 32 W UV-LED lamp (*λ*_0_ = 365 nm).

We first examined the N,N-dimethylation of primary amine **1a** to give pharmaceutically relevant target (*rac*)-rivastigmine (**3aa**; a chiral (*S*)-form being used for Alzheimer’s and Parkinson’s diseases) using methanol with metal-loaded TiO_2_ photocatalysts under light irradiation (Xe lamp, *λ* = 300–470 nm) at 25 °C (Fig. 2). Among those tested, Cu/TiO_2_ was the most effective photocatalyst, giving the tertiary amine **3aa** in 89% isolated yield without involving the cleavage of the benzylic C–N bond or carbamate linkages (entry 1). While Ag/TiO_2_ and Pd/TiO_2_ also afforded **3aa**, neither Au/TiO_2_, Pt/TiO_2_ nor TiO_2_ were effective (entries 2–6). Conversions of **1a** in entries 1–5 were >96%, but quantitative analysis of intermediates failed due to their instability. Inspired by the recent advent of synergistic photocatalysis^45,46^ and bimetallic heterogeneous catalysis^31^, we next investigated a mixed photocatalytic system consisting of Cu/TiO_2_ and Au/TiO_2_ in order to further establish a more efficient reaction system. Au/TiO_2_ was selected because it represents the most reactive titania-based photocatalyst for the dehydrogenation of primary alcohols to aldehydes^47^. Indeed, mixing these two photocatalysts resulted in a synergistic acceleration of the dimethylation of **1a**, and gave **3aa** in 70% yield in 2 h (entry 8). This result proved to be better than those obtained using Cu in combination with other metals (entries 9–11) or the sole use of Cu (entry 12). The Cu–Au promoted dimethylation of **1a** (1.0 mmol) completed in just 4 h at 25 °C (entry 13). Furthermore, small-scale reaction proceeded more quickly at 50 °C to afford **3aa** in 97% yield within 12 min (entry 14). Such a rapid dimethylation of amines by methanol has hitherto been unachievable. Reaction could be similarly promoted by irradiation with a UV-LED (*λ*_0_ = 365 nm), and hardly proceeded in the dark (entries 15 and 16).

### Alkylation of amines by synergistic Cu–Au photocatalysis: substrate scope

**Figure 3:**
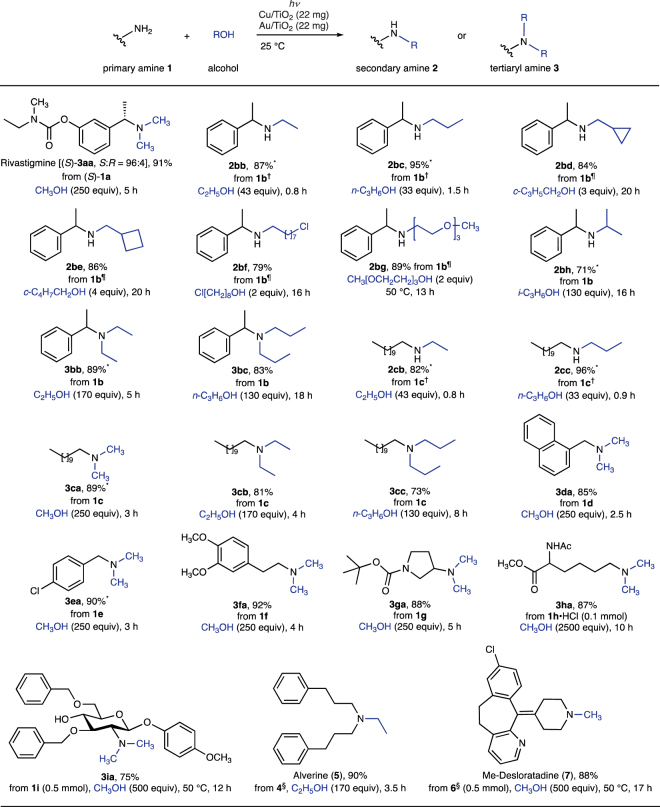
Substrate scope in synergistic Cu–Au photocatalysis. Conditions are analogous to Fig. 2, entry 13. Isolated yields are reported. ^*^Isolated yield of HCl salt. ^†^Conditions: **1** (0.2 mmol), hexane (10 mL). ^¶^Solvent = CPME (10 mL). ^§^**4** = bis(3-phenylpropyl)amine; **6** = desloratadine.

With this optimized photocatalytic system, next, the substrate scope was checked. The results of photocatalytic N-alkylation of amines are summarized in Fig. 3. A chiral substrate (*S*)-**1a** was straightforwardly converted to rivastigmine [(*S*)-**3aa**] with retention of the absolute configuration at the benzylic position. The selectivity for mono- and dialkylation of amines **1b** and **1c** could be precisely controlled by tuning the reaction conditions. Irradiation of amine **1b** and alcohols in hexane or cyclopentyl methyl ether (CPME) gave predominantly secondary amines **2bb**–**2bg**. For the first time, photocatalytic methods have been successfully used with the presence of cyclopropyl, cyclobutyl, chloroalkyl, and oligomeric alkoxy groups in **2bd**–**2bg** being tolerated. Moreover, the use of only 2–4 equiv of alcohol was sufficient for N-alkylation of **1b** to **2bd**–**2bg**. Exclusive monoalkylation of **1b** with 2-propanol to **2bh** proceeded under neat conditions. Selectivity for monoalkylation to **2bb**–**2bh** over dialkylation was >97:3, as determined by GC/MS analysis. In contrast, dialkylation of **1b** with ethanol and 1-propanol under neat conditions with longer irradiation time gave tertiary amines **3bb** and **3bc** in 89% and 83% yields, respectively. Similar results were also seen for amine **1c**. Amines bearing core structures important to pharmaceuticals were also efficiently converted to the desired products **3da**–**3ga**. In all these cases, the superior reactivity of the Cu–Au system with respect to either Cu/TiO_2_ or Au/TiO_2_ was confirmed (Table S6 in the Supplementary Information). Lysine derivative **1 h**•HCl and protected glucosamine **1i** were converted to tertiary amines **3 ha** and **3ia** in good yields, respectively. This method was also effective in the synthesis of alverine (**5**, a drug used for irritable bowel syndrome) and the functionalization of desloratadine (**6**, a drug used for treating allergies).

### Sequential, non-symmetrical dialkylation

**Figure 4:**
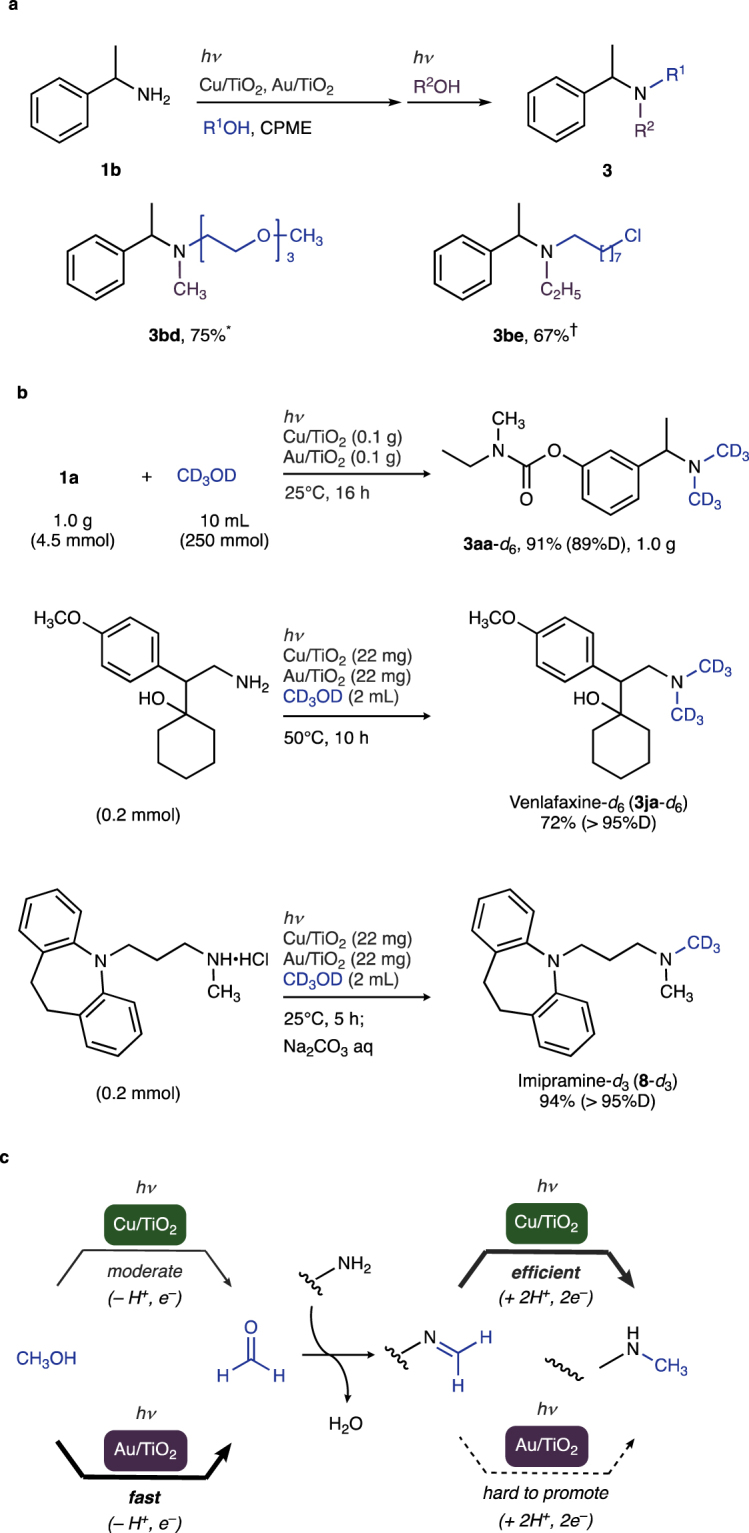
N-Alkylation of Amines by the Cu–Au mixed photocatalytic system. (**a**) Non-symmetrical N,N-dialkylation. Reaction conditions: ^*^1b (1.0 mmol), Cu/TiO_2_ (22 mg), Au/TiO_2_ (22 mg), triethylene glycol monomethyl ether (2 equiv), CPME (10 mL), *hv*, Ar, 50 °C, 13 h; CH_3_OH (5 mL), 50 °C, 6 h. ^†^**1b** (1.0 mmol), Cu/TiO_2_ (22 mg), Au/TiO_2_ (22 mg), 8-chloro-1-octanol (2 equiv), CPME (10 mL), *hv*, Ar, 25 °C, 16 h; C_2_H_5_OH (5 mL), 25 °C, 16 h. (**b**) Synthesis of deuterated drugs. (**c**) Synergistic effect in the Cu–Au mixed photocatalytic system.

Having demonstrated that the photocatalytic system enabled the precise control of mono- vs di-alkylation, one-pot, sequential synthesis of non-symmetrical tertiary amines from primary amines and alcohols was demonstrated (Fig. 4a). Successive reaction of **1b** with alcohols R^1^OH and R^2^OH yielded non-symmetrical amines **3bd** (75%) and **3be** (67%).

### Application to deuterated drug synthesis

Regio-specifically deuterated drugs have recently begun receiving significant attention because of their improved metabolic stability with respect to their hydrogen analogues. This is making the development of new and efficient synthetic methods for their production an important emergent area in medicinal chemistry^48^. Here, deuterium atoms were precisely installed to pharmaceutical structures at the desired methyl groups by using Cu–Au mixed photocatalysis (Fig. 4b). Photocatalytic reaction of **1a** with commercially available deuterated methanol (CD_3_OD) produced hexadeuterated (*rac*)-rivastigmine (**3aa**-*d*_6_) efficiently on a gram scale (1.0 g, 91% yield). This protocol also allowed us to rapidly access other hexa- and trideuterated drugs such as venlafaxine-*d*_6_ (**3ja**-*d*_6_) and imipramine-*d*_3_ (**8**-*d*_3_).

### Mechanistic discussion

The origin of the superior reactivity of the mixed Cu–Au photocatalytic system is under investigation. Whereas photocatalytic activity of Au/TiO_2_ in methanol dehydrogenation is higher than that of Cu/TiO_2_ (Figure S1 in the Supplementary Information), reducing the reactivity of Au/TiO_2_ in producing **3aa** is significantly lower than that of Cu/TiO_2_ (Fig. 2, entry 4 vs entry 1). These results imply a greater contribution by Au/TiO_2_ to alcohol dehydrogenation and by Cu/TiO_2_ to imine reduction, respectively (Fig. 4c).

When the photocatalyst recyclability was tested, the reused mixed Cu–Au photocatalysts were found to have comparable reactivity to the pristine mixture for 10 cycles (82–98% yield of **3aa**, Table S7 in the Supplementary Information). The fresh and used photocatalysts were investigated by (scanning) transmission electron microscopy [(S)TEM] and powder X-ray diffraction (Figures S2–S20). TEM indicated that Cu/TiO_2_ comprised Cu nanoparticles of mean particle size 1.7 ± 0.3 nm, while Au/TiO_2_ comprised Au nanoparticles of mean particle size 7.75 ± 0.46 nm (Figures S2–S6). Whereas bright field imaging of the combined photocatalysts suggests the presence of both Cu and Au nanoparticles (Figure S7), STEM analysis suggests a more complicated picture (Figures S8–S19). Hence, a pristine sample of combined Cu/TiO_2_ and Au/TiO_2_ photocatalysts reveals an essentially uniform Cu background punctuated by discrete Au nanoparticles (Figure S19a). In contrast, after photocatalytic reaction (Table S7, run 1) the Cu signals have become more localized and coincident with the Au signals (Figure S19b). This is consistent with the formation of individually heterobimetallic nanoparticles at room temperature by light irradiation, in spite of the fact that high temperatures (*ca*. 160 °C) are normally needed for their formation^49^. The formation of similar Cu–Au heterobimetallic nanoparticles was seen after the irradiation of a mixture of Cu/TiO_2_ and Au/TiO_2_ in methanol in the absence of amine (Figures S20 and S21). This pre-irradiated Cu–Au photocatalyst also showed similar reactivity to the pristine analogue in the N,N-dimethylation of **1a** (Table S8, entry 2). Nevertheless, the pristine mixture of Cu/TiO_2_ and Au/TiO_2_ showed slightly higher reactivity than the pre-irradiated mixed photocatalysts (Table S8, entry 1 vs entries 2 and 3), implying that the formation of heterobimetallic nanoparticles is not prerequisite for the high reactivity of the current photocatalytic system.

## Conclusion

We have established a mixed Cu–Au photocatalytic system for the rapid N-alkylation of pharmaceutically relevant amines. The synthesis and functionalization of drugs, the controllable mono- and dialkylation of primary amines, and the non-symmetrical dialkylation of primary amines to hetero-substituted tertiary amines have been demonstrated by the mixed photocatalytic system. Studies for further improvement of the photocatalytic system, targeting the ultra-fast N-methylation of amines applicable to ^11^C-positron emission tomography (PET) using ^11^CH_3_OH are currently underway^50^.

## Methods

A representative procedure for N-methylation of **1a** to **3aa** by Cu (5 wt%)/TiO_2_ and Au (5 wt%)/TiO_2_ (Fig. 2, entry 13) is as follows: Cu (5 wt%)/TiO_2_ (22 mg, 1.6 mol% Cu), Au (5 wt%)/TiO_2_ (22 mg, 0.51 mol% Au), anhydrous CH_3_OH (10 mL, 250 mmol), and **1a** (222.1 mg, 1.00 mmol) were added successively to a cylindrical Pyrex glass reaction vessel (diameter: 50 mm, height: 130 mm with a top window made of Pyrex) connected to a rubber balloon. After the resulting mixture was sonicated for 30 sec and deaerated by Ar bubbling *via* cannula for 5 min, the vessel was immersed in a water bath (kept at 25 °C using a cooling circulator), and stirred for 4 h with irradiation [300 W Xe lamp (Ushio: BA-x300/ES1 Technology; CERMAX PE300BF) equipped with a UV cold mirror (*λ* = 300–470 nm)]. The presence of **3aa** (95% yield) in the reaction mixture was indicated by GC/MS analysis using 2,2-dimethylpropan-1-ol as an internal standard. The reaction mixture was filtered through a 0.45 µm membrane filter and the photocatalyst was washed with CH_3_OH (10 mL). HCl (35–37%, 12 M aq, 0.5 mL, 6 mmol) was added to the solution (pH 1–2) and stirred at rt for 30 min. After methanol was evaporated, the residue was dissolved in H_2_O (20 mL) and washed with ethyl acetate (20 mL). To the aqueous layer, sodium carbonate (s) was added (pH 10) and extracted with ethyl acetate (2 × 30 mL). After washing with brine, the organic layer was dried over Na_2_SO_4_ and concentrated under reduced pressure to afford (*rac*)-rivastigmine (**3aa**) as a light-yellow oil (227.8 mg, 91% yield). All new compounds were fully characterized (see Supplementary Information).

## Electronic supplementary material

Supplementary information
