# Author Correction: N-Alkylation of functionalized amines with alcohols using a copper–gold mixed photocatalytic system

**DOI:** 10.1038/s41598-018-27270-y

**Published:** 2018-06-19

**Authors:** Lyu-Ming Wang, Yuna Morioka, Kellie Jenkinson, Andrew E. H. Wheatley, Susumu Saito, Hiroshi Naka

**Affiliations:** 1 Graduate School of Science and Research Center for Materials Science, Nagoya University, Chikusa, Nagoya, 464-8602 Japan; 2 Department of Chemistry, University of Cambridge, Lensfield Road, Cambridge, CB2 1EW UK

Correction to: *Scientific Reports* 10.1038/s41598-018-25293-z, published online 02 May 2018

In Figure 4c, there is an error in the wavy line of the product structure as it was wrongly disconnected from the amine group. The correct Figure 4 appears below as Figure 1.

**Figure 1:**
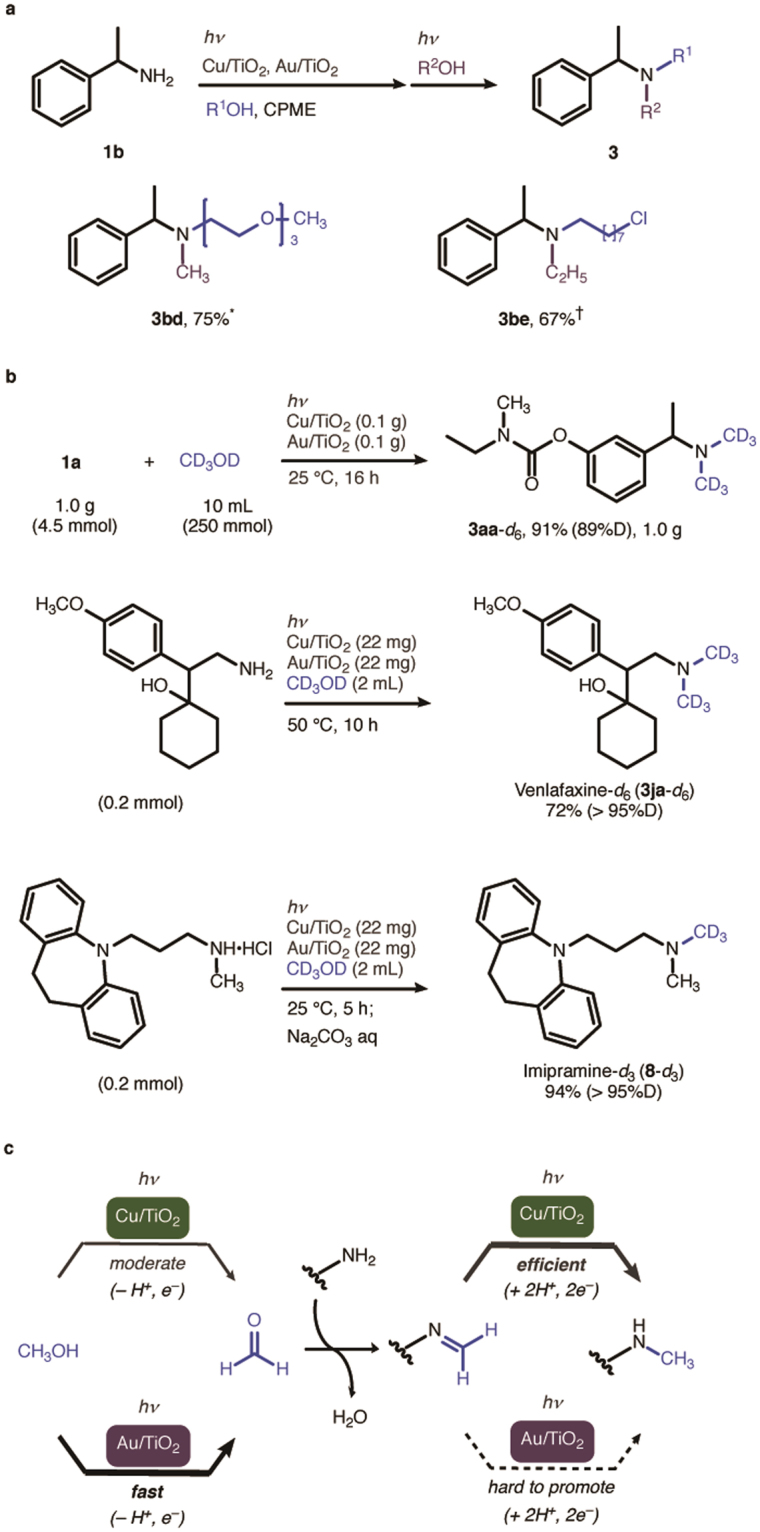
N-Alkylation of Amines by the Cu–Au mixed photocatalytic system. (**a**) Non-symmetrical N,N-dialkylation. Reaction conditions: *1b (1.0 mmol), Cu/TiO_2_ (22 mg), Au/TiO_2_ (22 mg), triethylene glycol monomethyl ether (2 equiv), CPME (10 mL), *hv*, Ar, 50 °C, 13 h; CH3OH (5 mL), 50 °C, 6 h. ^†^**1b** (1.0 mmol), Cu/TiO_2_ (22 mg), Au/TiO_2_ (22 mg), 8-chloro-1-octanol (2 equiv), CPME (10 mL), *hv*, Ar, 25 °C, 16 h; C2H5OH (5 mL), 25 °C, 16 h. (**b**) Synthesis of deuterated drugs. (**c**) Synergistic effect in the Cu–Au mixed photocatalytic system.

