# Supplementary material for: N-Alkylation of functionalized amines with alcohols using a copper–gold mixed photocatalytic system
Source: Sci Rep. 2018 May 2;8:6931. doi: 10.1038/s41598-018-25293-z (PMC5931965; doi:10.1038/s41598-018-25293-z)
Supplement: Supplementary file 1 — Supplementary information [file 41598_2018_25293_MOESM1_ESM.pdf]

Supporting Information

N-Alkylation of Functionalized Amines with Alcohols Using a Copper–Gold Mixed Photocatalytic System

Lyu-Ming Wang, Yuna Morioka, Kellie Jenkinson, Andrew E. H. Wheatley,*
Susumu Saito* and Hiroshi Naka*

Table of Contents

1.	General Comments	S3
2.	Materials	S4
3.	Substrate Synthesis	S5
4.	Preparation of Photocatalysts (Table S1, Figure S1)	S7
5.	Characterization of Photocatalysts (Figures S2–S22)	S10
6.	Photocatalytic N-Methylation of **1a** to (*rac*)-Rivastigmine (**3aa**) (Tables S2–S5)	S25
7.	Photocatalytic N-Alkylation of Amines (Table S6)	S30
8.	Photocatalyst Recycling Experiments (Tables S7 and S8)	S43
9.	NMR Charts	S45

1. General Comments

Reactions were conducted using a 300 W Xe lamp (Ushio: BA-x300/ES1 Technology; CERMAX PE300BF) equipped with a UV cold mirror (λ = 300–470 nm) or using 32 W UV-LED lamps [two 16W UV-LED lamps (λ_0 = 365 nm), LN-128UV2-365 (CCS Inc.)]. GC/MS analyses were performed on Agilent 6850 series network GC system and Agilent 5975C series Mass Selective Detector (EI) [column: HP-5MS capillary column (l = 30 m, d = 0.25 mm, film thickness = 0.25 µm); chromatographic elution: isothermal at 50 ºC for 1 min, 50–250 ºC at a rate of 20 ºC/min, isothermal at 250 ºC for 4 min, 250–300 ºC at a rate of 20 ºC/min, isothermal at 300 ºC for 1 min; carrier gas: He (1.5 mL/min, at 27 psi)]. The amount of H_2 was determined by Micro-GC (Agilent 490) using argon as a carrier. ^{1}H and ^{13}C NMR spectra were recorded on a JEOL ECA-500 (500 MHz for ^{1}H, 125 MHz for ^{13}C) or JEOL ECA-600 (600 MHz for ^{1}H, 150 MHz for ^{13}C) at 27 °C. Chemical shifts are reported as δ in ppm and are internally referenced to tetramethylsilane (TMS, 0.0 ppm for ^{1}H) and $CDCl_3$ (77.2 ppm for ^{13}C). The following abbreviations are used: s = singlet, d = doublet, t = triplet, q = quartet, quint = quintet, bs = broad singlet, dd = doublet of doublet, dq = doublet of quartet, and m = multiplet. High-resolution mass spectra (HRMS) were obtained from micrOTOF-QII (ESI, Bruker). Inductively coupled plasma atomic emission spectrophotometry (ICP-AES) was carried out with Vista Pro (Agilent) using yttrium as an internal standard after digestion of samples (10 mg) with aqua regia (2 mL) for 12 h. Optical rotation values were recorded on Polarimeter P-1010-GT (JASCO). High performance liquid chromatography (HPLC) spectra were recorded on Prominence 2000 (SHIMADZU). Transmission electron microscope (TEM) measurements used a FEI Philips Tecnai 20 TEM. Typical bright-field (BF) imaging was achieved with a 70 µm objective aperture, CCD camera and 200 KeV source. Scanning TEM (STEM) high-angle annular dark-field (STEM HAADF) imaging and energy dispersive spectroscopy (EDS) analysis was achieved with 70 µm C1 and C2 condenser apertures. For powder X-ray diffraction (PXRD), the already fine powder was placed on a zero-background sample holder. PXRD measurements were achieved using Ni-filtered CuKα radiation from a PAN-analytical X-ray generator operated at 40 kV and 40 mA. The X-ray generator was coupled with a PW3071/60 Bracket goniometer for sample mounting. Step-scan data (step size 0.02° 2θ, counting time 3 s/step) were recorded. Acquisition times were 3 hours per sample. NMR, HRMS, optical rotation, and ICP-AES analyses were carried out at the Chemical Instrumental Center, Research Center for Materials Science, Nagoya University. (S)TEM, EDS, and PXRD analyses were carried out at Department of Chemistry, University of Cambridge.

2. Materials

Substrates: **1a** and **4** were prepared as shown in the next section. (*S*)-**1a**[1] and (*S*)-**3aa**[2] for HPLC analyses were prepared according to literature procedures. 1-[2-Amino-1-(4-methoxyphenyl)ethyl]cyclohexanol was prepared by treatment of its hydrochloride salt (from Tokyo Chemical Industry) with sodium carbonate aq and exaction with ethyl acetate. Following substrates were purchased from commercial suppliers: **1b**, **1d**, **1 h**, and **6** from Sigma-Aldrich; **1c**, **1e**, and 10,11-dihydro-*N*-methyl-5*H*-dibenz[*b*,*f*]azepine-5-propanamine hydrochloride from Wako Chemicals; **1f**, **1g**, and **1i** from Tokyo Chemical Industry.

Alcohols: Alcohols were purchased from commercial suppliers: methanol (dehydrated), and methanol-d_4 (99.8%D) from Kanto Chemicals; ethanol (dehydrated), and 2-propanol from Wako Chemicals; 1-propanol (dehydrated) from Sigma-Aldrich; cyclopropanemethanol, cyclobutanemethanol, 8-chloro-1-octanol, and 2-[2-(2-methoxyethoxy)ethoxy]ethanol from Tokyo Chemical Industry.

Photocatalyst Precursors: Titanium(IV) oxide (Aeroxide® P25) and copper(II) nitrate hemi(pentahydrate) were purchased from Sigma-Aldrich. Hydrogen tetrachloroaurate(III) tetrahydrate and hydrogen hexachloroplatinate(IV) hexahydrate were purchased from Kanto Chemicals. Silver(I) nitrate and palladium(II) acetate were purchased from Wako Chemicals.

Others: Hydrogen chloride in diethyl ether (2 M), sodium triacetoxyborohyride, and titanium(IV) ethoxide were purchased from Sigma-Aldrich. Ammonium formate, chloro[4-(dimethylamino)-*N*-(dimethylamino)phenyl-2-pyridine-carboxamidato]-(pentamethyl-cyclopentadienyl) iridium(III) [Ir-complex], diethyl ether (dehydrated), diethylamine, *N,N*-diethylmethylamine, hydrochloric acid, sodium carbonate, sodium sulfate, potassium carbonate, and triethylamine were purchased from Kanto Chemicals. (*S*)-(–)-*tert*-Butylsulfinamide, 2,2-dimethylpropan-1-ol, *N*-ethyl-*N*-methylcarbamoyl chloride, 3´-hydroxyacetophenone, 3-phenylpropyl bromide, 3-phenylpropylamine, sodium borohydride, and trifluoroacetic acid were purchased from Tokyo Chemical Industry. Acetic acid, formaldehyde solution (37% in H_2O), nitric acid, sodium hydroxide, sodium acetate, and standard solutions for ICP-AES [$Y(NO_3)_3$ in HNO_3 aq, 1.00 mg Y per mL, 1000 ppm; $Cu(NO_3)_2$ in HNO_3 aq, 1.00 mg Cu per mL, 1000 ppm; $HAuCl_4$ in HCl aq, 1.00 mg Au per mL, 1000 ppm] were purchased from Wako Chemicals. Water was deionized before use by Demiace DX-07 (KURITA Water Industries LTD.). Other solvents were purchased from Kanto Chemicals.

Silica gel (diameter: 60–210 µm) was purchased from Kanto Chemicals. 50 NH_2 silica gel (diameter: 38–63 µm) was purchased from Wako Chemicals.

3. Substrate Synthesis

3-Acetylphenyl ethyl(methyl) carbamate (S1a)

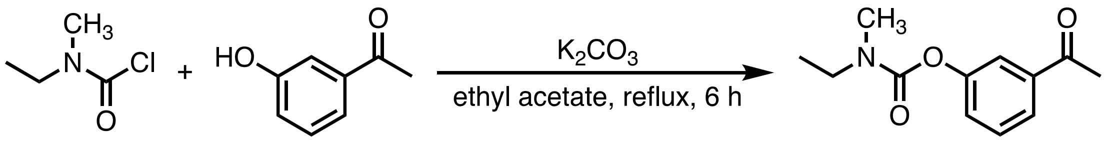

S1a was prepared by slightly modifying a literature procedure.[1] To a 300-mL round-bottom flask equipped with a stirring bar, 3´-hydroxyacetophenone (6.8 g, 50 mmol), *N*-ethyl-*N*-methylcarbamoyl chloride (6.7 g, 55 mmol), K_2CO_3 (20 g, 150 mmol), and ethyl acetate (100 mL) were added. The reaction mixture was refluxed for 6 h (completion of reaction was monitored by TLC). After the reaction mixture was cooled to 25 °C, K_2CO_3 was filtered and organic layer was washed with H_2O (3 × 50 mL). Then the organic layer was dried over Na_2SO_4 and concentrated under reduced pressure to afford **S1a** as a light-yellow oil (10.6 g, 95%). Analytical data for **S1a** (observed as a 1:1 *cis*/*trans* isomeric mixture around the carbamate moiety): ^{1}H NMR (500 MHz, $CDCl_3$) δ 1.19 (t, *J* = 7.2 Hz, 1.5H), 1.25 (t, *J* = 7.2 Hz, 1.5H), 2.58 (s, 3H), 3.00 (s, 1.5H), 3.07 (s, 1.5H), 3.41 (q, *J* = 7.1 Hz, 1H), 3.48 (q, *J* = 7.1 Hz, 1H), 7.30–7.36 (m, 1H), 7.44 (dd, *J* = 7.5, 8.0 Hz, 1H), 7.70 (s, 1H), 7.77 (d, *J* = 7.4 Hz, 1H); ^{13}C{^{1}H} NMR (125 MHz, $CDCl_3$) δ 12.5 (0.5C), 13.3 (0.5C), 26.7, 33.9 (0.5C), 34.3 (0.5C), 44.1 (0.5C), 44.2 (0.5C), 121.7, 125.1, 126.8, 129.5, 138.4, 151.8, 154.2, 197.2; HRMS (ESI) calcd for $C_{12}H_{15}NO_3Na^+$ ([M + Na]$^+$) 244.0944, found 244.0953. These data are consistent with the literature values.[1]

3-(1-Aminoethyl)phenyl ethyl(methyl)carbamate (1a)

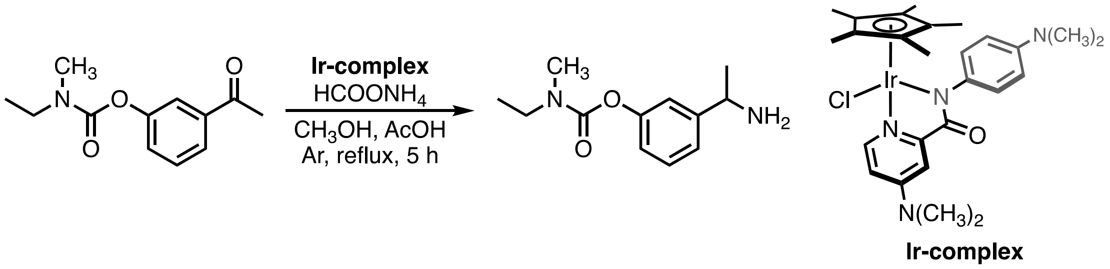

To a 100-mL Schlenk tube equipped with a stirring bar, 3-acetylphenyl ethyl(methyl) carbamates (**S1a,** 2.0 g, 9.0 mmol), ammonium formate (2.85 g, 45.2 mmol), chloro[4-(dimethylamino)-*N*-(dimethylamino)phenyl-2-pyridine-carboxamidato]-(pentamethylcyclopentadienyl)iridium(III) (Ir-complex, 5.8 mg, 9.0 µmol)[3], acetic acid (1.09 g, 18.1 mmol), and methanol (12 mL) were added. The solution was degassed by three freeze-thaw cycles and refluxed for 5 h. After the reaction mixture was cooled to 25 °C, ammonium formate was precipitated upon addition of ethyl acetate (50 mL).

After ammonium formate was filtered, HCl (35–37%, 12 M aq) was added to the solution (pH 1–2). The mixture was stirred at 25 °C for 30 min and was extracted with H_2O (50 mL). The aqueous layer was adjusted to pH 10 using sodium carbonate (s) and was extracted with CH_2Cl_2 (2 × 100 mL). After washed with brine, the organic layer was dried over Na_2SO_4 and concentrated under reduced pressure. The residue was purified by silica gel column chromatography (ethyl acetate) to afford **1a** (1.46 g, 73%). Analytical data for **1a** (observed as a 1:1 *cis*/*trans* isomeric mixture around the carbamate moiety): ^{1}H NMR (500 MHz, $CDCl_3$) δ 1.19 (t, J = 6.9 Hz, 1.5H), 1.24 (t, J = 6.9 Hz, 1.5H), 1.37 (d, J = 6.3 Hz, 3H), 1.52 (s, 2H), 2.98 (s, 1.5H), 3.06 (s, 1.5H), 3.40 (q, J = 7.1, 1H), 3.46 (q, J = 7.1, 1H), 4.10 (q, J = 6.7 Hz, 1H), 6.98 (d, J = 7.4 Hz, 1H), 7.10–7.19 (m, 2H), 7.30 (dd, J = 7.4, 8.0 Hz, 1H); ^{13}C{^{1}H} NMR (125 MHz, $CDCl_3$) δ 12.3 (0.5C), 13.1 (0.5C), 25.5, 33.7 (0.5C), 34.1 (0.5C), 43.9, 50.9, 118.9, 120.0, 122.4, 129.1, 149.3, 151.6, 154.3, 154.5; HRMS (ESI) calcd for $C_{12}H_{18}N_2O_2Na^+$ ([M + Na]$^+$) 245.1260, found 245.1252. These data are broadly in line with the literature values for *S*-**1a**.[1]

***N*-(3-Phenylpropyl)benzenepropanamine (4)**

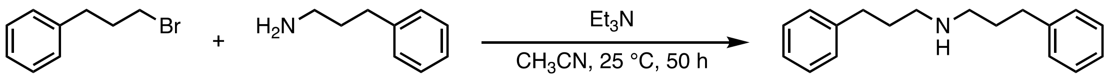

To a 50-mL round-bottom flask equipped with a stirring bar, 3-phenylpropylamine (1.1 g, 8 mmol), acetonitrile (10 mL), triethylamine (0.8 g, 8 mmol), and 3-phenylpropyl bromide (0.8 g, 4 mmol) were added. The reaction mixture was stirred at 25 °C for 50 h (completion of reaction was monitored by TLC). After the solution was concentrated under reduced pressure, triethylamine HBr salt was precipitated upon addition of ethyl acetate (50 mL), after triethylamine HBr salt was filtered, the residue was concentrated under reduced pressure and purified by silica gel column chromatography (hexane/ethyl acetate 2:1) to afford **4** as a light-yellow oil (0.4 g, 40%). Analytical data for **4**: ^{1}H NMR (500 MHz, $CDCl_3$) δ 1.01 (bs, 1H), 1.80 (quint, J = 7.4 Hz, 4H), 2.63 (q, J = 7.8 Hz, 8H), 7.16–7.18 (m, 6H), 7.27 (t, J = 7.4 Hz, 4H); ^{13}C{^{1}H} NMR (125 MHz, $CDCl_3$) δ 31.9 (2C), 33.8 (2C), 49.7 (2C), 125.9 (2C), 128.4 (4C), 128.5 (4C), 142.3 (2C); HRMS (ESI) calcd for $C_{18}H_{23}NH^+$ ([M + H]$^+$) 254.1903, found 254.1890. These data are consistent with the literature values.[4]

4. Preparation of Photocatalysts

A typical procedure for the preparation of Cu (5 wt %)/TiO_2

In a 300-mL round-bottom flask wrapped in aluminum foil, $Cu(NO_3)_2$•$2.5H_2O$ (0.482 g, 2.07 mmol) was dissolved in deionized H_2O (100 mL), then TiO_2 (2.5 g) was added to the solution. After the resulting suspension was rotated for 30 min at 50 °C on a rotary evaporator, the solvent was removed under vacuum (20 mm Hg), then the resulted light blue powder was transferred into a 500-mL round-bottom flask wrapped in aluminum foil. After the flask was successively evacuated and refilled with N_2, the solid was suspended in deoxygenated, deionized H_2O (76 mL). The formed slurry was stirred for 20 min, treated with CH_3CO_2Na (1.0 M aq, 120 mL, 120 mmol, 58 equiv), and stirred for additional 15 min. To the suspension, a mixture of $NaBH_4$ (14.0 mmol, 7 equiv) and degassed H_2O (126 mL) was added dropwise, and the resulted suspension was stirred at 25 °C for 3.5 h. The stirring was stopped to settle down the solid particles and the supernate was roughly removed *via* cannula. The remaining suspension was transferred to a 50-mL Falcon tube and centrifuged (3500 rpm, 10 min). After the supernate was removed by decantation, the solid residue was suspended in deionized H_2O (20 mL) and stirred overnight under N_2. Centrifugation of the mixture (3500 rpm, 10 min) and removal of the supernate by decantation yielded Cu (5 wt %)/TiO_2 as a dark-cyan solid after drying in vacuum (0.01 mm Hg) at 25 °C for 12 h (2.26 g, 86% yield). The copper content was 4.57 ± 0.026 wt % [determined by ICP-AES, after digestion of Cu (5 wt %)/TiO_2 (10.1 mg) using aqua regia (2 mL) for 12 h]. Other photocatalysts were analogously prepared by following the same procedure using the amounts of reagents listed in Table **S1**. Methods and data for Ag (4 wt %)/TiO_2, Pt (5 wt %)/TiO_2, and Pd (5 wt %)/TiO_2, are shown elsewhere.[5,6]

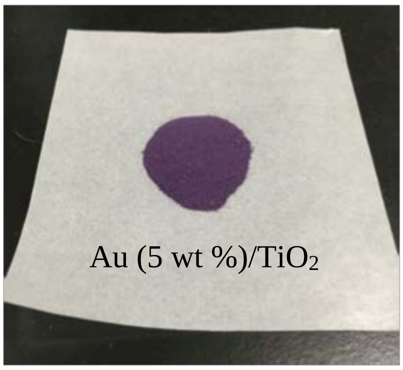

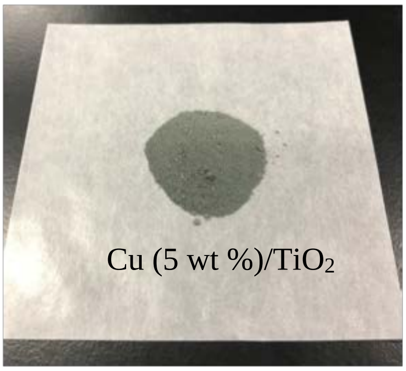

Table S1. Preparation of metal-loaded photocatalysts

	reagents used (mg)			
photocatalyst	$Cu(NO_3)_2•2.5H_2O$	$HAuCl_4•4H_2O$	TiO_2	metal content (wt %)
Cu (1 wt %)/TiO_2	365	–	990	Cu 0.89 ± 0.003
Cu (5 wt %)/TiO_2	482	–	2489	Cu 4.57 ± 0.026
Cu (11 wt %)/TiO_2	366	–	900	Cu 10.58 ± 0.084
Au (0.6 wt %)/TiO_2	–	5.2	500	Au 0.56 ± 0.002
Au (1 wt %)/TiO_2	–	10.9	515	Au 1.08 ± 0.010
Au (2 wt %)/TiO_2	–	26.5	494	Au 2.35 ± 0.014
Au (5 wt %)/TiO_2	–	265	2405	Au 4.61 ± 0.036
Au (6 wt %)/TiO_2	–	78.4	463	Au 6.4 ± 0.024

H_2 evolution comparison

Photocatalyst [Au (5 wt %)/TiO_2 (22 mg, 0.5 mol % Au) or Cu (5 wt %)/TiO_2 (22 mg, 1.6 mol % Cu)] and anhydrous CH_3OH (10 mL, 250 mmol) were added successively to a 3-necked cylindrical Pyrex glass reaction vessel (diameter: 50 mm, height: 130 mm with a top window made of Pyrex). After the resulting mixture was sonicated for 30 sec and deaerated by Ar bubbling *via* cannula for 5 min, the vessel was closed and immersed in a water bath (kept at 25 ºC using a cooling circulator), and stirred for 1 h with irradiation [300 W Xe lamp (Ushio: BA-x300/ES1 Technology; CERMAX PE300BF) equipped with a UV cold mirror (λ = 300–470 nm)]. A larger amount of hydrogen gas was generated with Au/TiO_2 than with Cu/TiO_2, as indicated by semi-quantitative analysis of gas phase in the reaction vessel using micro-GC (see Figure S1); the peak area of H_2 using Au/TiO_2 is approximately twice as large as that obtained using Cu/TiO_2 (S_{Au}/S_{Cu} = 2.1).

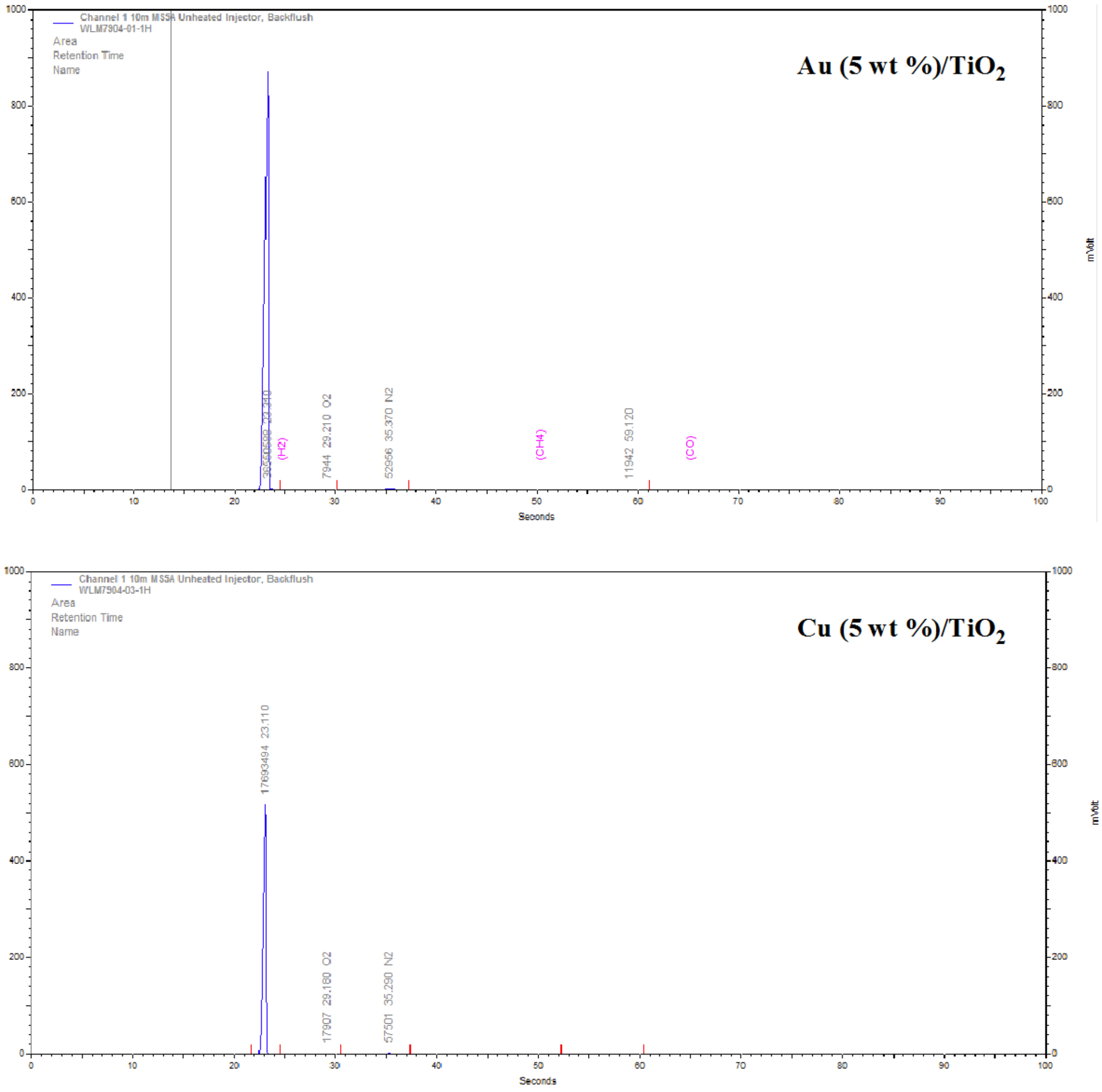

Figure S1. Micro-GC chromatograms of the gas phase after light irradiation of methanol (1 h, 25 °C, Ar) in the presence of Au (5 wt %)/TiO_2 and Cu (5 wt %)/TiO_2. 2 mL of the gas phase was subjected to analysis using a syringe.

5. Characterization of Photocatalysts

5.1. Transmission electron microscopy

Dry powdered samples were prepared for analysis by lightly crushing through folding in weighing paper, with any larger particles crushed using a pestle for 5 seconds. The electron microscopy grid was submerged into the power briefly before analysis. For unmixed photocatalyst systems, lacey carbon copper TEM grids were used. In the case of mixed photocatalyst systems, 30 nm Si_3N_4 grids were used.

Mixed photocatalysts were investigated both pre- and post-catalytic testing. The following synthetic procedure was used for the relevant catalytic test: 1-undecanamine (**1c**, 1.0 mmol), methanol (10 mL), pre-mixed photocatalyst [Au (5 wt %)/TiO_2 (22 mg) and Cu (5 wt %)/TiO_2 (22 mg)] were added successively to a cylindrical Pyrex glass reaction vessel (diameter: 50 mm, height: 130 mm with a Pyrex top window) connected to a balloon. After sonication (30 sec) and deaeration (Ar bubbling via cannula for 5 min), the vessel was immersed in a water bath (kept at 25 ºC using a cooling circulator), and stirred for 1/3/20 h with irradiation [300 W Xe lamp (Ushio: BA-x300/ES1 Technology; CERMAX PE300BF) equipped with a UV cold mirror (λ = 300–470 nm)]. The reaction mixture was transferred to a 50-mL Falcon tube and centrifuged (3500 rpm, 10 min). The supernate was obtained by decantation. The photocatalyst was washed (methanol, 10 mL) and centrifuged again. After the methanol was decanted, the photocatalyst was dried *in vacuo* (0.01 mm Hg) at 25 °C overnight.

Cu (5 wt %)/TiO_2

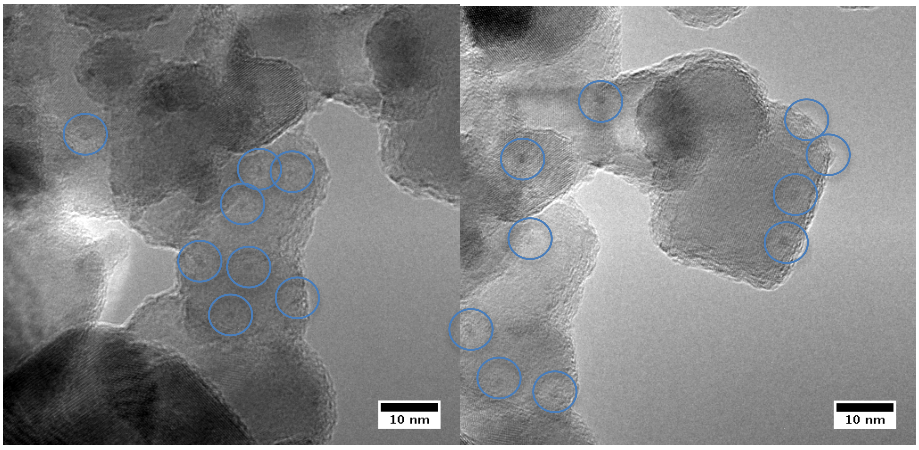

Figure S2. Representative BFTEM images of Cu (5 wt %)/TiO_2 on a lacey carbon Cu grid. (Scale bars: 10 nm; ○ examples of Cu NPs).

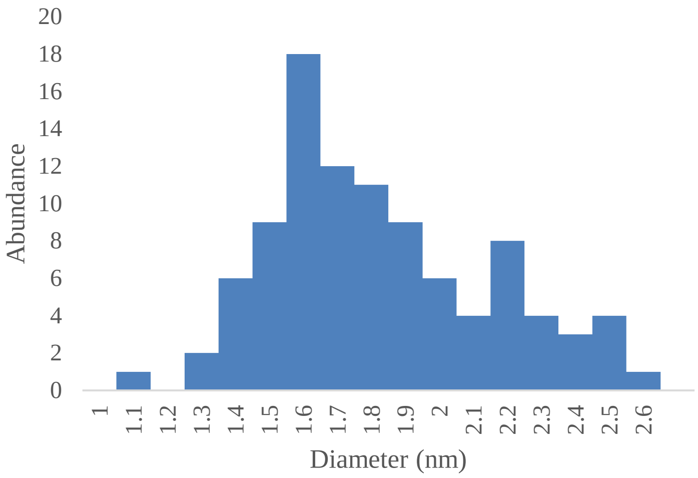

Figure S3. Particle size distribution for the Cu NPs in Cu (5 wt %)/TiO_2. Mean particle size = 1.74 ± 0.35 nm. SD = 0.27. *N* = 100.

Au (5 wt %)/TiO_2

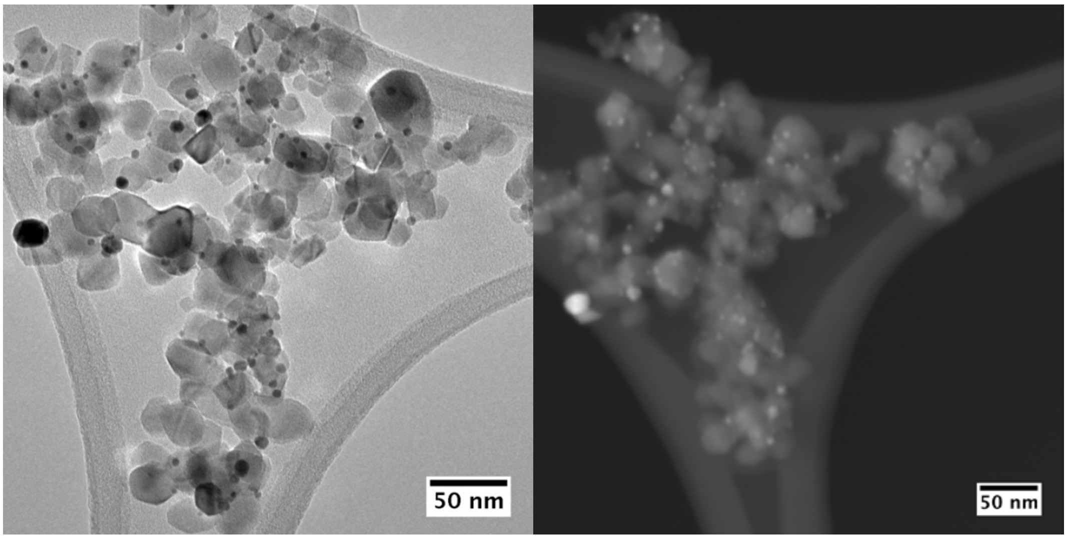

Figure S4. Left: Representative BFTEM imaging of Au (5 wt %)/TiO_2 on a lacey carbon Cu grid. Right: Identical image by STEM-HAADF imaging showing brighter Au NPs on the darker TiO_2 support. (Scale bars: 50 nm).

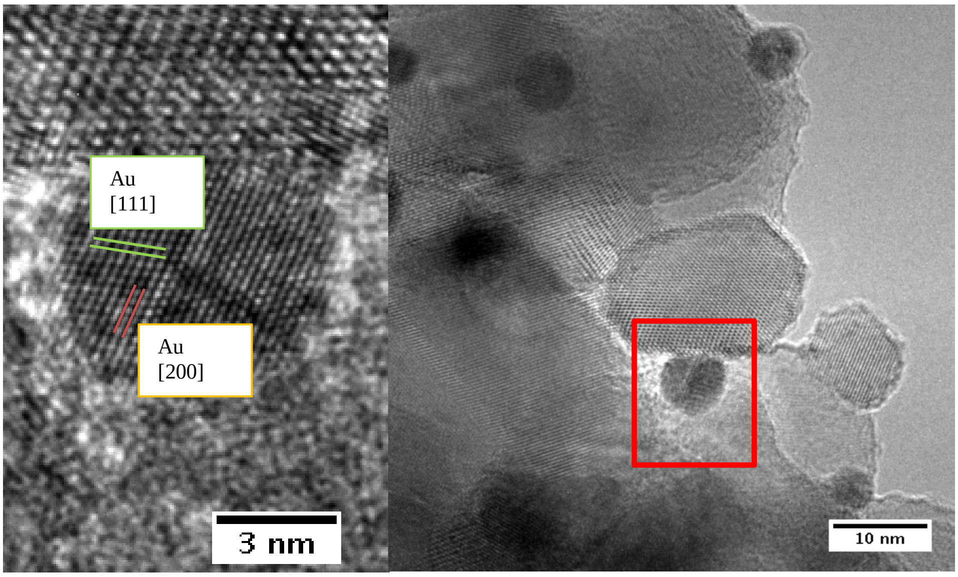

Figure S5. Left: High-resolution (HR) BFTEM image of an Au NP in Au (5 wt %)/TiO_2. Lattice fringes are consistent with pristine metallic [111] and [200] lattice parameters (scale bar: 3 nm). Right: lower magnification BFTEM image highlighting the particle studied by HRTEM (Scale bar: 10 nm).

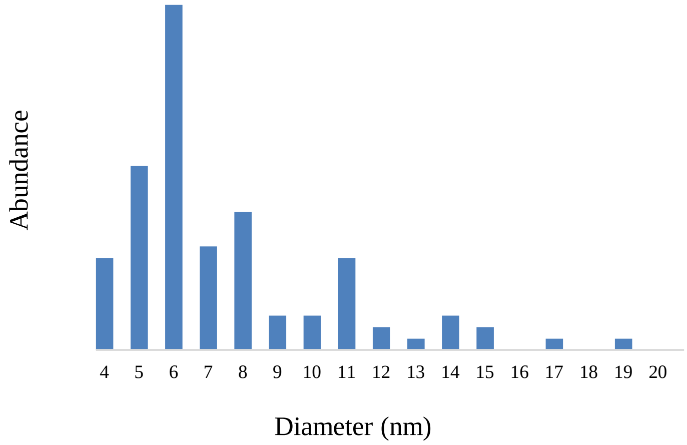

Figure S6. Particle size distribution for the Au NPs in Au (5 wt %)/TiO_2. Mean particle size = 7.65 ± 0.46 nm. SD = 3.53. N = 100.

Pristine mixed Cu/TiO_2 + Au/TiO_2

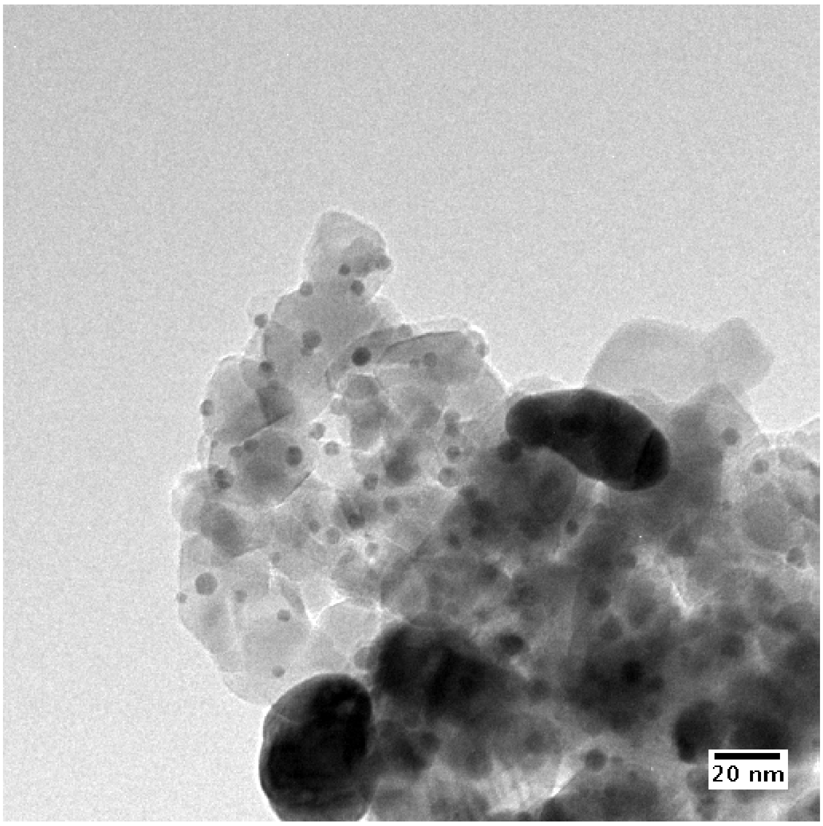

Figure S7. Representative BFTEM imaging of a pristine mixed photocatalyst system before reaction on a lacey carbon Cu grid (scale bar: 20 nm).

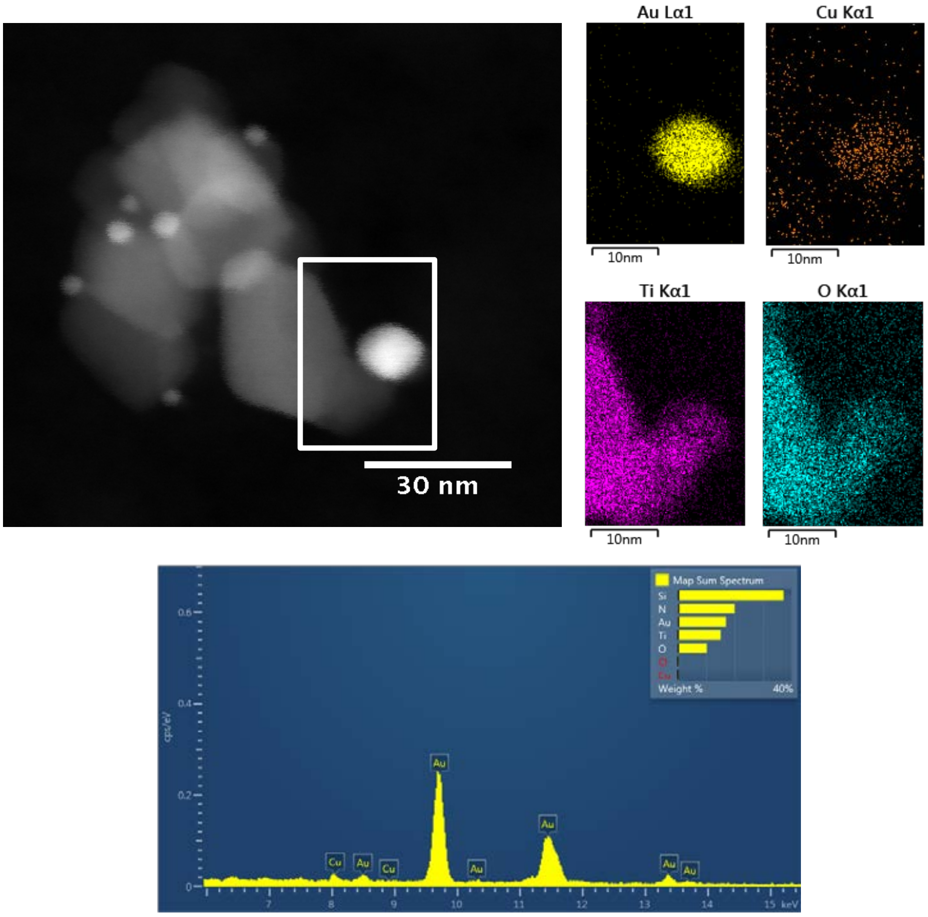

Figure S8. Top left: Representative STEM-HAADF image of a pristine mixed photocatalyst system before reaction with the white box indicating the scanning area (scale bar: 30 nm). Top right: EDS maps of the selected area (scale bar: 10 nm). Bottom: EDS spectrum of the selected area.

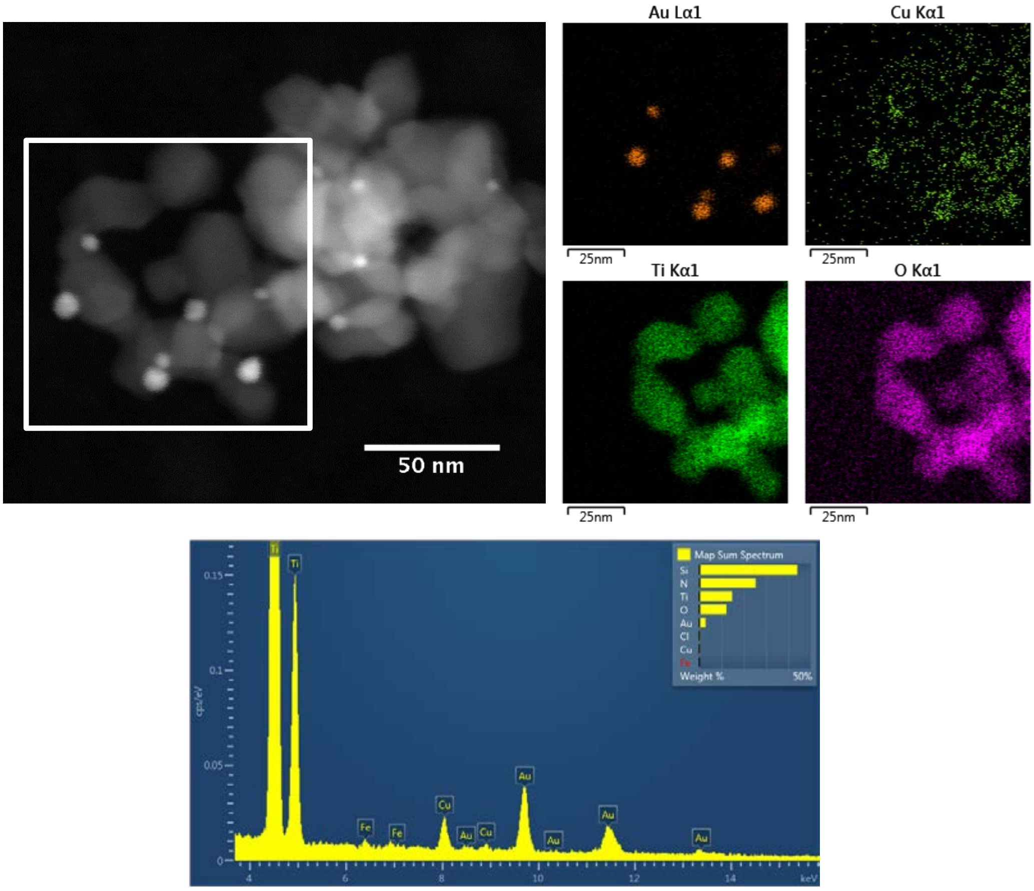

Figure S9. Top left: Representative STEM-HAADF image of a pristine mixed photocatalyst system before reaction with the white box indicating the scanning area (scale bar: 50 nm). Top right: EDS maps of the selected area (scale bar: 25 nm). Bottom: EDS spectrum of the selected area.

Mixed Cu/TiO_2 + Au/TiO_2 after 1 h reaction

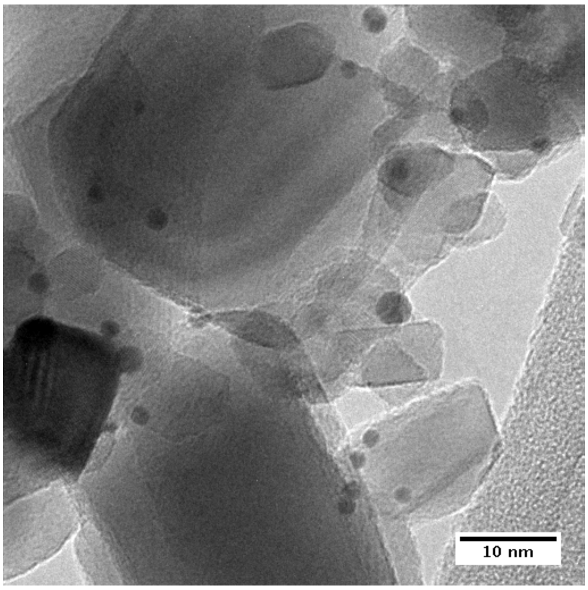

Figure S10. Representative BFTEM image of the mixed photocatalyst system after reaction for 1 h on a lacey carbon Cu grid (scale bar: 10 nm).

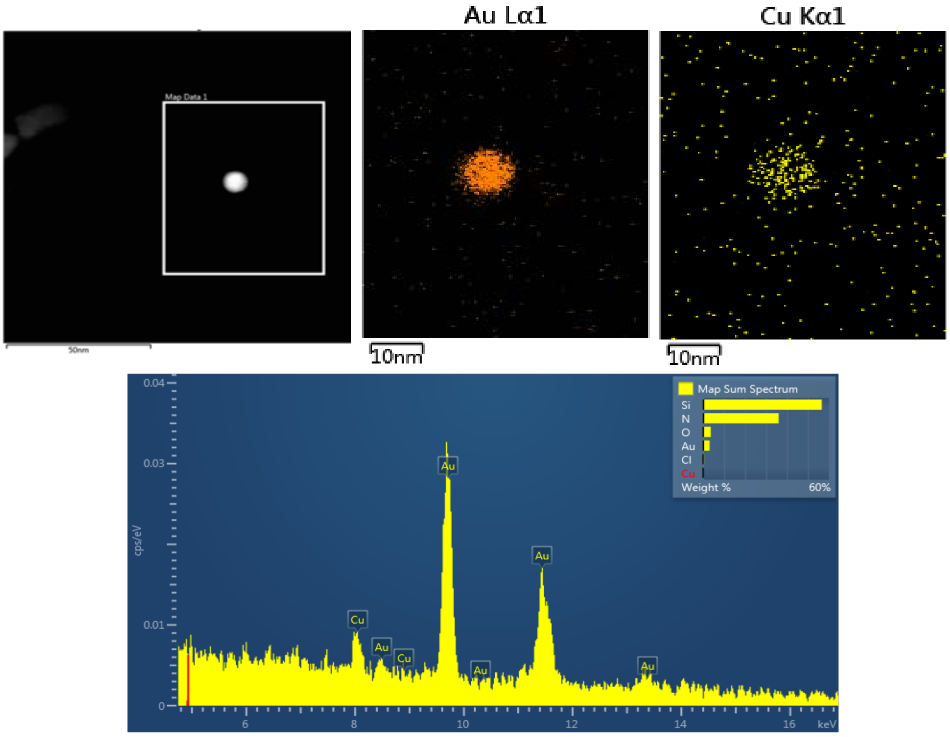

Figure S11. Top left: Representative STEM-HAADF image of the mixed photocatalyst system after reaction for 1 h with the white box indicating the scanning area (scale bar: 50 nm). Top middle and right: EDS maps of the selected area (scale bar: 10 nm). Bottom: EDS spectrum of the selected area.

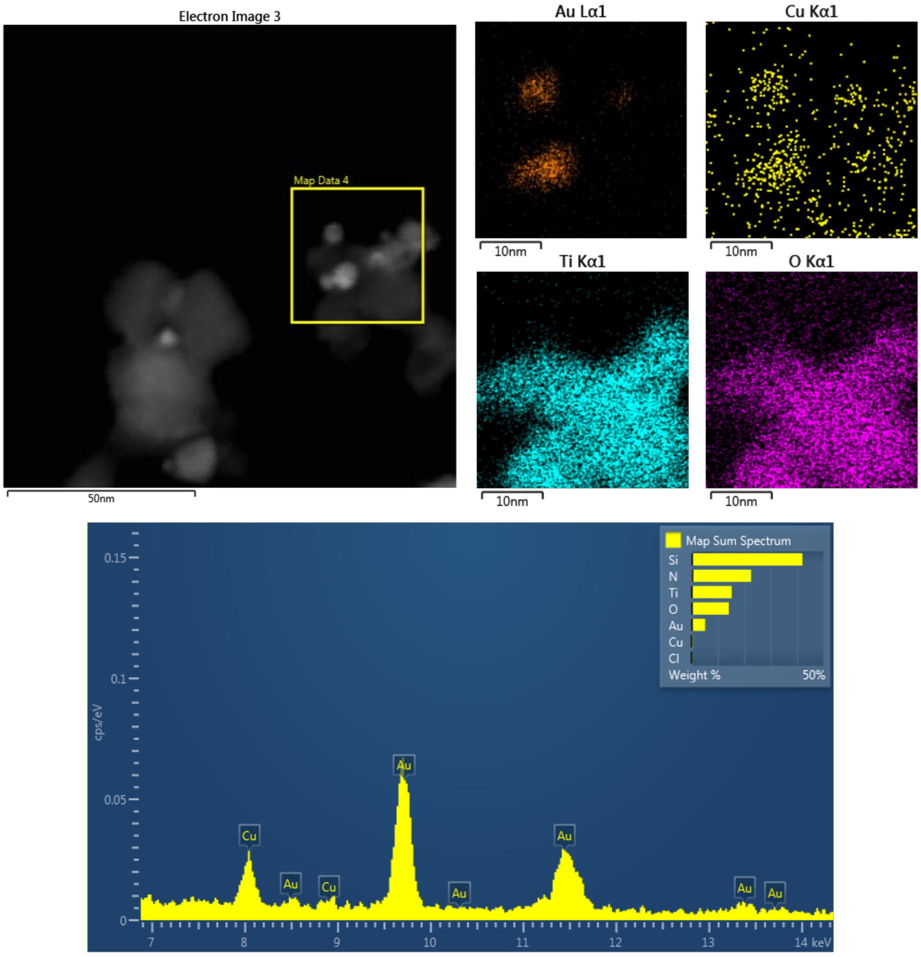

Figure S12. Top left: Representative STEM-HAADF image of the mixed photocatalyst system after reaction for 1 h with the white box indicating the scanning area (scale bar: 50 nm). Top middle and right: EDS maps of the selected area (scale bar: 10 nm). Bottom: EDS spectrum of the selected area.

Mixed Cu/TiO_2 + Au/TiO_2 after 3 h reaction

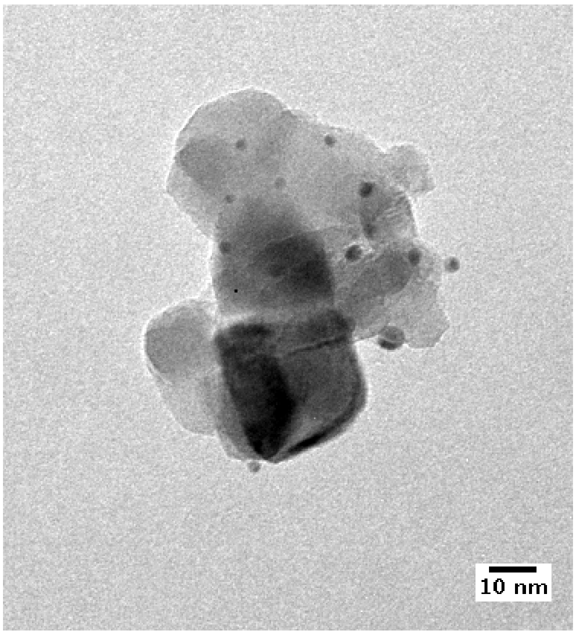

Figure S13. Representative BFTEM image of the mixed photocatalyst system after reaction for 3 h on a lacey carbon Cu grid (scale bar: 10 nm).

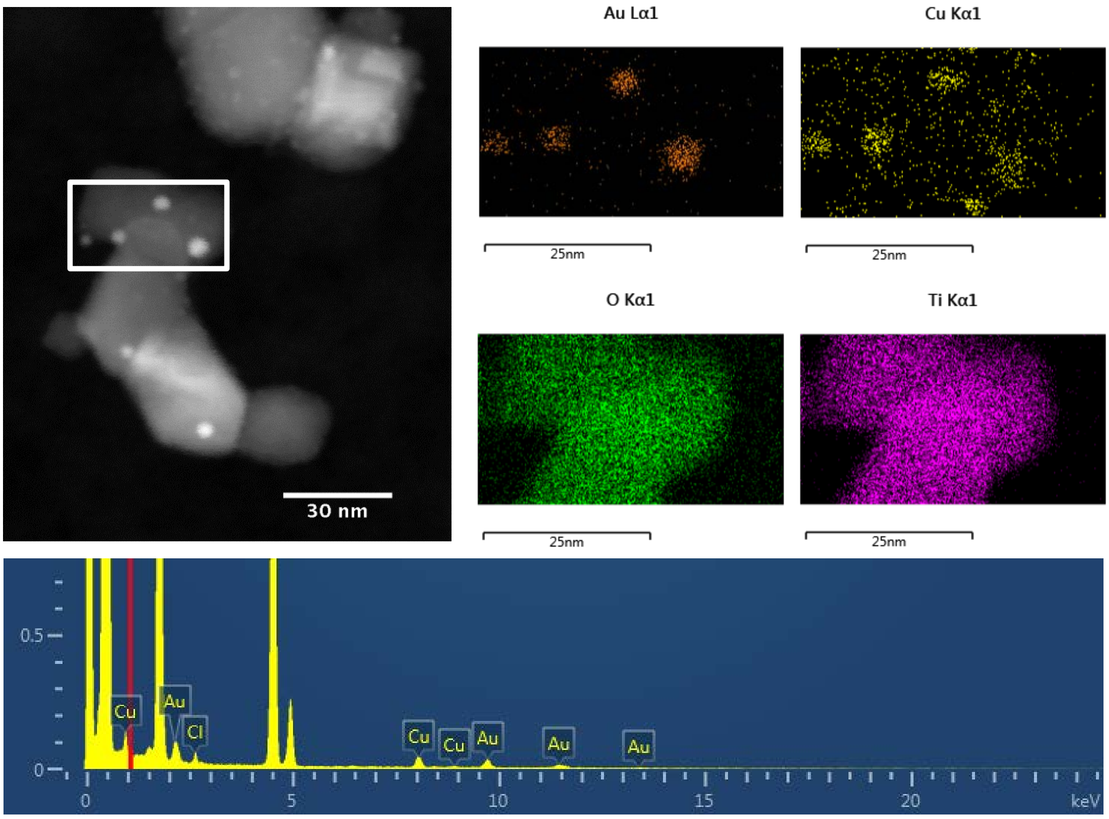

Figure S14. Top left: Representative STEM-HAADF image of the mixed photocatalyst system after reaction for 3 h with the white box indicating the scanning area (scale bar: 30 nm). Top middle and right: EDS maps of the selected area (scale bar: 25 nm). Bottom: EDS spectrum of the selected area.

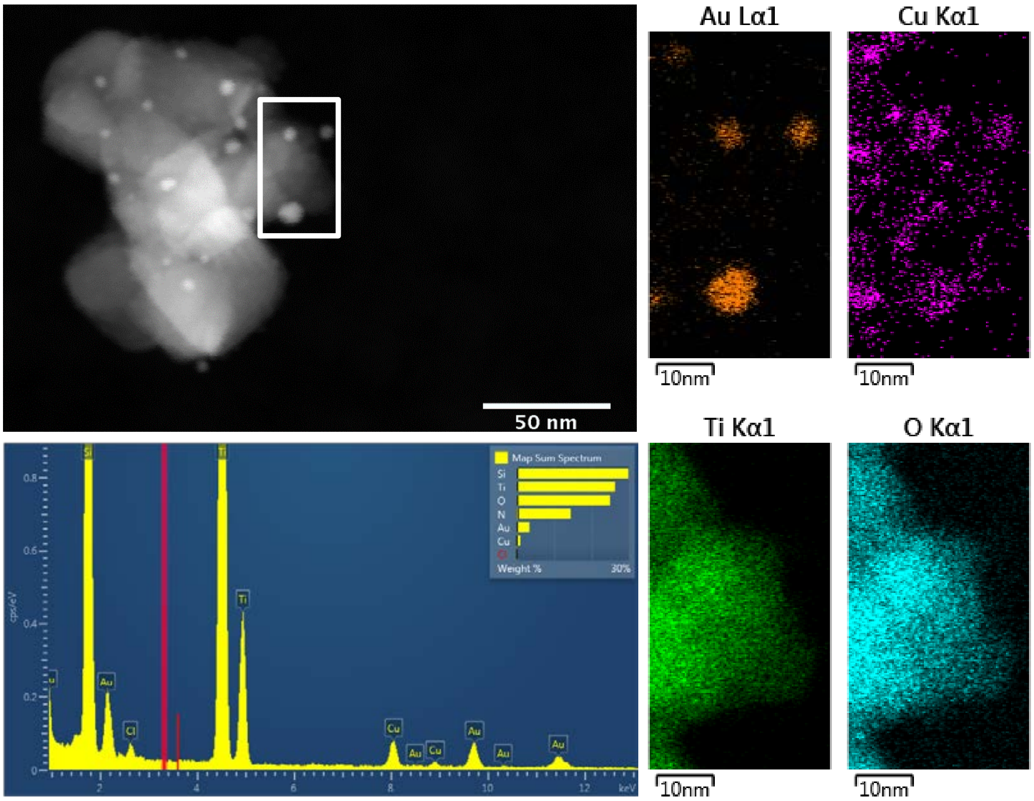

Figure S15. Top left: Representative STEM-HAADF image of the mixed photocatalyst system after reaction for 3 h with the white box indicating the scanning area (scale bar: 50 nm). Right: EDS maps of the selected area (scale bar: 10 nm). Bottom left: EDS spectrum of the selected area.

Mixed Cu/TiO$_2$ + Au/TiO$_2$ after 20 h reaction

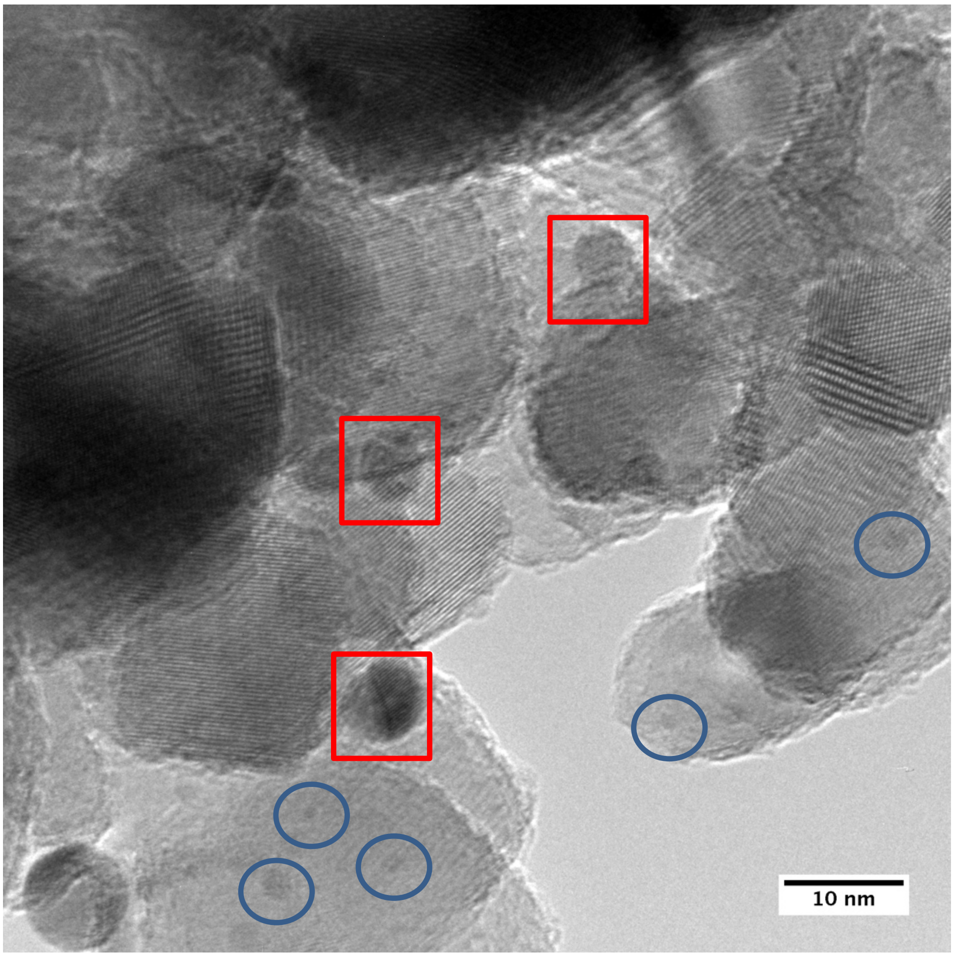

Figure S16. Representative BFTEM image of the mixed photocatalyst system after reaction for 20 h on a lacey carbon Cu grid (scale bar: 10 nm; ○ Cu NPs and □ Au NPs).

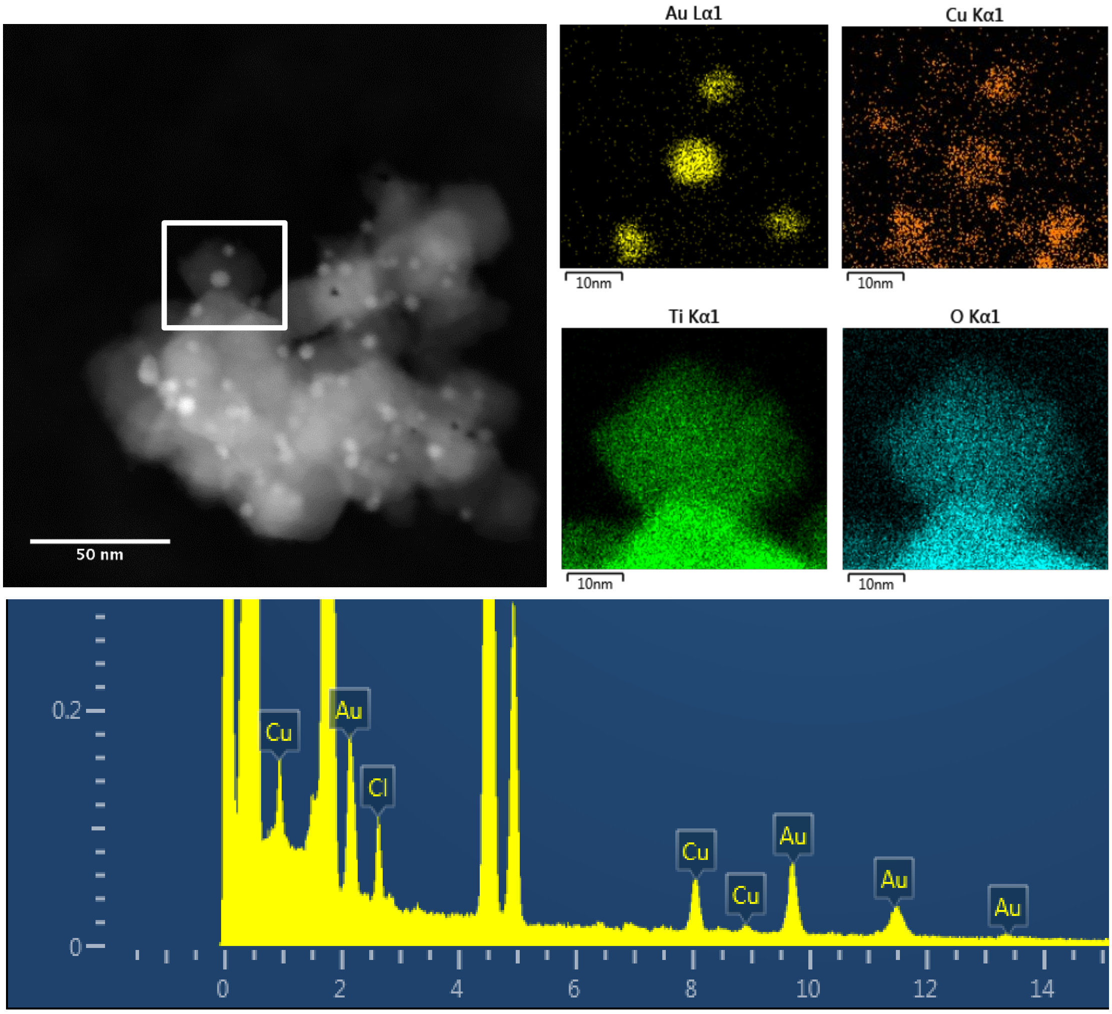

Figure S17. Top left: Representative STEM-HAADF image of the mixed photocatalyst system after reaction for 20 h with the white box indicating the scanning area (scale bar: 50 nm). Top right: EDS maps of the selected area (scale bar: 10 nm). Bottom: EDS spectrum of the selected area.

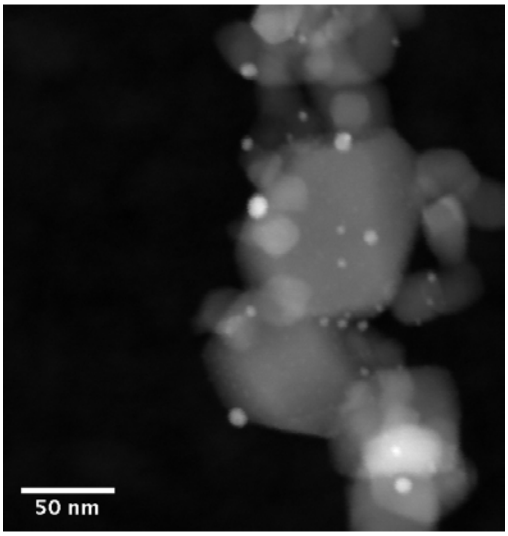

Figure S18. Representative STEM-HAADF image of the mixed photocatalyst system after reaction for 20 h showing both bright Au NPs and less bright Cu NPs (scale bar: 50 nm).

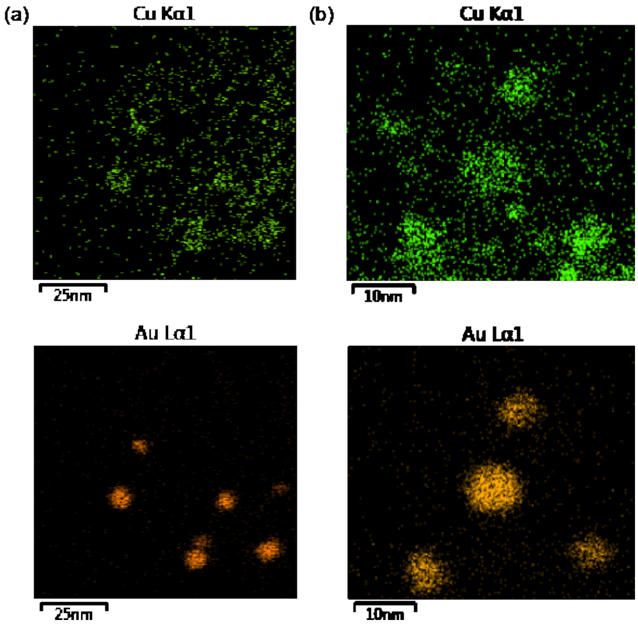

Figure S19. STEM EDS images that compare Cu and Au distributions in (a) a fresh mixture of Cu/TiO_2 and Au/TiO_2 (see Figure S9), and (b) a mixture of Cu/TiO_2 and Au/TiO_2 after photocatalytic reaction of **1c** with methanol for 20 h (see Figure S17).

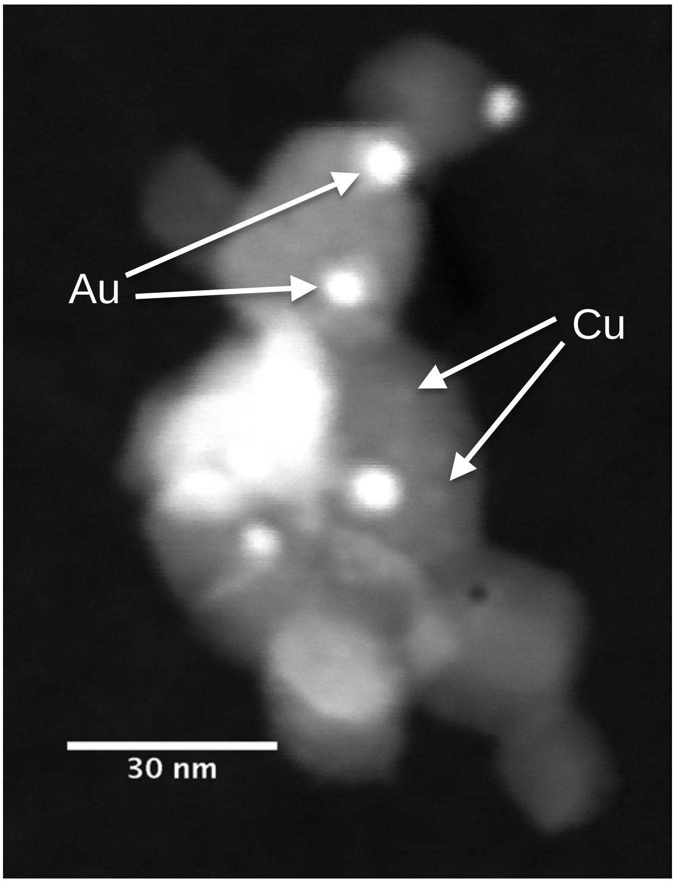

Figure S20. Representative STEM-HAADF image of the Cu–Au mixed photocatalyst system after stirring under UV irradiation for 20 h in methanol in the absence of amine substrate (scale bar: 30 nm).

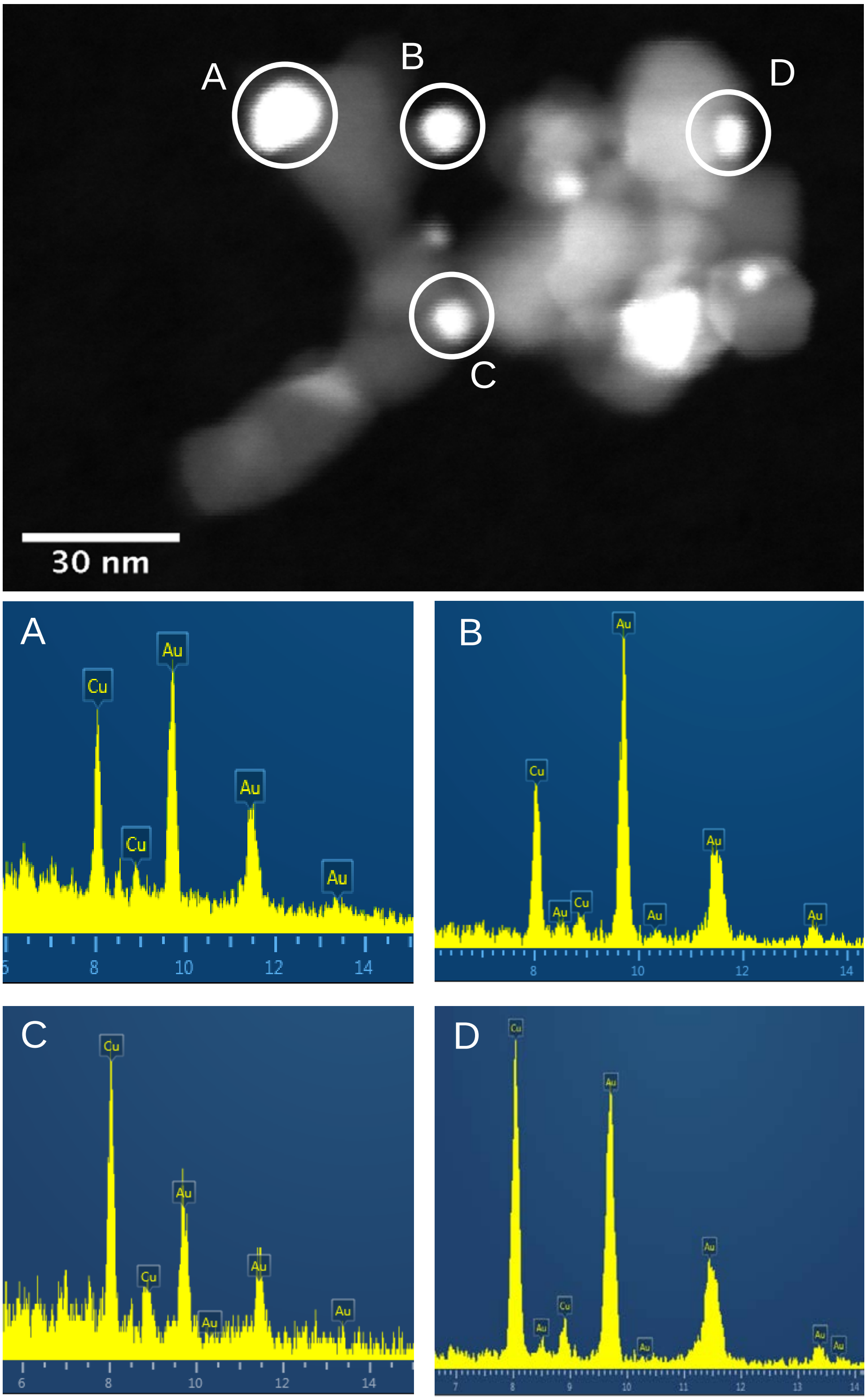

Figure S21. Top: Representative STEM-HAADF image of the Cu–Au mixed photocatalyst stirred under UV irradiation for 20 h in methanol in the absence of amine substrate (scale bar: 30 nm). Bottom: EDX spectra corresponding to the areas marked on STEM-HAADF image. In each of A-D, the EDX compositions were calculated to be $Au_{0.50}Cu_{0.50}$, $Au_{0.62}Cu_{0.38}$, $Au_{0.33}Cu_{0.66}$ and $Au_{0.44}Cu_{0.56}$, respectively.

5.2 Powder X-ray diffraction

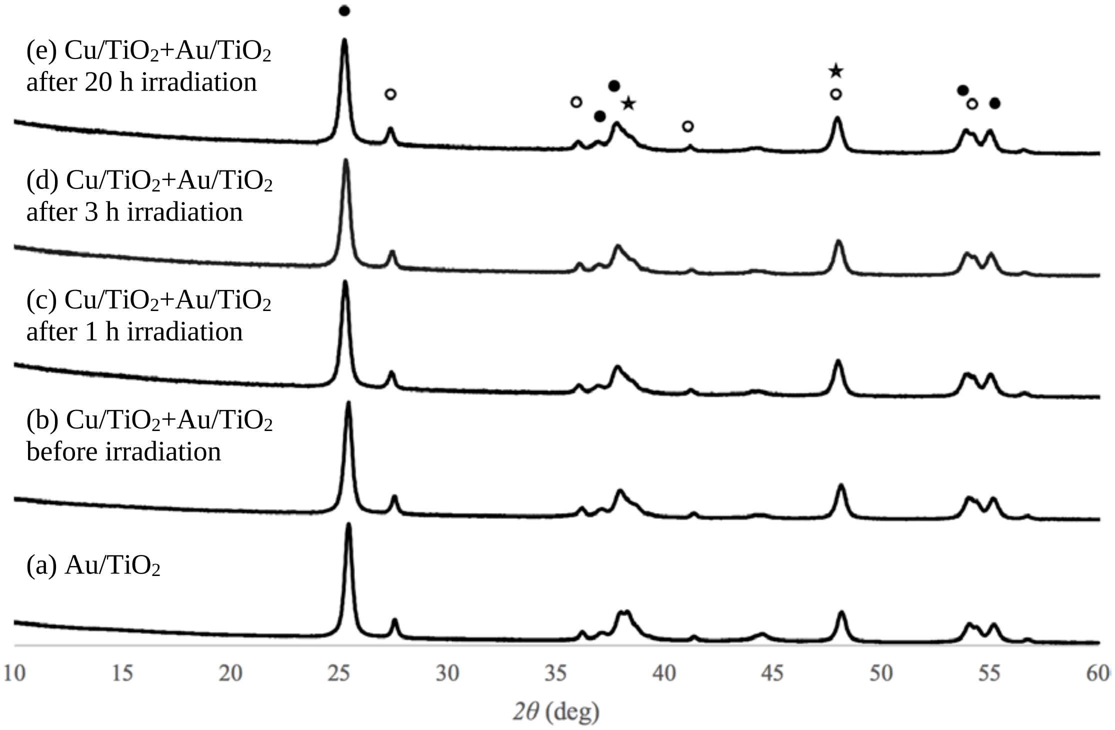

Figure S22. PXRD patterns for: monometallic Au (5 wt %)/TiO_2 (a) and Cu–Au mixed photocatalyst [Cu (5 wt %)/TiO_2 + Au (5 wt %)/TiO_2] before (b), after 1 h (c), after 3 h (d), and after 20 h (e) reaction of **1c** with methanol. ● = anatase (TiO_2), ○ = rutile (TiO_2), ★ = gold.

6. Photocatalytic N-Methylation of 1a to (*rac*)-Rivastigmine (3aa)

A representative procedure for N-methylation of 1a to 3aa by Cu (5 wt %)/TiO_2 and Au (5 wt %)/TiO_2 (Figure 2, entry 13)

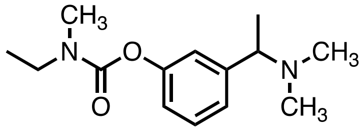

Cu (5 wt %)/TiO_2 (22 mg, 1.6 mol % Cu), Au (5 wt %)/TiO_2 (22 mg, 0.51 mol % Au), anhydrous CH_3OH (10 mL, 250 mmol), and **1a** (222.1 mg, 1.00 mmol) were added successively to a cylindrical Pyrex glass reaction vessel (diameter: 50 mm, height: 130 mm with a top window made of Pyrex) connected to a rubber balloon. After the resulting mixture was sonicated for 30 sec and deaerated by Ar bubbling *via* cannula for 5 min, the vessel was immersed in a water bath (kept at 25 ºC using a cooling circulator), and stirred for 4 h with irradiation [300 W Xe lamp (Ushio: BA-x300/ES1 Technology; CERMAX PE300BF) equipped with a UV cold mirror (λ = 300–470 nm)]. The presence of **3aa** (95% yield) in the reaction mixture was indicated by GC/MS analysis using 2,2-dimethylpropan-1-ol as an internal standard. The reaction mixture was filtered through 0.45 µm membrane filter and the photocatalyst was washed with CH_3OH (10 mL). HCl (35–37%, 12 M aq, 0.5 mL, 6 mmol) was added to the solution (pH 1–2) and stirred at rt for 30 min. After methanol was evaporated, the residue was dissolved in H_2O (20 mL) and washed with ethyl acetate (20 mL). To the aqueous layer, sodium carbonate (s) was added (pH 10) and extracted with ethyl acetate (2 × 30 mL). After washed with brine, the organic layer was dried over Na_2SO_4 and concentrated under reduced pressure to afford (*rac*)-rivastigmine (**3aa**) as a light-yellow oil (227.8 mg, 91% yield). Analytical data for **3aa** (observed as a 1:1 *cis*/*trans* isomeric mixture around the carbamate moiety): ^{1}H NMR (500 MHz, $CDCl_3$) δ 1.18 (t, J = 6.9 Hz, 1.5H), 1.23 (t, J = 6.9 Hz, 1.5H), 1.35 (d, J = 6.5 Hz, 3H), 2.20 (s, 6H), 2.98 (s, 1.5H), 3.05 (s, 1.5H), 3.24 (q, J = 6.5 Hz, 1H), 3.40 (q, J = 6.9 Hz, 1H), 3.46 (q, J = 7.1 Hz, 1H), 7.00 (d, J = 7.5 Hz, 1H), 7.06 (s, 1H), 7.11 (d, J = 7.5 Hz, 1H), 7.28 (t, J = 7.5 Hz, 1H); ^{13}C{^{1}H} NMR (125 MHz, $CDCl_3$) δ 12.6 (0.5C), 13.3 (0.5C), 20.2, 33.9 (0.5C), 34.3 (0.5C), 43.3, 44.1, 65.7, 120.3, 120.8, 124.3, 129.0, 145.9, 151.6, 154.5 (0.5C), 154.6 (0.5C); HRMS (ESI) calcd for $C_{14}H_{22}N_2O_2H^+$ ([M + H]$^+$) 251.1754, found 251.1760; calcd for $C_{14}H_{22}N_2O_2Na^+$ ([M + Na]$^+$) 273.1573, found 273.1576. These data are consistent with the literature values.[7]

N-Methylation of (*S*)-1a to (*S*)-3aa by Cu/TiO_2 and Au/TiO_2 (Figure 3)

Photocatalytic N-methylation of (*S*)-**1a** to (*S*)-**3aa** was carried out by following the above-mentioned representative procedure (25 °C, 5 h) to afford (*S*)-**3aa** (228 mg, 91% yield); $[\alpha]^{22}_{D}$ –29.7 (*c* 1.3, C_2H_5OH) [lit.[8] $[\alpha]^{25}_{D}$ –32.8 (*c* 1.3, C_2H_5OH) (97% *ee*)]; The *S*:*R* enantiomeric ratio was determined by HPLC analysis (Daicel Chiralcel OD-H, hexane/ethanol/trifluoroacetic acid/diethylamine = 92:8:0.2:0.1, 1 mL/min, 30 °C, 220 nm), t_R = 15.24 min (*R*), 22.84 min (*S*), *S*:*R* ratio = 96:4.

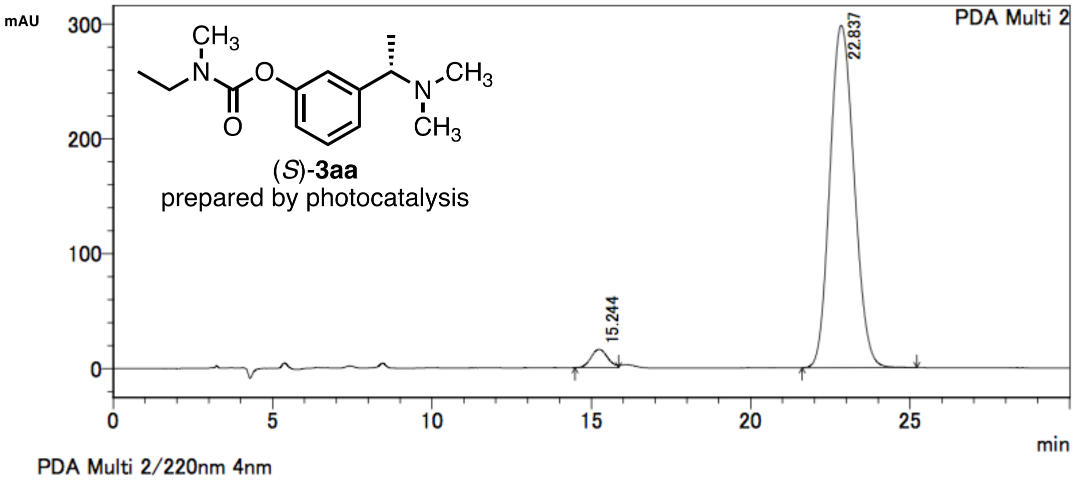

The *S*:*R* enantiomeric ratio of (*S*)-**1a** was deduced by determining that for (*S*)-**3aa** synthesized by reductive amination of (*S*)-**1a** employing HCHO and $NaBH(OAc)_3$.[2] HPLC (Daicel Chiralcel OD-H, hexane/ethanol/trifluoroacetic acid/diethylamine = 92:8:0.2:0.1, 1 mL/min, 30 °C, 220 nm), t_R = 15.23 min (*R*), 22.87 min (*S*), *S*:*R* ratio = 96:4.

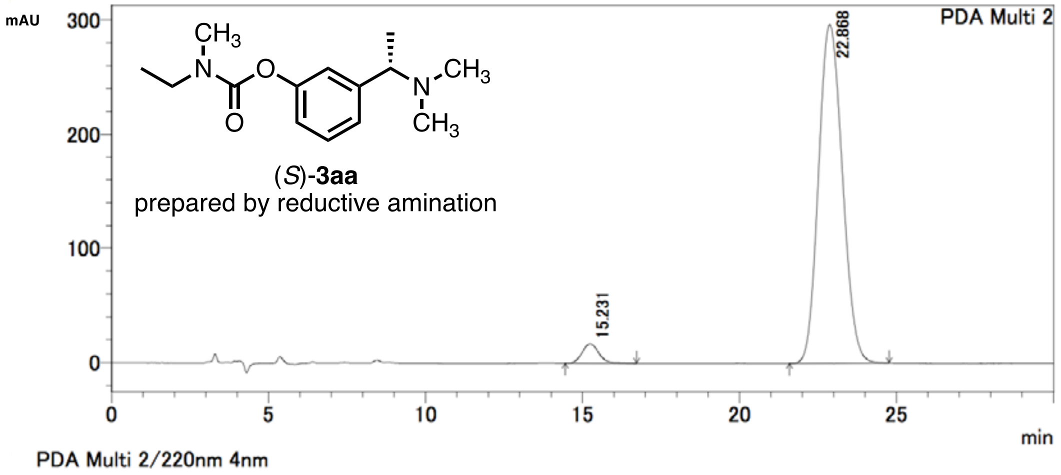

HPLC analysis of (*rac*)-**3aa** (Daicel Chiralcel OD-H, hexane/ethanol/trifluoroacetic acid/diethylamine = 92:8:0.2:0.1, 1 mL/min, 30 °C, 220 nm), t_R = 14.75 min (*R*), 22.58 min (*S*). *S*:*R* ratio = 49:51.

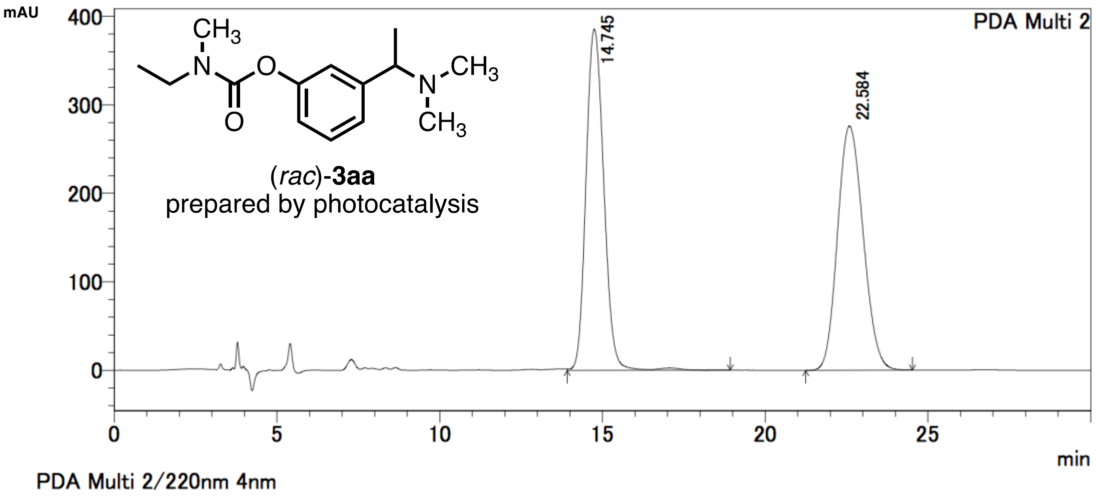

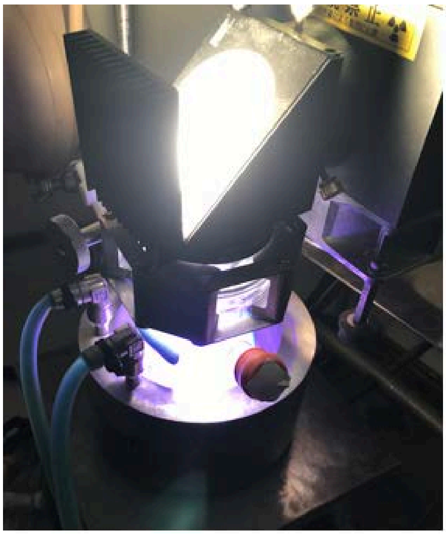

Irradiation of a reaction vessel

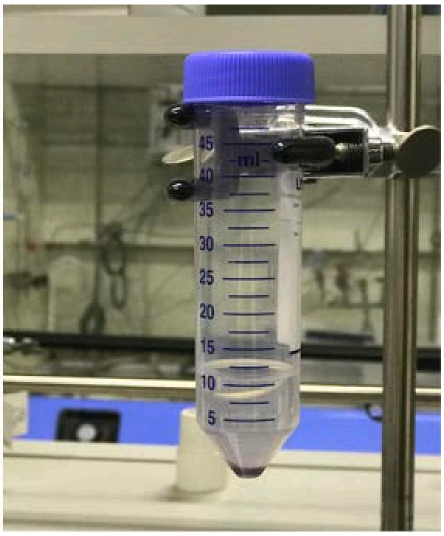

Reaction mixture after centrifugation

Table S2. Photocatalyst screening for N,N-dimethylation of **1a** to **3aa**[a]

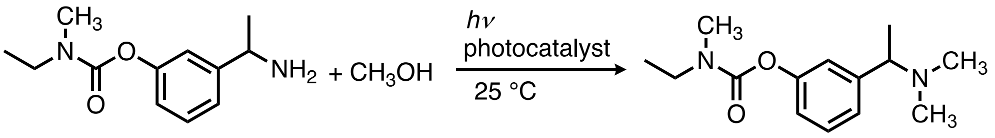

Photocatalyst[b]	metal (mol %)[b]	time (h)	conv. of **1a** (%)[c]	yield of **3aa** (%)[c]
Au (5 wt %)/TiO_2	Au (0.51)	7	> 99	5[d]
Ag (4 wt %)/TiO_2	Ag (0.85)	7	> 99	68
Cu (5 wt %)/TiO_2	Cu (1.6)	7	> 99	92 [89][e]
Pd (5 wt %)/TiO_2	Pd (0.95)	7	99	65
Pt (5 wt %)/TiO_2	Pt (0.32)	7	> 99	1[d]
TiO_2	-	5	67	< 1

[a]Typical conditions: photocatalyst 22 mg, **1a** (1.0 mmol), methanol (250 equiv, 10 mL), 300 W Xe lamp with UV-cold mirror (λ = 300−470 nm), Ar (1 atm), 25 °C, 7 h unless otherwise noted. [b]Metal content was determined by ICP-AES. [c]GC/MS yields using 2,2-dimethylpropan-1-ol as an internal standard. [d]^{1}H-NMR analysis using 2,2-dimethylpropan-1-ol as an internal standard. [e]Isolated yield of **3aa** as HCl salt.

Table S3. Effect of Cu loading in N,N-dimethylation of **1a** to **3aa** using Cu/TiO_2[a]

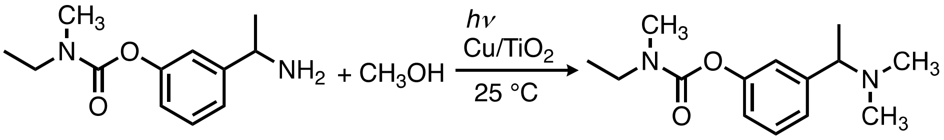

		yield of **3aa** (%)[c]			
Cu/TiO_2[b]	metal (mol %)[b]	3 h	4 h	5 h	6 h
Cu (1 wt %)/TiO_2	Cu (0.32)	32	54	61	84
Cu (5 wt %)/TiO_2	Cu (1.6)	68	86	90	92
Cu (11 wt %)/TiO_2	Cu (3.5)	54	82	89	92

[a]Typical conditions: Cu/TiO_2 22 mg, **1a** (1.0 mmol), methanol (250 equiv, 10 mL), 300 W Xe lamp with UV-cold mirror (λ = 300−470 nm), Ar (1 atm), 25 °C, 3–6 h unless otherwise noted. [b]Metal content was determined by ICP-AES. [c]GC/MS yields using 2,2-dimethylpropan-1-ol as an internal standard.

Table S4. Relative reactivity of photocatalysts based on conversion of **1a**[a]

photocatalyst[b]	metal (mol %)[b]	conditions[c]	relative reactivity (%)	conv. of **1a** (%)[d]
Ag (4 wt %)/TiO_2	Ag (0.85)	A	58	54
Cu (5 wt %)/TiO_2	Cu (1.6)	A	64	60
Pd (5 wt %)/TiO_2	Pd (0.95)	A	78	74
Pt (5 wt %)/TiO_2	Pt (0.32)	A	80	75
Au (5 wt %)/TiO_2	Au (0.51)	A	100	93
Au (5 wt %)/TiO_2	Au (0.51)	B	100	69
Au (1 wt %)/TiO_2	Au (0.092)	B	83	57
Au (0.6 wt %)/TiO_2	Au (0.062)	B	75	52
Au (6 wt %)/TiO_2	Au (0.54)	B	64	44

[a]Conditions: photocatalyst 22 mg, **1a** (1.0 mmol), methanol (250 equiv, 10 mL), 300 W Xe lamp with UV-cold mirror (λ = 300−470 nm), Ar (1 atm), 25 °C, 1 h unless otherwise noted. [b]Metal content was determined by ICP-AES. [c]Irradiated with A: new Xe lamp or B: old Xe lamp with decreased light intensity. [d]GC/MS yields using 2,2-dimethylpropan-1-ol as an internal standard.

Table S5. Reactivity comparison of different mixed photocatalysts in N,N-dimethylation of **1a** to **3aa**[a]

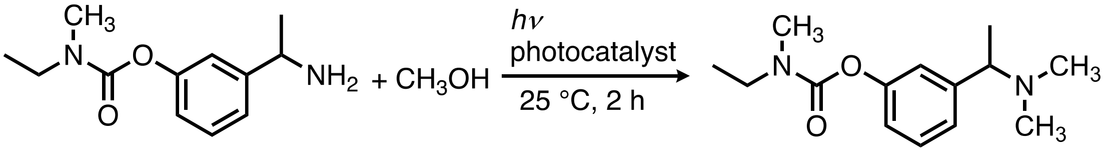

photocatalyst	metal (mol %)[b]	yield (%)[c]
Cu/TiO_2, Au/TiO_2	1.6, 0.51	70
Cu/TiO_2 (43 mg)	3.2	40
Cu/TiO_2, Pd/TiO_2	1.6, 0.95	50
Cu/TiO_2, Ag/TiO_2	1.6, 0.85	42
Cu/TiO_2, Pt/TiO_2	1.6, 0.32	59

[a]Typical conditions: Cu/TiO_2 22 mg, Metal/TiO_2 22 mg, **1a** (1.0 mmol), methanol (250 equiv, 10 mL), 300 W Xe lamp with UV-cold mirror (λ = 300−470 nm), Ar (1 atm), 25 °C, 2 h unless otherwise noted. [b]Metal content was determined by ICP-AES. [c]GC/MS or ^{1}H NMR yields using 2,2-dimethylpropan-1-ol as an internal standard.

7. Photocatalytic N-Alkylation of Amines

A general procedure for N-monoalkylation of amines (A)

As per the above-mentioned representative procedure for N-methylation of **1a**, N-monoalkylation of amines (0.2 mmol) was conducted using Au (5 wt %)/TiO_2 (10 mg, 1.2 mol % Au), Cu (5 wt %)/TiO_2 (10 mg, 3.6 mol % Cu), alcohol (0.5 mL), and hexane (10 mL) for 0.8–1.5 h. The reaction progress was analyzed by GC/MS. After the photocatalyst was removed, the photocatalyst was washed with CH_3OH (5 mL). The combined organic layer was concentrated under reduced pressure before CH_3OH (10 mL) and HCl (2 M in diethylether, 0.2 mL, 0.4 mmol) was added to the solution and stirred at rt for 30 min. The mixture was concentrated under reduced pressure, the residue was dissolved in $CHCl_3$ (0.5 mL) and added to diethyl ether (10 mL) to form precipitates. The precipitate was isolated by centrifugation (10 min) and dried at 25 °C for 10 h to give amines **2**.

A general procedure for N-monoalkylation of amines (B)

As per the above-mentioned representative procedure for N-methylation of **1a**, N-monoalkylation of amines (1.0 mmol) was conducted using Au (5 wt %)/TiO_2 (22 mg, 0.51 mol % Au), Cu (5 wt %)/TiO_2 (22 mg, 1.6 mol % Cu), alcohol (2–4 equiv), and CPME (10 mL) for 13–20 h. The reaction progress was analyzed by GC/MS. After the photocatalyst was removed, and the photocatalyst was washed with CH_3OH (10 mL). The combined organic layer was concentrated under reduced pressure, and chromatographic purification was conducted to isolate **2** as a free amine.

A general procedure for N-alkylation of amines (C)

As per the above-mentioned representative procedure for N-methylation of **1a**, N-alkylation of amines (1.0 mmol) was conducted using Au (5 wt %)/TiO_2 (22 mg, 0.51 mol % Au), Cu (5 wt %)/TiO_2 (22 mg, 1.6 mol % Cu), and alcohol (10 mL) for 2.5–18 h. The reaction progress was analyzed by GC/MS. After the photocatalyst was removed, the photocatalyst was washed with CH_3OH (5 mL). The combined organic layer was concentrated under reduced pressure to give products **2** or **3**. When the purity is unsatisfactory, the product was further purified by forming a HCl salt (see general procedure **A**) or by column chromatography.

A general procedure for non-symmetrical N,N-dialkylation of amines (D)

As per the above-mentioned general procedure **B**, N-monoalkylation of amines (1.0 mmol) was conducted using Au (5 wt %)/TiO_2 (22 mg, 0.51 mol % Au), Cu (5 wt %)/TiO_2 (22 mg, 1.6 mol % Cu), alcohol (R^1OH, 2 equiv), and CPME (10 mL) for 13–16 h. Another alcohol (R^2OH, 5.0 mL) was then added to the reaction and the resulting mixture was irradiated for another 6–16 h. The reaction progress was analyzed by GC/MS. The product was purified as a free amine in the same way shown in general procedure **B**.

***N*-Ethyl-*α*-methylbenzylamine (2bb•HCl, Figure 3)**

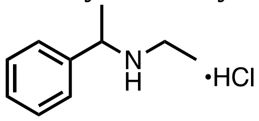

Prepared according to general procedure **A** using *α*-methylbenzylamine (**1b**, 24.3 mg, 0.20 mmol) for 50 min. **2bb**•HCl was obtained as a white powder (32 mg, 87% yield). Analytical data for **2bb**•HCl: Mp 200.2–201.3 °C (lit.[9] 198–199 °C); ^{1}H NMR (500 MHz, $CDCl_3$) *δ* 1.44 (t, *J* = 7.6 Hz, 3H), 1.91 (d, *J* = 6.9 Hz, 3H), 2.71–2.85 (m, 2H), 4.22 (t, *J* = 6.5 Hz, 1H), 7.39 (t, *J* = 7.6 Hz, 1H), 7.44 (t, *J* = 7.6 Hz, 2H), 7.64 (d, *J* = 8.3 Hz, 2H), 9.84 (s, 1H), 10.17 (s, 1H); ^{13}C{^{1}H} NMR (125 MHz, $CDCl_3$) *δ* 11.3, 20.8, 40.9, 58.7, 127.9, 129.3, 129.5, 136.2; HRMS (ESI) calcd for $C_{10}H_{15}NH^+$ ($[M – Cl]^+$) 150.1277, found 150.1282.

***N*-Propyl-*α*-methylbenzylamine (2bc•HCl, Figure 3)**

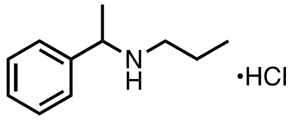

Prepared according to general procedure **A** using *α*-methylbenzylamine (**1b**, 24.4 mg, 0.20 mmol) for 90 min. **2bc**•HCl was obtained as a light-yellow powder (38 mg, 95% yield). Analytical data for **2bc**•HCl: Mp 170.4–172.6 °C (lit.[10] 177 °C); ^{1}H NMR (500 MHz, $CDCl_3$) *δ* 0.88 (t, *J* = 6.9 Hz, 3H), 1.70–2.09 (m, 5H), 2.63 (s, 2H), 4.22 (s, 1H), 7.29–7.49 (m, 3H), 7.64 (d, *J* = 6.9 Hz, 2H), 9.75 (s, 1H), 10.09 (s, 1H); ^{13}C{^{1}H} NMR (125 MHz, $CDCl_3$) *δ* 11.5, 19.5, 20.8, 47.6, 59.1, 128.0, 129.4, 129.5, 136.2; HRMS (ESI) calcd for $C_{11}H_{17}NH^+$ ($[M – Cl]^+$) 164.1434, found 164.1441.

***N*-(Cyclopropylmethyl)-*α*-methyl-benzenemethanamine (2bd, Figure 3)**

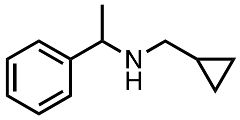

Prepared according to general procedure **B** using *α*-methylbenzylamine (**1b,** 121.8 mg, 1.01 mmol), and cyclopropanemethanol (217.5 mg, 3.02 mmol) for 20 h. The product was purified by column chromatography on NH silica gel (hexane/ethyl acetate 10:1) to afford **2bd** as a yellow oil (147 mg, 84% yield). Analytical data for **2bd**: ^{1}H NMR (500 MHz, $CDCl_3$) *δ* 0.02–0.12 (m, 2H), 0.43–0.53 (m, 2H), 1.40 (d, *J* = 6.9 Hz, 3H), 1.60 (bs, 1H), 2.27 (q, *J* = 6.3 Hz, 1H), 2.43 (q, *J* = 6.3 Hz, 1H), 3.82 (q, *J* = 6.5 Hz, 1H), 7.24–7.29 (m, 1H), 7.32–7.38 (m, 4H); ^{13}C{^{1}H} NMR (125 MHz, $CDCl_3$) *δ* 3.3, 3.6, 11.5, 24.6, 53.0, 58.3, 126.6, 126.9, 128.5, 145.9; HRMS (ESI) calcd for $C_{12}H_{17}NH^+$ ($[M + H]^+$) 176.1434, found 176.1442.

***N*-(Cyclobutylmethyl)-*α*-methylbenzenemethanamine (2be, Figure 3)**

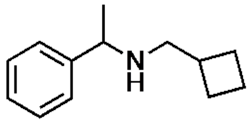

Prepared according to general procedure **B** using *α*-methylbenzylamine (**1b,** 121.7 mg, 1.00 mmol), and cyclobutanemethanol (345 mg, 4.01 mmol) for 20 h. The product was purified by column chromatography on NH silica gel (hexane/ethyl acetate 10:1) to afford **2be** as a light-yellow oil (164 mg, 86% yield). Analytical data for **2be**: ^{1}H NMR (500 MHz, $CDCl_3$) *δ* 1.34 (d, *J* = 6.9 Hz, 3H), 1.41 (bs, 1H), 1.53–1.64 (m, 2H), 1.75–1.92 (m, 2H), 2.00–2.01 (m, 2H), 2.40–2.46 (m, 2H), 2.51–2.55 (m, 1H), 3.75 (q, *J* = 6.7 Hz, 1H), 7.20–7.25 (m, 1H), 7.29–7.33 (m, 4H); ^{13}C{^{1}H} NMR (125 MHz, $CDCl_3$) *δ* 18.7, 24.6, 26.5, 35.9, 54.1, 58.5, 126.7, 126.9, 128.5, 146.0; HRMS (ESI) calcd for $C_{13}H_{19}NH^+$ ($[M + H]^+$) 190.1590, found 190.1599.

8-Chloro-*N*-(1-phenylethyl)octan-1-amine (2bf, Figure 3)

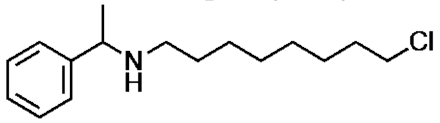

Prepared according to general procedure **B** using *α*-methylbenzylamine (**1b,** 121.4 mg, 1.00 mmol), and 8-chloro-1-octanol (331.2 mg, 2.01 mmol) for 16 h. The product was purified by column chromatography on NH silica gel (hexane/ethyl acetate 10:1) to afford **2bf** as a yellow oil (221 mg, 79% yield). Analytical data for **2bf**: ^{1}H NMR (500 MHz, $CDCl_3$) *δ* 1.27 (s, 6H), 1.35 (d, *J* = 6.9 Hz, 3H), 1.38–1.53 (m, 5H), 1.74 (quint, *J* = 7.0 Hz, 2H), 2.38–2.43 (m, 1H), 2.46–2.51 (m, 1H), 3.51 (t, *J* = 6.9 Hz, 2H), 3.75 (q, *J* = 6.7 Hz, 1H), 7.20–7.26 (m, 1H), 7.29–7.33 (m, 4H); ^{13}C{^{1}H} NMR (125 MHz, $CDCl_3$) *δ* 24.5, 26.9, 27.3, 28.9, 29.4, 30.3, 32.7, 45.2, 47.9, 58.5, 126.6, 126.9, 128.5, 145.9; HRMS (ESI) calcd for $C_{16}H_{26}NClH^+$ ($[M + H]^+$) 268.1827, found 268.1827.

***N*-[2-[2-(2-Methoxyethoxy)ethoxy]ethyl]-*α*-methyl-benzenemethanamine (2bg, Figure 3)**

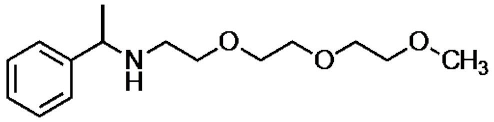

Prepared according to general procedure **B** using *α*-methylbenzylamine (**1b,** 121.7 mg, 1.00 mmol), and 2-[2-(2-methoxyethoxy)ethoxy]ethanol (329.9 mg, 2.01 mmol) at 50 °C for 13 h. The product was purified by column chromatography on NH silica gel (hexane/ethyl acetate 10:1) to afford **2bg** as a colorless oil (238 mg, 89% yield). Analytical data for **2bg**: ^{1}H NMR (500 MHz, $CDCl_3$) *δ* 1.36 (d, *J* = 6.3 Hz, 3H), 1.81 (bs, 1H), 2.56–2.62 (m, 1H), 2.65–2.72 (m, 1H), 3.37 (s, 3H), 3.50–3.66 (m, 10H), 3.76 (q, *J* = 6.5 Hz, 1H), 7.19–7.25 (m, 1H), 7.28–7.34 (m, 4H); ^{13}C{^{1}H} NMR (125 MHz, $CDCl_3$) *δ* 24.5, 47.2, 58.3, 59.1, 70.3, 70.6, 70.7, 72.0, 126.7, 126.9, 128.5, 145.7; HRMS (ESI) calcd for $C_{15}H_{25}NO_3Na^+$ ($[M + Na]^+$) 290.1727, found 290.1753.

***N*-Isopropyl-*α*-methylbenzylamine (2bh•HCl, Figure 3)**

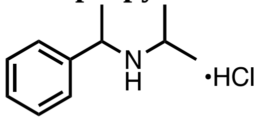

Prepared according to general procedure **C** using *α*-methylbenzylamine (**1b,** 121.6 mg, 1.00 mmol) for 16 h. The product was purified by column chromatography on silica gel (hexane/ethyl acetate/triethylamine 50:50:0.5) and by successive treatment of HCl in ether (1 mL, see general procedure A) to afford **2bh**•HCl as a white powder (142 mg, 71% yield). Analytical data for **2bh**•HCl: Mp 229.8–231.5 °C; ^{1}H NMR (500 MHz, $CDCl_3$) δ 1.38 (d, *J* = 6.2 Hz, 3H), 1.55 (d, *J* = 6.2 Hz, 3H), 1.94 (d, *J* = 6.9 Hz, 3H), 2.90–3.11 (m, 1H), 4.37 (quint, *J* = 6.9 Hz, 1H), 7.38 (t, *J* = 7.2 Hz, 1H), 7.45 (t, *J* = 7.6 Hz, 2H), 7.71 (d, *J* = 7.6 Hz, 2H), 9.69 (s, 1H), 10.00 (s, 1H); $^{13}C\{^1H\}$ NMR (125 MHz, $CDCl_3$) δ 17.8, 20.6, 21.5, 48.3, 56.4, 127.8, 129.1, 129.5, 136.5; HRMS (ESI) calcd for $C_{11}H_{17}NH^+$ ($[M - Cl]^+$) 164.1434, found 164.1427.

***N,N*-Diethyl-*α*-methylbenzylamine (3bb•HCl, Figure 3)**

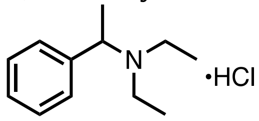

Prepared according to general procedure **C** using *α*-methylbenzylamine (**1b,** 121.7 mg, 1.00 mmol) for 5 h. The product was treated with HCl in diethyl ether to afford **3bb**•HCl as a yellow oil (189 mg, 89% yield). Analytical data for **3bb**•HCl: ^{1}H NMR (500 MHz, $CDCl_3$) δ 1.31 (t, *J* = 7.4 Hz, 3H), 1.47 (t, *J* = 7.2 Hz, 3H), 1.88 (d, *J* = 6.9 Hz, 3H), 2.72–2.80 (m, 1H), 3.11–3.17 (m, 1H), 3.22–3.30 (m, 1H), 3.36–3.44 (m, 1H), 4.25 (quint, *J* = 6.9 Hz, 1H), 7.39–7.46 (m, 3H), 7.73 (d, *J* = 7.5 Hz, 2H), 11.91 (s, 1H); $^{13}C\{^1H\}$ NMR (125 MHz, $CDCl_3$) δ 8.3, 8.9, 18.3, 43.8, 44.0, 63.1, 128.8, 129.6, 129.7, 135.4; HRMS (ESI) calcd for $C_{12}H_{19}NH^+$ ($[M - Cl]^+$) 178.1590, found 178.1601.

***N,N*-Dipropyl-*α*-methylbenzylamine (3bc, Figure 3)**

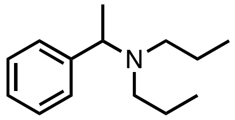

Prepared according to general procedure **C** using *α*-methylbenzylamine (**1b,** 121.5 mg, 1.00 mmol) for 18 h. The product was purified by column chromatography on silica gel (dichloromethane) to afford **3bc** as a light-yellow oil (171 mg, 83% yield). Analytical data for **3bc**: ^{1}H NMR (500 MHz, $CDCl_3$) δ 0.82 (t, *J* = 7.5 Hz, 6H), 1.32 (d, *J* = 6.9 Hz, 3H), 1.38–1.49 (m, 4H), 2.27–2.36 (m, 2H), 2.37–2.47 (m, 2H), 3.83 (q, *J* = 6.7 Hz, 1H), 7.18–7.23 (m, 1H), 7.29 (t, *J* = 7.5 Hz, 3H), 7.36 (d, *J* = 6.9 Hz, 2H); $^{13}C\{^1H\}$ NMR (125 MHz, $CDCl_3$) δ 12.0, 16.6, 21.1, 52.1, 59.1, 126.4, 127.8, 128.0, 145.1; HRMS (ESI) calcd for $C_{14}H_{23}NH^+$ ($[M + H]^+$) 206.1903, found 206.1904.

N-Ethyl-1-undecanamine hydrochloride (2cb•HCl, Figure 3)

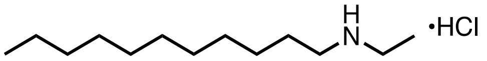

Prepared according to general procedure **A** using 1-undecanamine (**1c**, 34.5 mg, 0.20 mmol) for 50 min. **2cb**•HCl was obtained as a white powder (39 mg, 82% yield). Analytical data for **2cb**•HCl: Mp 206.8–208.7 °C; ^{1}H NMR (500 MHz, $CDCl_3$) δ 0.88 (t, J = 7.2 Hz, 3H), 1.20–1.41 (m, 16H), 1.48 (t, J = 7.2 Hz, 3H), 1.90 (quint, J = 7.7 Hz, 2H), 2.90 (d, J = 4.1 Hz, 2H), 3.03 (q, J = 5.5 Hz, 2H), 9.51 (s, 2H); ^{13}C{^{1}H} NMR (125 MHz, $CDCl_3$) δ 11.3, 14.2, 22.8, 26.1, 27.0, 29.2, 29.4, 29.5, 29.6, 32.0, 42.8, 47.4; HRMS (ESI) calcd for $C_{13}H_{29}NH^+$ ([M – Cl]$^+$) 200.2373, found 200.2375.

N-Propyl-1-undecanamine hydrochloride (2cc•HCl, Figure 3)

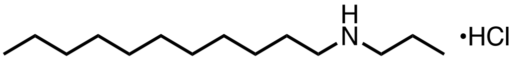

Prepared according to general procedure **A** using 1-undecanamine (**1c**, 34.7 mg, 0.20 mmol) for 55 min. **2cc**•HCl was obtained as a yellow powder (48 mg, 96% yield). Analytical data for **2cc**•HCl: Mp 239.0–241.7 °C; ^{1}H NMR (500 MHz, $CDCl_3$) δ 0.88 (t, J = 7.2 Hz, 3H), 1.01 (t, J = 7.5 Hz, 3H), 1.25–1.43 (m, 16H), 1.87–1.98 (m, 4H), 2.82–2.96 (m, 4H), 9.48 (s, 2H); ^{13}C{^{1}H} NMR (125 MHz, $CDCl_3$) δ 11.4, 14.2, 19.5, 22.8, 26.0, 27.0, 29.2, 29.4, 29.5, 29.6, 47.9, 49.4; HRMS (ESI) calcd for $C_{14}H_{31}NH^+$ ([M – Cl]$^+$) 214.2529, found 214.2531.

N,N-Dimethyl-1-undecanamine hydrochloride (3ca•HCl, Figure 3)

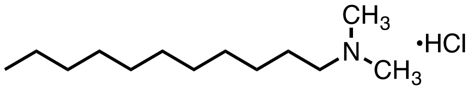

Prepared according to general procedure **C** using 1-undecanamine (**1c**, 171 mg, 1.0 mmol) for 3 h. The product was treated with HCl in diethyl ether to afford **3ca**•HCl as a white powder (211 mg, 89% yield). Analytical data for **3ca**•HCl: Mp 193.3–194.6 °C; ^{1}H NMR (500 MHz, $CDCl_3$) δ 0.88 (t, J = 6.5 Hz, 3H), 1.10–1.40 (m, 16H), 1.82–1.88 (m, 2H), 2.81 (d, J = 4.2 Hz, 6H), 2.96–3.01 (m, 2H), 12.36 (bs, 1H); ^{13}C{^{1}H} NMR (125 MHz, $CDCl_3$) δ 14.2, 22.7, 24.3, 26.7, 29.1, 29.4(2C), 29.5(2C), 31.9, 42.8, 58.1; HRMS (ESI) calcd for $C_{13}H_{29}NH^+$ ([M – Cl]$^+$) 200.2373, found 200.2317.

N,N-Diethyl-1-undecanamine (3cb, Figure 3)

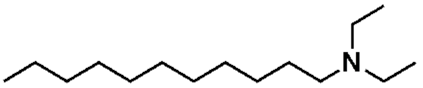

Prepared according to general procedure **C** using 1-undecanamine (**1c**, 171 mg, 1.0 mmol) for 4 h to afford **3cb** as a light-yellow oil (183 mg, 81% yield). Analytical data for **3cb**: ^{1}H NMR (500 MHz, $CDCl_3$) δ 0.86 (t, J = 6.9 Hz, 3H), 1.00 (t, J = 7.2 Hz, 6H), 1.10–1.35 (m, 16H), 1.38–1.48 (m, 2H), 2.38 (t, J = 8.0 Hz, 2H), 2.50 (q, J = 7.3

Hz, 4H); $^{13}C\{^1H\}$ NMR (125 MHz, $CDCl_3$) δ 11.8, 14.2, 22.8, 27.1, 27.8, 29.4, 29.7(4C), 32.0, 47.0, 53.1; HRMS (ESI) calcd for $C_{15}H_{33}NH^+$ ($[M + H]^+$) 228.2686, found 228.2630.

***N,N*-Dipropyl-1-undecanamine (3cc, Figure 3)**

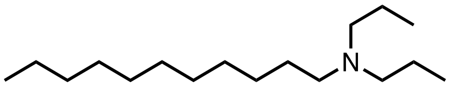

Prepared according to general procedure **C** using 1-undecanamine (**1c**, 171 mg, 1.0 mmol) for 8 h. The product was purified by column chromatography on silica gel (dichloromethane/methanol 8:1) to afford **3cc** as a yellow oil (187 mg, 73% yield). Analytical data for **3cc**: ^1H NMR (500 MHz, $CDCl_3$) δ 0.84–0.91 (m, 9H), 1.20–1.32 (m, 16H), 1.41–1.48 (m, 6H), 2.34–2.40 (m, 6H); $^{13}C\{^1H\}$ NMR (125 MHz, $CDCl_3$) δ 12.1, 14.2, 20.3, 22.8, 27.1, 27.8, 29.4, 29.7(4C), 32.0, 54.4, 56.4; HRMS (ESI) calcd for $C_{17}H_{37}NH^+$ ($[M + H]^+$) 256.2999, found 256.2956.

***N,N*-Dimethyl-1-naphthylmethylamine (3da, Figure 3)**

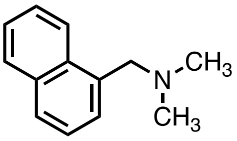

Prepared according to general procedure **C** using 1-naphthylmethylamine (**1d**, 157 mg, 1.0 mmol) for 2.5 h to afford **3da** as a light-yellow oil (157 mg, 85% yield). Analytical data for **3da**: ^1H NMR (600 MHz, $CDCl_3$) δ 2.28 (s, 6H), 3.80 (s, 2H), 7.36–7.41 (m, 2H), 7.45 (t, J = 7.6 Hz, 1H), 7.51 (t, J = 7.2 Hz, 1H), 7.74–7.78 (m, 1H), 7.83 (d, J = 7.6 Hz, 1H), 8.25 (d, J = 8.3 Hz, 1H); $^{13}C\{^1H\}$ NMR (150 MHz, $CDCl_3$) δ 45.8, 62.7, 124.6, 125.2, 125.7, 126.1, 127.5, 128.1, 128.5, 132.6, 134.0, 134.9; HRMS (ESI) calcd for $C_{13}H_{15}NH^+$ ($[M + H]^+$) 186.1277, found 186.1291. These data are consistent with the literature values. [11]

4-Chloro-*N,N*-dimethylbenzylamine hydrochloride (3ea•HCl, Figure 3)

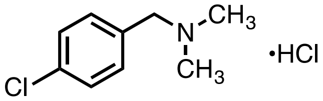

Prepared according to general procedure **C** using 4-chloro-benzylamine (**1e**, 142 mg, 1.0 mmol) for 3 h. The product was treated with HCl in diethyl ether to afford **3ea** •HCl as a light-yellow powder (186 mg, 90% yield). Analytical data for **3ea** •HCl: Mp 204.6–206.2 °C (lit. [12] 212–213 °C); ^1H NMR (500 MHz, $CDCl_3$) δ 2.80 (s, 6H), 4.22 (s, 2H), 7.43 (d, J = 8.3 Hz, 2H), 7.64 (d, J = 8.3 Hz, 2H), 12.67 (bs, 1H); $^{13}C\{^1H\}$ NMR (125 MHz, $CDCl_3$) δ 42.4, 60.6, 127.1, 129.8, 132.6, 136.7; HRMS (ESI) calcd for $C_9H_{13}ClNH^+$ ($[M - Cl]^+$) 170.0731, found 170.0739; calcd for $C_7H_6Cl^+$ ([M –

$NH(CH_3)_2Cl]^+$) 125.0153, found 125.0152. These data are consistent with the literature values.[13]

_N_-[2-(3,4-Dimethoxyphenyl)ethyl]dimethylamine (3fa, Figure 3)

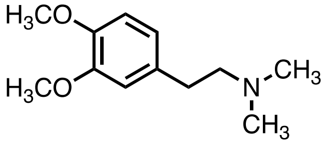

Prepared according to general procedure **C** using 3,4-dimethoxyphenethylamine (**1f**, 181 mg, 1.0 mmol) for 4 h. After the photocatalyst was removed by filtration, the filtrate was concentrated to give **3fa** as a yellow oil (193 mg, 92% yield). Analytical data for **8**: ^{1}H NMR (500 MHz, $CDCl_3$) δ 2.29 (s, 6H), 2.51 (t, J = 8.0 Hz, 2H), 2.73 (t, J = 8.0 Hz, 2H), 3.85 (s, 3H), 3.87 (s, 3H), 6.71–6.76 (m, 2H), 6.79 (d, J = 8.0 Hz, 1H); $^{13}C\{^1H\}$ NMR (125 MHz, $CDCl_3$) δ 34.2, 45.6, 56.0 (d), 61.9, 111.4, 112.0, 120.5, 133.1, 147.4, 149.0; HRMS (ESI) calcd for $C_{12}H_{19}NO_2H^+$ ([M + H]$^+$) 210.1489, found 210.1498. These data are consistent with the literature values. [14]

1-Boc-3-(dimethylamino)pyrrolidine (3ga, Figure 3)

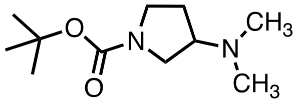

Prepared according to general procedure **C** using 3-amino-1-(*tert*-butoxycarbonyl)pyrrolidine (**1g**, 186 mg, 1.0 mmol) for 5 h. The product was purified by column chromatography on NH silica gel (hexane/ethyl acetate 3:1) to afford **3ga** as a colorless oil (189 mg, 88% yield). Analytical data for **3ga**: ^{1}H NMR (500 MHz, $CDCl_3$) δ 1.46 (s, 9H), 1.67–1.80 (m, 1H), 2.00–2.10 (m, 1H), 2.25 (s, 6H), 2.59–2.69 (m, 1H), 3.01–3.10 (m, 1H), 3.26–3.31 (m, 1H), 3.46–3.68 (m, 2H); $^{13}C\{^1H\}$ NMR (125 MHz, $CDCl_3$) δ 28.6, 29.8, 30.7, 44.4, 44.8, 45.2, 50.1, 50.4, 64.9, 65.7, 79.2(2C), 154.5; HRMS (ESI) calcd for $C_{11}H_{22}N_2O_2Na^+$ ([M + Na]$^+$) 237.1573, found 237.1589; calcd for $C_{22}H_{44}N_4O_4Na^+$ ([2M + Na]$^+$) 451.3255, found 451.3258.

_N_-_α_-Acetyl-_N_-dimethyl-lysine methyl ester (3ha, Figure 3)

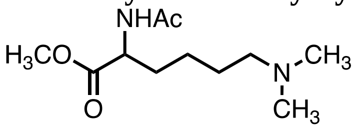

Prepared according to general procedure **C** using *N*-*α*-acetyl-lysine methyl ester hydrochloride (**1h,** 24.0 mg, 0.10 mmol), Au (5 wt %)/TiO_2 (10 mg) and Cu (5 wt %)/TiO_2 (10 mg) for 10 h. After separation of the catalyst, the mixture was treated with *N*,*N*-diethylmethylamine (87 mg, 1 mmol) and stirred for 15 min. The product was purified by successive column chromatography on NH silica gel (ethyl acetate) and silica gel (methanol) to afford **3ha** as a colorless oil (20 mg, 87% yield). Analytical data

for **3ha**: ^{1}H NMR (500 MHz, CDCl$_3$) δ 1.26–1.40 (m, 2H), 1.43–1.54 (m, 2H), 1.65–1.73 (m, 1H), 1.81–1.89 (m, 1H), 2.02 (s, 3H), 2.21 (s, 6H), 2.26 (t, *J* = 7.5 Hz, 2H), 3.74 (s, 3H), 4.59 (q, *J* = 6.9 Hz, 1H), 6.23 (d, *J* = 7.5 Hz, 1H); ^{13}C{^{1}H} NMR (125 MHz, CDCl$_3$) δ 23.0, 23.2, 27.1, 32.2, 45.4, 52.2, 52.4, 59.3, 170.0, 173.2; HRMS (ESI) calcd for $C_{11}H_{22}N_2O_3H^+$ ([M + H]$^+$) 231.1703, found 231.1705.

4-Methoxyphenyl 2-*N*,*N*-dimethyl-amino-3,6-di-*O*-benzyl-2-deoxy-*β*-D-glucopyranoside (3ia, Figure 3)

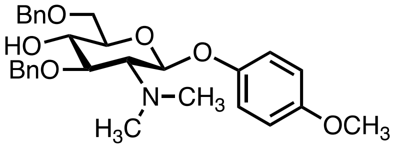

Prepared according to general procedure **C** using 4-methoxyphenyl 2-amino-3,6-di-*O*-benzyl-2-deoxy-*β*-D-glucopyranoside (**1i,** 233.2 mg, 0.50 mmol) at 50 °C for 12 h. The product was purified by column chromatography on silica gel (hexane/ethyl acetate 5:1) to afford **3ia** as a colorless oil (186 mg, 75% yield). Analytical data for **3ia**: ^{1}H NMR (500 MHz, CDCl$_3$) δ 2.55 (s, 6H), 2.65 (bs, 1H), 2.89 (t, *J* = 9.2 Hz, 1H), 3.44–3.52 (m, 2H), 3.60 (t, *J* = 9.2 Hz, 1H), 3.66 (q, *J* = 5.5 Hz, 1H), 3.74 (s, 3H), 3.82 (dd, *J* = 3.5 Hz, 1H), 4.55 (s, 2H), 4.73 (d, *J* = 11.5 Hz, 1H), 4.95 (d, *J* = 8.0 Hz, 1H), 5.02 (d, *J* = 11.5 Hz, 1H), 6.78 (d, *J* =9.2 Hz, 2H), 7.03 (d, *J* = 9.2 Hz, 2H), 7.24–7.31 (m, 6H), 7.34 (t, *J* = 7.5 Hz, 2H), 7.39 (d, *J* = 6.9 Hz, 2H); ^{13}C{^{1}H} NMR (125 MHz, CDCl$_3$) δ 42.0, 55.7, 68.6, 70.5, 71.8, 73.7, 74.6 (d), 80.2, 100.5, 114.6, 118.2, 127.7, 127.9, 128.2, 128.3, 128.5, 128.7, 138.3, 139.1, 151.4, 155.2; HRMS (ESI) calcd for $C_{29}H_{35}NO_6Na^+$ ([M + Na]$^+$) 516.2357, found 516.2359.

Alverine (5, Figure 3)

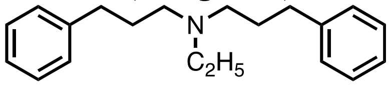

Prepared according to general procedure **A** using bis(3-phenylpropyl)amine (**4**, 253.9 mg, 1.00 mmol) for 3.5 h. The product was purified by column chromatography on silica gel (hexane/ethyl acetate 2:1) to afford **5** as a colorless oil (254 mg, 90% yield). Analytical data for **5**: ^{1}H NMR (500 MHz, CDCl$_3$) δ 0.98 (t, *J* = 7.2 Hz, 3H), 1.75 (quint, *J* = 7.6 Hz, 4H), 2.45 (t, *J* = 7.7 Hz, 4H), 2.51 (q, *J* = 7.1 Hz, 2H), 2.60 (t, *J* = 7.7 Hz, 4H), 7.17 (d, *J* = 7.5 Hz, 6H), 7.26 (t, *J* = 7.7 Hz, 4H); ^{13}C{^{1}H} NMR (125 MHz, CDCl$_3$) δ 11.9, 29.0, 33.9, 47.6, 53.1, 125.8, 128.4, 128.5, 142.6; HRMS (ESI) calcd for $C_{20}H_{27}NH^+$ ([M + H]$^+$) 282.2216, found 282.2229. These data are consistent with the literature values. [15]

Me-Desloratadine (7, Figure 3)

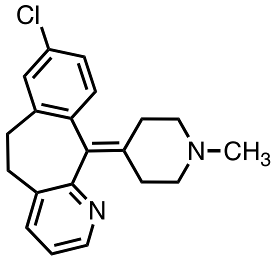

Prepared according to general procedure **C** using desloratadine (**6**, 155.9 mg, 0.50 mmol) at 50 °C for 17 h. The product was purified by column chromatography on NH silica gel (hexane/ethyl acetate 3:1) to afford **7** as a white solid (143 mg, 88% yield). Analytical data for **26**: Mp 114.4–116.5 °C (lit.[16] 116–119 °C); ^{1}H NMR (500 MHz, $CDCl_3$) δ 2.04–2.10 (m, 2H), 2.27 (s, 3H), 2.33–2.47 (m, 3H), 2.52–2.58 (m, 1H), 2.69–2.74 (m, 2H), 2.76–2.86 (m, 2H), 3.34–3.45 (m, 2H), 7.07 (q, *J* = 4.2 Hz, 1H), 7.11–7.16 (m, 3H), 7.42 (d, *J* = 7.5 Hz, 1H), 8.40 (d, *J* = 4.6 Hz, 1H); $^{13}C\{^{1}H\}$ NMR (125 MHz, $CDCl_3$) δ 30.9, 31.1, 31.5, 31.9, 46.1, 57.0, 122.2, 126.1, 129.0, 130.9, 132.7, 132.9, 133.5, 137.3, 138.0, 138.4, 139.6, 146.7, 157.7; HRMS (ESI) calcd for $C_{20}H_{21}N_2ClNa^+$ ($[M + Na]^+$) 347.1285, found 347.1290. These data are consistent with the literature values.[16]

Table S6. Comparing reactivity of the Cu–Au system with that of Cu/TiO_2 or Au/TiO_2.

entry	RNH_2, photocatalyst[a]	Product, yield[b] (time)
	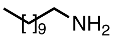	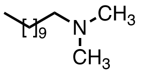
	**1c**	**3ca**
1	Au + Cu	63% (2 h) [89% (3 h)]
2	Au only	7% (2 h)
3	Cu only	39% (2 h)
	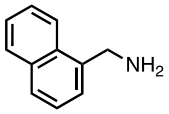	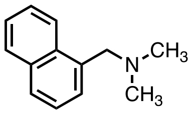
	**1d**	**3da**
4	Au + Cu	92% (2.5 h) [85 (2.5 h)]
5	Au only	13 (2.5 h)
6	Cu only	69 (2.5 h)
	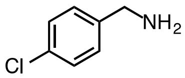	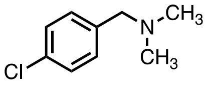
	**1e**	**3ea**
7	Au + Cu	59 (1.5 h) [90 (3 h)]
8	Au only	7 (1.5 h)
9	Cu only	43 (1.5 h)
	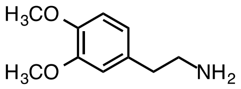	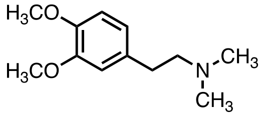
	**1f**	**3fa**
10	Au + Cu	96 (4 h) [92 (4 h)]
11	Au only	6 (4 h)
12	Cu only	65 (4 h)
	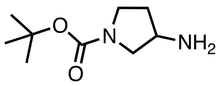	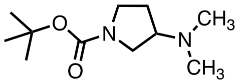
	**1g**	**3ga**
13	Au + Cu	68 (2 h) [88 (5 h)]
14	Au only	6 (2 h)
15	Cu only	44 (2 h)

[a]Conditions: Au + Cu: Au/TiO_2 22 mg, Cu/TiO_2 22 mg; Au only: Au/TiO_2 43 mg; Cu only: Cu/TiO_2 43 mg. RNH_2 (1.0 mmol), CH_3OH (250 equiv, 10 mL), 300 W Xe lamp with a UV-cold mirror (λ = 300–470 nm), Ar (1 atm), 25 °C, 1.5–5 h. [b]^{1}H NMR yield using dibenzyl ether (entries 1–3) or 2,2-dimethylpropan-1-ol (entries 4–15) as an internal standard. Isolated yield in brackets.

N-Methyl-N-[2-[2-(2-methoxyethoxy)ethoxy]ethyl]-α-methyl-benzenemethanamine (3bd, Figure 4)

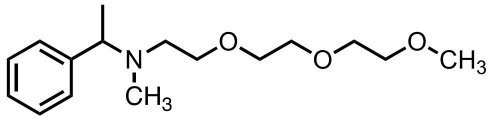

Prepared according to general procedure **D** using α-methylbenzylamine (**1b,** 121.7 mg, 1.00 mmol), and 2-[2-(2-methoxyethoxy)ethoxy]ethanol (329.8 mg, 2.01 mmol) at 50 °C for 13 h, then methanol (5.0 mL) for 6 h. The product was purified by column chromatography on NH silica gel (hexane/ethyl acetate 10:1) to afford **3bd** as a colorless oil (210 mg, 75% yield). Analytical data for **3bd**: ^{1}H NMR (500 MHz, $CDCl_3$) δ 1.36 (d, J = 6.3 Hz, 3H), 2.24 (s, 3H), 2.49 (quint, J = 6.4 Hz, 1H), 2.66 (quint, J = 6.5 Hz, 1H), 3.37 (s, 3H), 3.51–3.65 (m, 11H), 7.19–7.24 (m, 1H), 7.26–7.33 (m, 4H); ^{13}C{^{1}H} NMR (125 MHz, $CDCl_3$) δ 18.5, 39.5, 53.4, 59.1, 63.8, 69.9, 70.4, 70.7 (d), 72.0, 126.9, 127.8, 128.2, 143.8; HRMS (ESI) calcd for $C_{16}H_{27}NO_3H^+$ ($[M + H]^+$) 282.2064, found 282.2075.

8-Chloro-N-ethyl-N-(1-phenylethyl)octan-1-amine (3be, Figure 4)

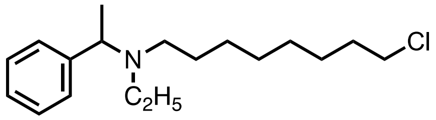

Prepared according to general procedure **D** using α-methylbenzylamine (**1b,** 121.4 mg, 1.00 mmol), and 8-chloro-1-octanol (330.0 mg, 2.00 mmol) for 16 h, then ethanol (5.0 mL) for 16 h. The product was purified by column chromatography on NH silica gel (hexane) and silica gel (ethyl acetate) to afford **3be** as a colorless oil (199 mg, 67% yield). Analytical data for **3be**: ^{1}H NMR (500 MHz, $CDCl_3$) δ 0.98 (t, J = 6.9 Hz, 3H), 1.17–1.30 (m, 6H), 1.32 (d, J = 6.9 Hz, 3H), 1.34–1.46 (m, 4H), 1.75 (quint, J = 7.2 Hz, 2H), 2.29–2.61 (m, 4H), 3.51 (t, J = 6.9 Hz, 2H), 3.80 (q, J = 6.7 Hz, 1H), 7.17–7.23 (m, 1H), 7.29 (t, J = 7.5 Hz, 2H), 7.35 (d, J = 7.5 Hz, 2H); ^{13}C{^{1}H} NMR (125 MHz, $CDCl_3$) δ 12.5, 17.5, 26.9, 27.4, 27.6, 29.0, 29.5, 32.7, 43.6, 45.2, 49.4, 59.2, 126.5, 127.7, 128.0, 145.2; HRMS (ESI) calcd for $C_{18}H_{30}NClH^+$ ($[M + H]^+$) 296.2140, found 296.2140.

3-[1-([$^2H_6$]Dimethylamino)ethyl]phenyl ethyl(methyl)carbamate (3aa-d_6, Figure 4)

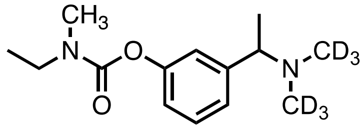

Prepared according to general procedure **C** using **1a** (1.00 g, 4.50 mmol), Au (5 wt %)/TiO_2 (100 mg), Cu (5 wt %)/TiO_2 (100 mg), and deuterated methanol (CD_3OD 10.0 mL, 250 mmol) for 16 h to afford **3aa**-d_6 as a light-yellow oil (1.05 g, 91% yield,

89%D). Analytical data for **3aa**-*d*$_6$ (observed as a 1:1 *cis*/*trans* isomeric mixture around the carbamate moiety): ^{1}H NMR (500 MHz, $CDCl_3$) δ 1.19 (t, J = 6.9 Hz, 1.5H), 1.24 (t, J = 6.9 Hz, 1.5H), 1.35 (d, J = 6.9 Hz, 3H), 2.15 (s, 0.67H), 2.98 (s, 1.5H), 3.06 (s, 1.5H), 3.24 (q, J = 6.7 Hz, 1H), 3.40 (q, J = 6.9 Hz, 1H), 3.47 (q, J = 6.9 Hz, 1H), 7.01 (d, J = 7.5 Hz, 1H), 7.06 (s, 1H), 7.11 (d, J = 8.0 Hz, 1H), 7.28 (t, J = 7.7 Hz, 1H); ^{13}C{^{1}H} NMR (125 MHz, $CDCl_3$) δ 12.6 (0.5C), 13.3 (0.5C), 20.2, 33.9 (0.5C), 34.3 (0.5C), 42.6 (sept, J_{CD} = 20.3 Hz), 44.1, 65.6, 120.3, 120.8, 124.3, 128.9, 145.8 (0.5C), 145.9 (0.5C), 151.6, 154.5 (0.5C), 154.7 (0.5C); HRMS (ESI) calcd for $C_{14}H_{16}D_6N_2O_2H^+$ ([M + H]$^+$) 257.2131, found 257.2111.

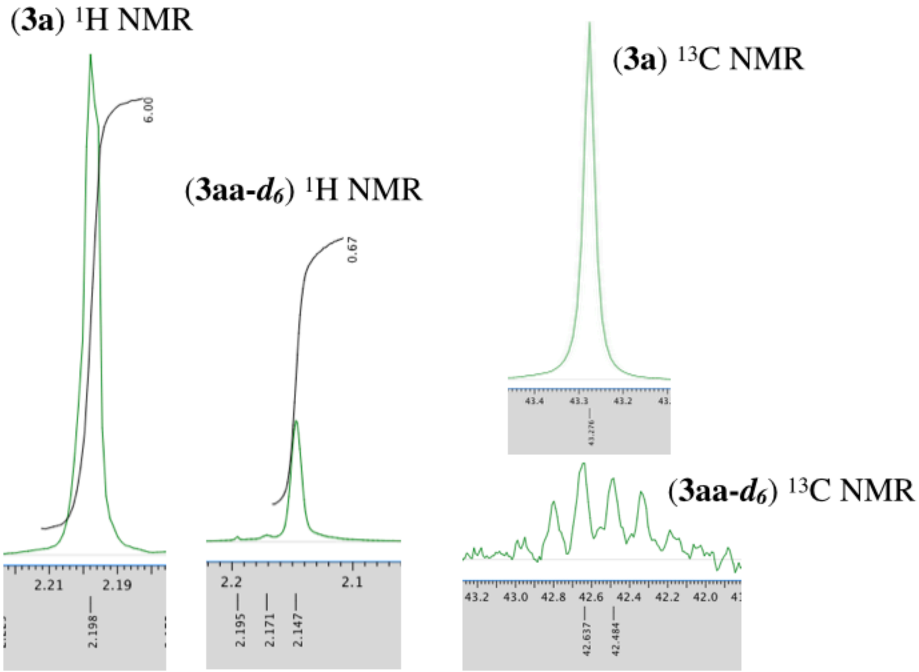

Venlafaxine-*d*$_6$ (3ja-*d*$_6$, Figure 4)

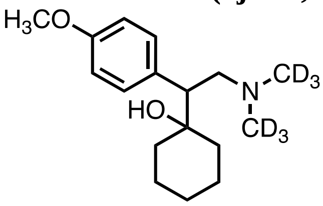

Prepared according to general procedure **C** using 1-[2-amino-1-(4-methoxyphenyl)ethyl]cyclohexanol (49.7 mg, 0.20 mmol), and deuterated methanol (CD_3OD 2.0 mL, 50 mmol) in a Young tube at 50 °C for 10 h. The product was purified by column chromatography on silica gel (ethyl acetate) to afford **3ja**-*d*$_6$ as a light-yellow oil (41 mg, 72% yield, > 95%D). Analytical data for **3ja**-*d*$_6$: ^{1}H NMR (500 MHz, $CDCl_3$) δ 0.82–1.01 (m, 2H), 1.25–1.41 (m, 2H), 1.47–1.60 (m, 3H), 1.61–1.79 (m, 3H), 2.28 (dd, J = 12.9 Hz, 1H), 2.94 (dd, J = 12.3 Hz, 1H), 3.27 (t, J = 12.6 Hz, 1H),

3.79 (s, 3H), 6.81 (d, J = 8.6 Hz, 2H), 7.05 (d, J = 9.2 Hz, 2H); $^{13}C\{^1H\}$ NMR (125 MHz, $CDCl_3$) δ 21.4, 21.7, 26.1, 31.3, 38.2, 44.7 (sept, J_{CD} = 20.8 Hz), 51.7, 55.3, 61.2, 74.3, 113.4, 130.2, 132.9, 158.4; HRMS (ESI) calcd for $C_{17}H_{21}D_6NO_2H^+$ ($[M + H]^+$) 284.2491, found 284.2491.

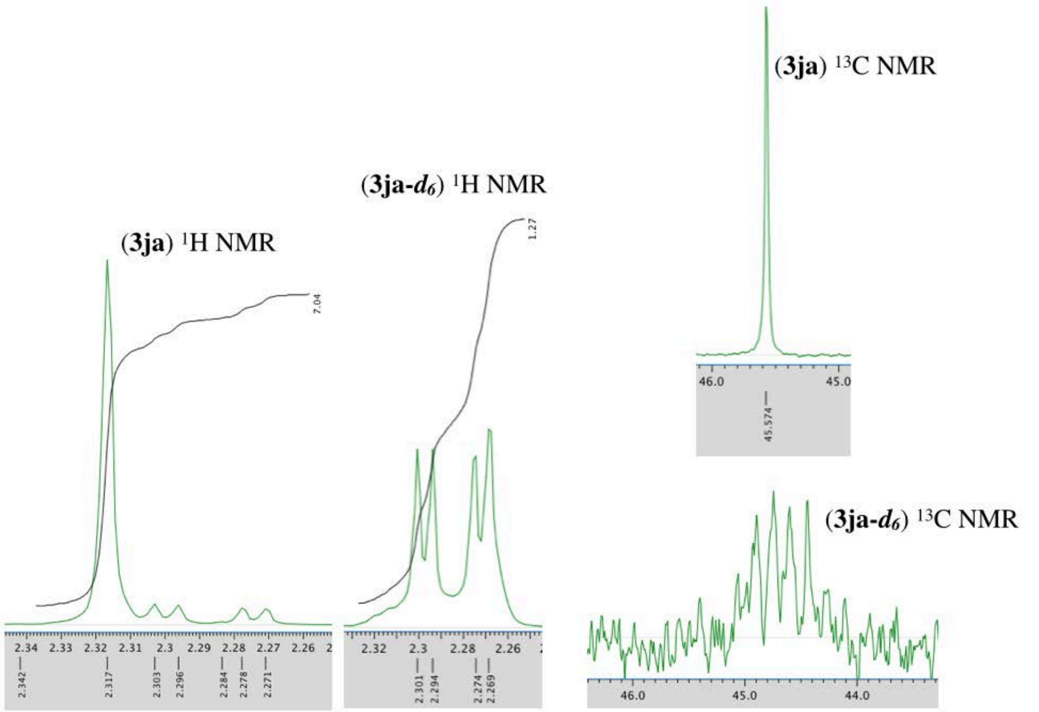

Imipramine-d_3 (8-d_3, Figure 4)

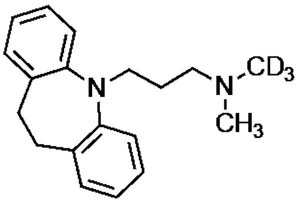

Prepared according to general procedure **C** using 10,11-dihydro-*N*-methyl-5*H*-dibenz[*b*,*f*]azepine-5-propanamine hydrochloride (1:1) (60.8 mg, 0.20 mmol), and deuterated methanol (CD_3OD, 2.0 mL, 50 mmol) in a Young tube at 25 °C for 5 h. After reaction mixture was treated with sat Na_2CO_3 aq to pH 10 and extracted by ethyl acetate to afford **8**-d_3 as a colorless oil (41 mg, 94% yield, > 95%D). Analytical data for **8**-d_3: ^1H NMR (500 MHz, $CDCl_3$) δ 1.71 (quint, J = 7.2 Hz, 2H), 2.13 (s, 3H), 2.29 (t, J = 7.2 Hz, 2H), 3.15 (s, 4H), 3.76 (t, J = 6.9 Hz, 2H), 6.86–6.93 (m, 2H), 7.05–7.13 (m, 6H); $^{13}C\{^1H\}$ NMR (125 MHz, $CDCl_3$) δ 26.3, 32.3, 44.9 (sept, J_{CD} = 19.4 Hz), 45.6, 49.0, 57.8, 120.1, 122.5, 126.4, 129.9, 134.3, 148.4; HRMS (ESI) calcd for $C_{19}H_{21}D_3N_2H^+$ ($[M + H]^+$) 284.2201, found 284.2191.

8. Photocatalyst recycling experiments

As per the representative procedure for N-methylation of **1a** to **3aa**, N-methylation of **1c** (171.6 mg, 1.00 mmol) with methanol (250 equiv, 10 mL), Au (5 wt %)/TiO_2 (25 mg), and Cu (5 wt %)/TiO_2 (25 mg) was conducted for 20 h (1st run). After the reaction mixture was transferred to a 50-mL centrifuge tube and centrifuged (3500 rpm, 10 min), the precipitate was collected by decantation, washed with methanol (10 mL), and collected by centrifugation and decantation. Drying the precipitate under vacuum (0.01 mm Hg) at rt overnight gave a used photocatalyst (43.8 mg). This photocatalyst was used for N-methylation of **1a** to **3aa** (2nd to 10th runs).

Table S7. Recycling experiments[a]

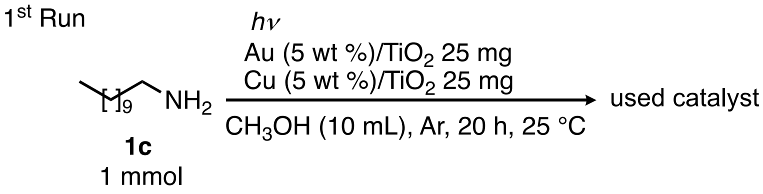

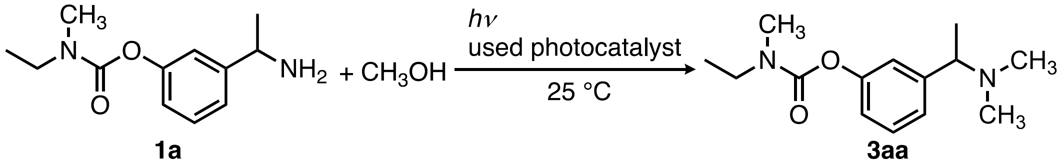

run	**1a** (mg)	**1a** (mmol)	photocatalyst (mg)	CH_3OH (mL)	yield of **3aa** (%)[b]
2nd	222.4	1.00	43.8	10.0	98
3rd	188.8	0.85	37.3[c]	8.5	97
4th	177.2	0.80	35.3	8.0	97
5th	170.8	0.77	34.0	7.7	96
6th	150.6	0.68	30.1	6.8	95
7th	142.2	0.64	28.0	6.4	92
8th	124.7	0.56	24.7	5.6	91
9th	102.6	0.46	20.1	5.0	90
10th	95.6	0.43	18.7	5.0	82[d]

[a]Conditions: 300 W Xe lamp with a UV-cold mirror (λ = 300–470 nm), Ar (1 atm), 25 °C, 4 h. [b]^{1}H NMR yields using 2,2-dimethylpropan-1-ol as internal standard. [c]Photocatalyst lost because some sample was taken out for GC and ^{1}H NMR analysis in second run. [d]Isolated yield.

Table S8. Activity of Pre-irradiated Photocatalyst[a]

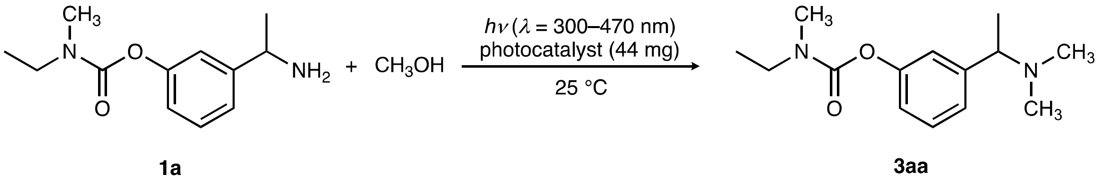

Entry	Photocatalyst	Yield of **3aa** [%]				
		30 min	1 h	2 h	3 h	4 h
1	Cu (5 wt %)/TiO_2 (22 mg) + Au (5 wt %)/TiO_2 (22 mg)	< 1	5	64	93	96
2	Cu–Au photocatalyst irradiated in methanol[b]	< 1	3	38	90	94
3	used Cu–Au photocatalyst[c]	< 1	0	33	84	95

[a]Conditions: **1a** (1.0 mmol), CH_3OH (10 mL, 250 mmol, 250 equiv), 300 W Xe lamp with a UV-cold mirror, Ar (1 atm), 25 °C. Yields of **3aa** were determined by ^1H NMR using 2,2-dimethylpropan-1-ol as an internal standard. [b]Preparation: a mixture of Cu (5 wt %)/TiO_2 (100 mg) and Au (5 wt %)/TiO_2 (100 mg) was irradiated in methanol (15 mL) at 25 °C for 20 h under Ar (1 atm) using 300 W Xe lamp with a UV-cold mirror (λ = 300–470 nm). [c]Analogous sample to the used photocatalyst (1 time) in Table S7.

References:

[1] V. R. Arava, L. Gorentla, P. K. Dubey, *Int. J. Org. Chem.* **2011**, *1*, 26–32.
[2] M. Fuchs, D. Koszelewski, K. Tauber, J. Sattler, W. Banko, A. K. Holzer, M. Pickl, W. Kroutil, K. Faber, *Tetrahedron* **2012**, *68*, 7691–7694.
[3] For a related patent, see: M. Watanabe, J. Hori, K. Murata, Eur. Pat. Appl. (2010), 30pp. EP2228377.
[4] C. Zha, G. B. Brown, W. J. Brouillette, *Bioorg. Med. Chem.* **2014**, *22*, 95–104.
[5] V. N. Tsarev, Y. Morioka, J. Caner, Q. Wang, R. Ushimaru, A. Kudo, H. Naka, S. Saito, *Org. Lett.* **2015**, *17*, 2530–2533.
[6] S. Shibata, R. Nagata, S. Saito, H. Naka, *Chem. Lett.* **2017**, *46*, 580–582.
[7] E. Zhang, C. Li, H. Liu, *Chin. J. Org. Chem.* **2013**, *33*, 1100–1103.
[8] K. Han, C. Kim, M-J. Kim, *J. Org. Chem.* **2010**, *75*, 3105–3108.
[9] C. S. Cooper, A. L. Peyton, R. J. Weinkam, *J. Org. Chem.* **1983**, *48*, 4116–4119.
[10] W. Meindl, *Arch. Pharm.* **1993**, *326*, 277–286.
[11] W. X. Chen, C. Y. Zhang, L. X. Shao, *Tetrahedron* **2014**, *70*, 880–885.
[12] R. Baltzly, P. B. Russell, *J. Am. Chem. Soc.* **1950**, *72*, 3410–3413.
[13] M. Barniol-Xicota, A. L. Turcu, S. Codony, C. Escolano, S. Vázquez, *Tetrahedron Lett.* **2014**, *55*, 2548–2550.
[14] M. H. S. A. Hamid, C. L. Allen, G. W. Lamb, A. C. Maxwell, H. C. Maytum, A. J. A. Waston, J. M. J. Williams, *J. Am. Chem. Soc.* **2009**, *131*, 1766–1774.
[15] S. P. Shan, X. Xiaoke, B. Gnanaprakasam, T. T. Dang, B. Ramalingam, H. V. Huynh, A. M. Seayad, *RSC Adv.* **2015**, *5*, 4434–4442.
[16] S. Cuboni, C. Devigny, B. Hoogeland, A. Strasser, S. Pomplun, B. Hauger, G. Höfner, K. T. Wanner, M. Eder, A. Buschauer, F. Holsboer, F. Hausch, *J. Med. Chem.* **2014**, *57*, 9473–9479.

7. NMR Charts

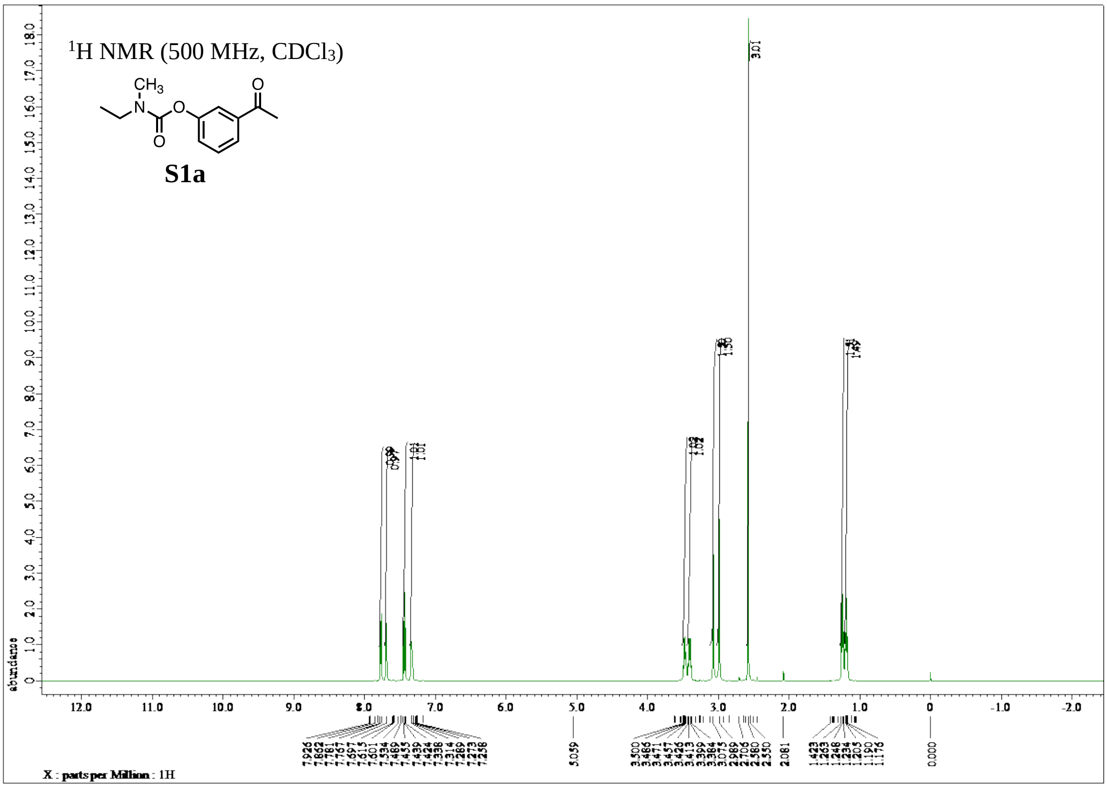
1H NMR (500 MHz, CDCl3)
S1a
X : parts per Million : 1H

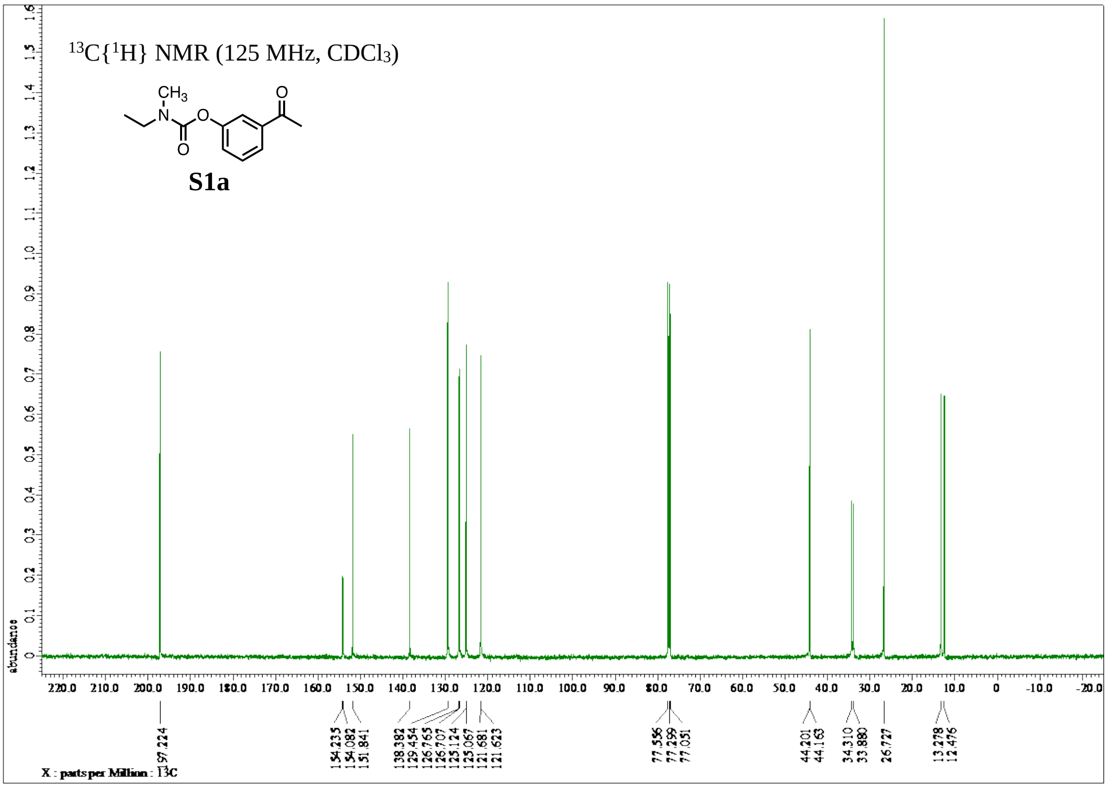
13C{1H} NMR (125 MHz, CDCl3)
S1a
X : parts per Million : 13C

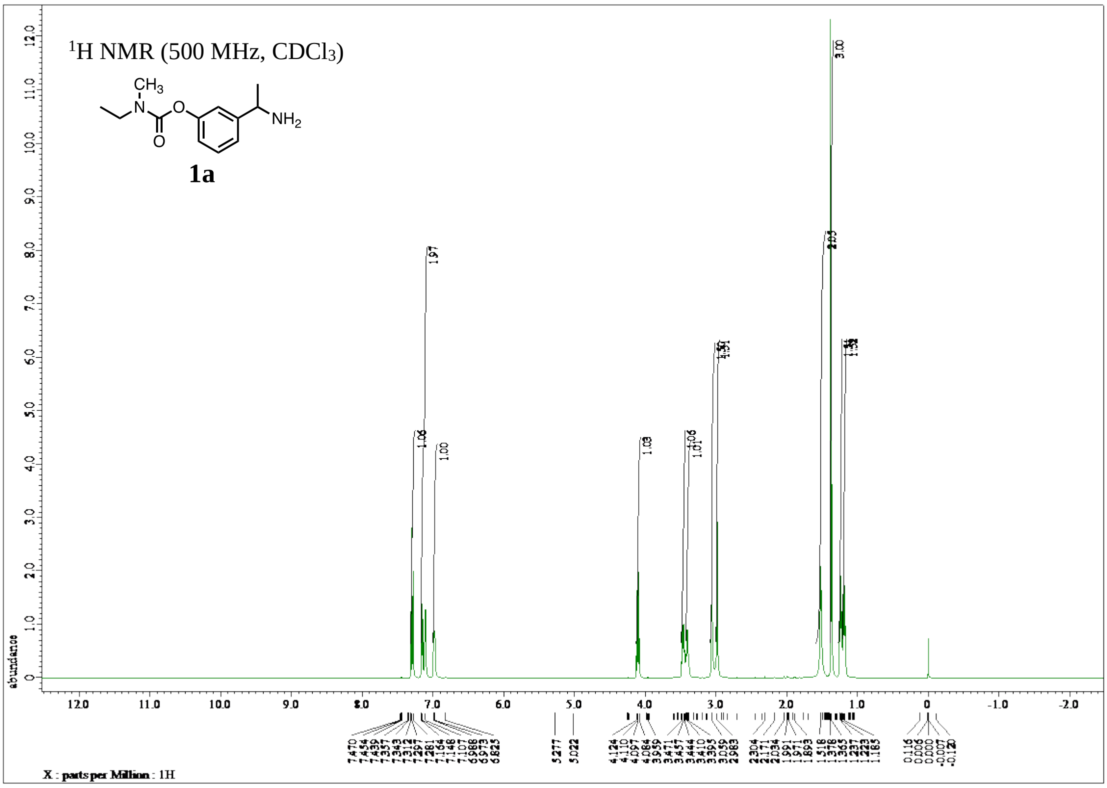
1H NMR (500 MHz, CDCl3)
CH3
N
O
O
NH2
1a
X : parts per Million : 1H
abundance

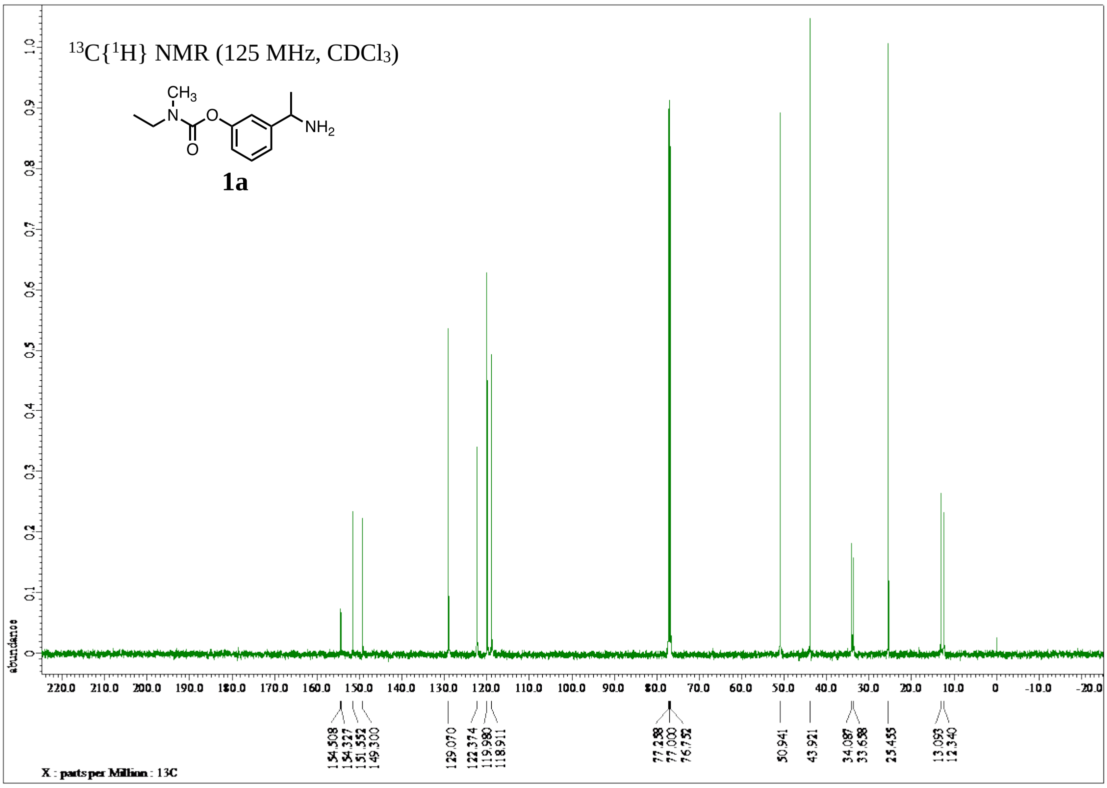
13C{1H} NMR (125 MHz, CDCl3)
CH3
N
O
O
NH2
1a
154.508
154.327
151.552
149.300
129.070
122.374
119.980
118.911
77.258
77.000
76.752
50.941
43.921
34.087
33.658
25.455
13.093
12.340
X : parts per Million : 13C
abundance

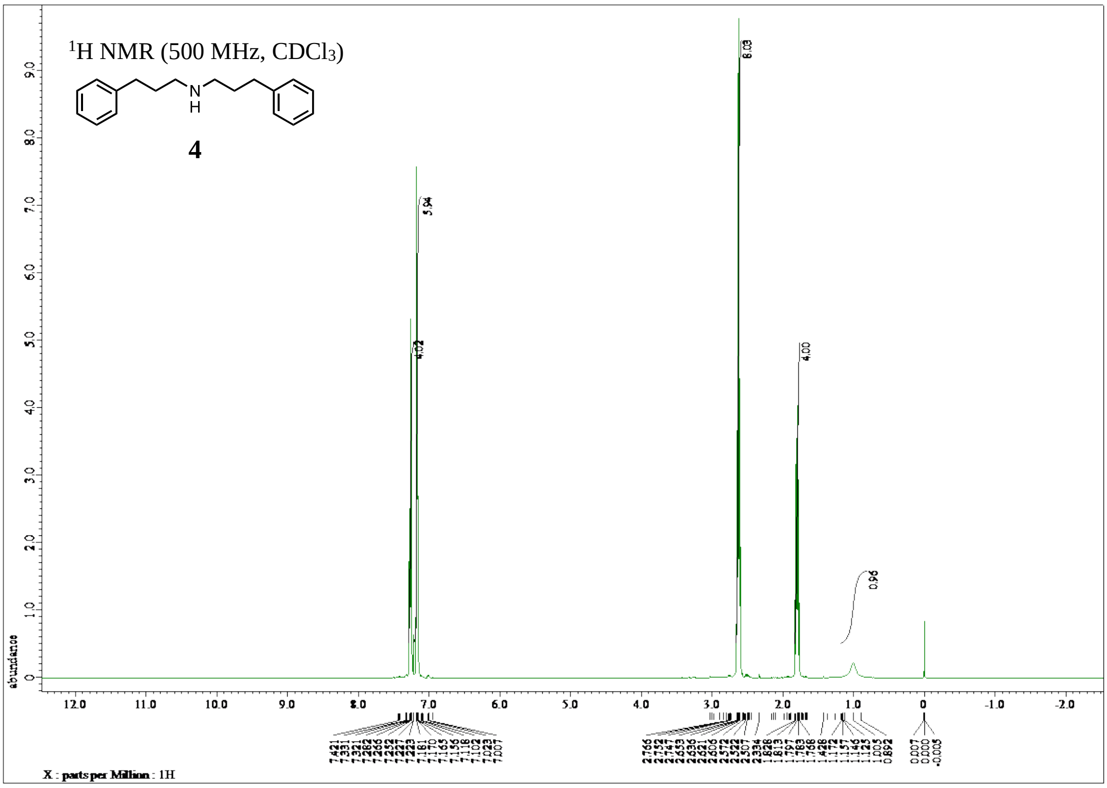

^{1}H NMR (500 MHz, CDCl$_3$)
N
H
4
X : parts per Million : 1H
abundance

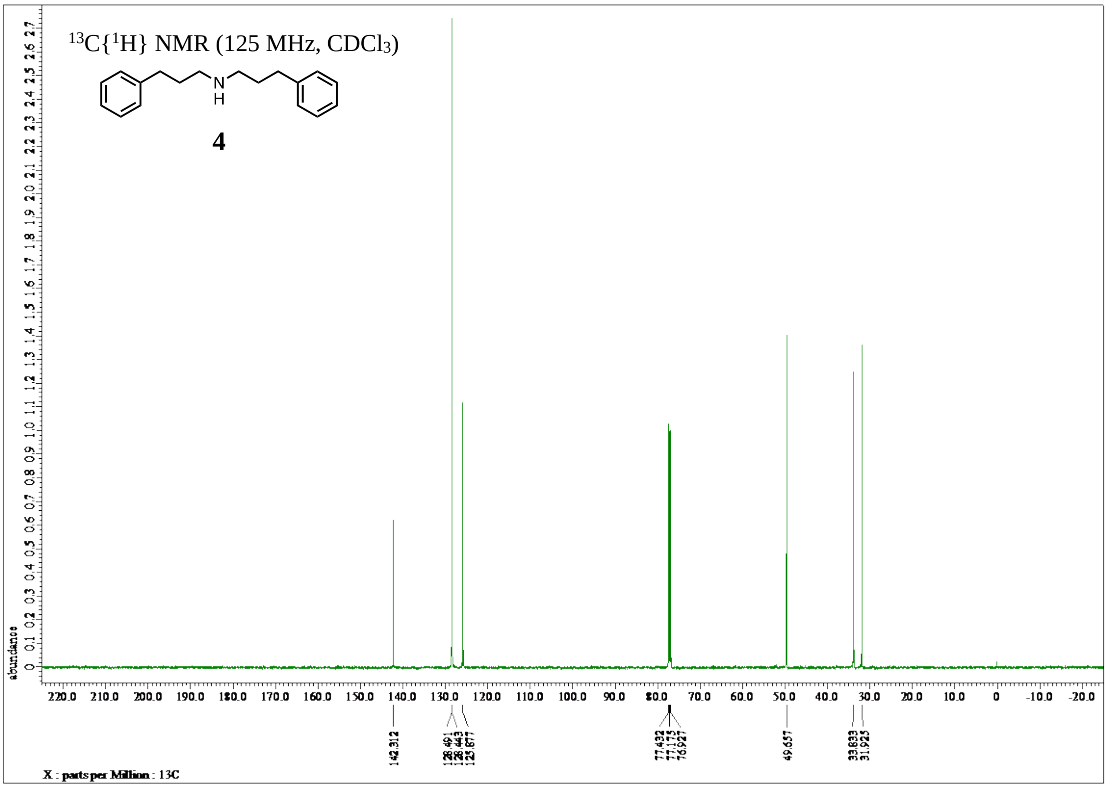

^{13}C{^{1}H} NMR (125 MHz, CDCl$_3$)
N
H
4
142.312
128.491
128.443
125.877
77.432
77.175
76.927
49.657
33.833
31.925
X : parts per Million : 13C
abundance

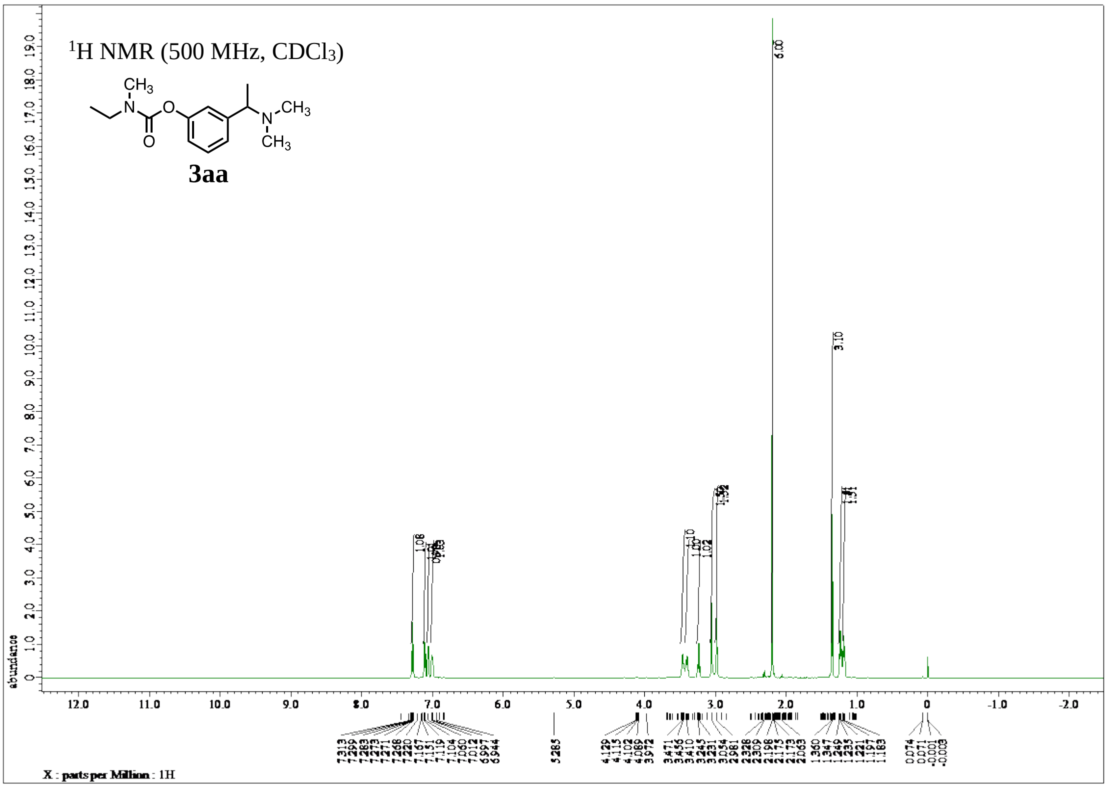

^{1}H NMR (500 MHz, CDCl$_3$)
3aa
X : parts per Million : 1H

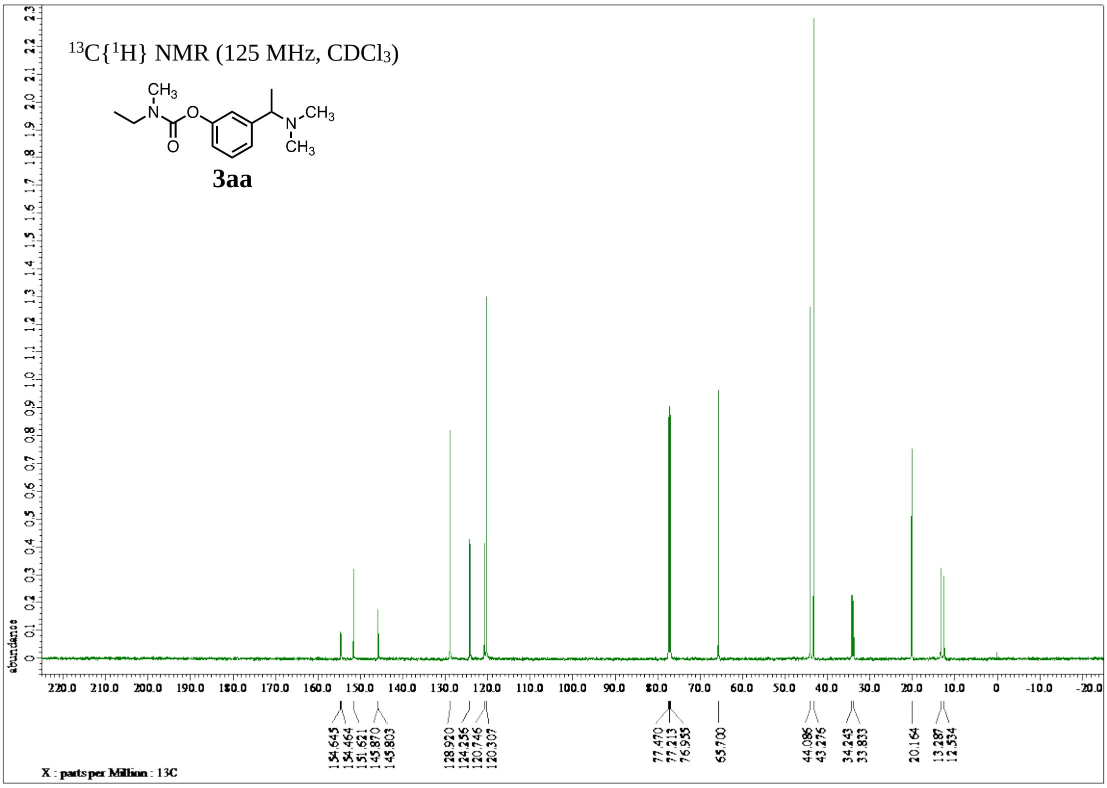

^{13}C{^{1}H} NMR (125 MHz, CDCl$_3$)
3aa
X : parts per Million : 13C

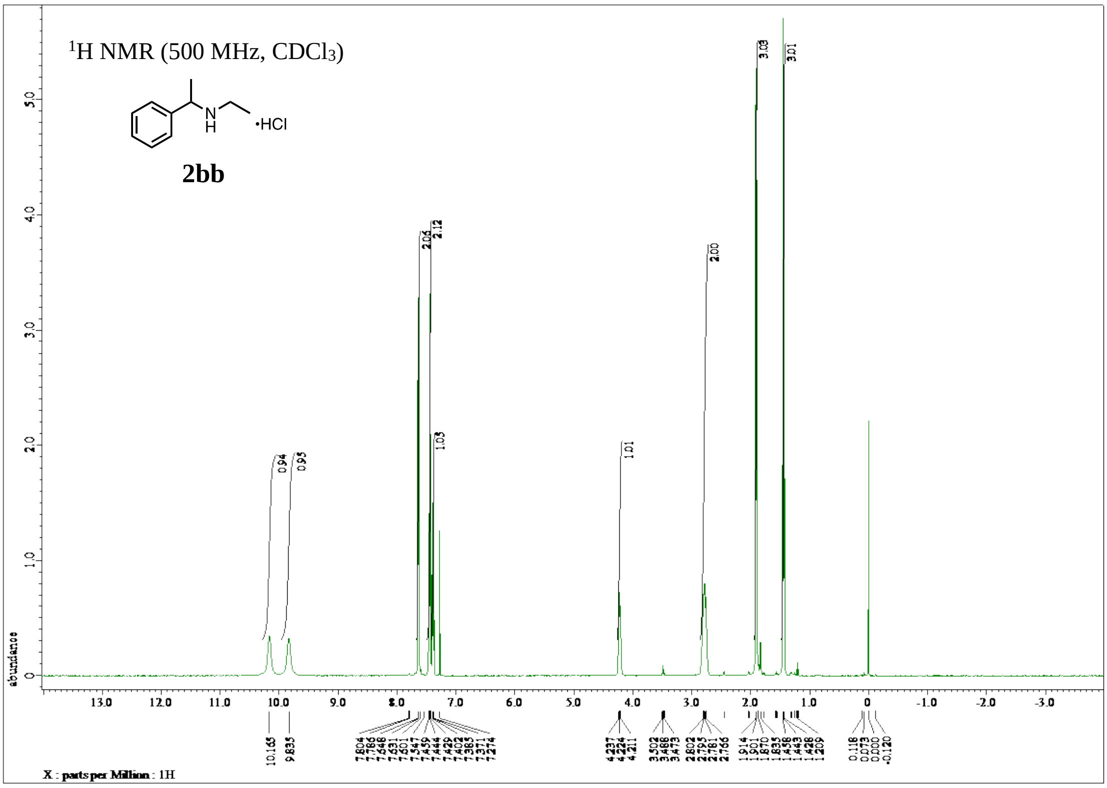

^{1}H NMR (500 MHz, CDCl$_3$)
•HCl
2bb
X : parts per Million : 1H
abundance

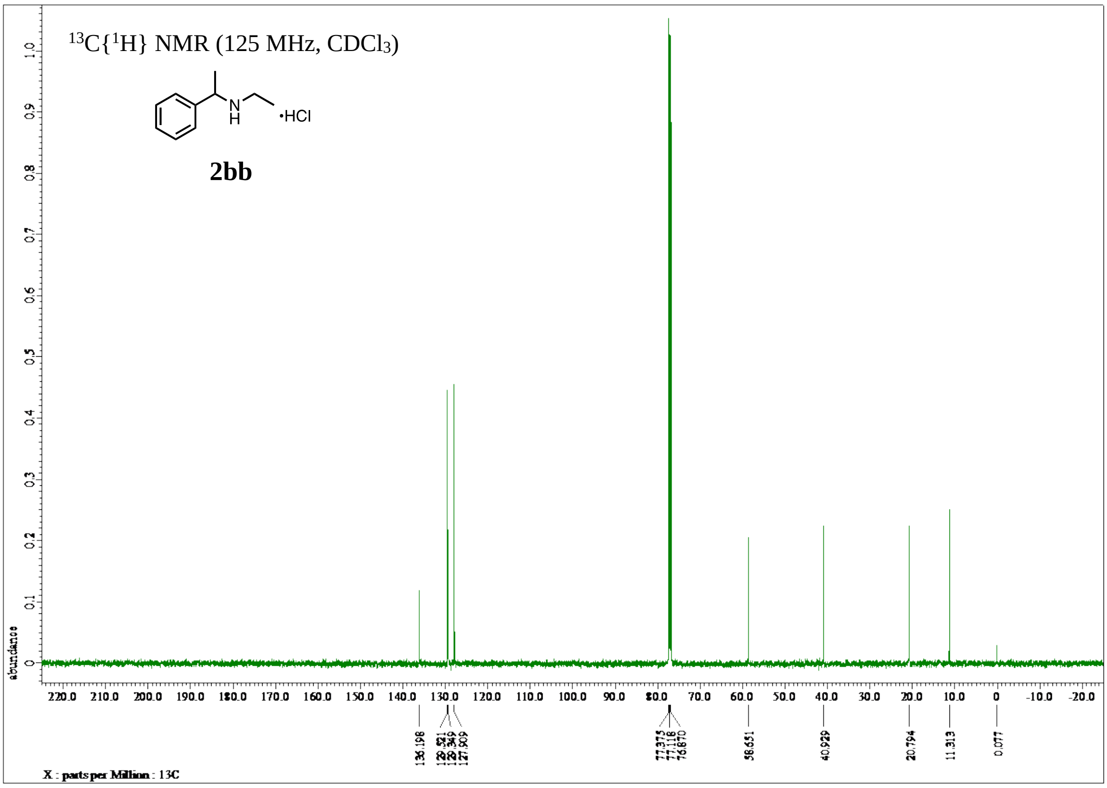

^{13}C{^{1}H} NMR (125 MHz, CDCl$_3$)
•HCl
2bb
X : parts per Million : 13C
abundance
136.198
77.375
77.118
76.870
58.651
40.929
20.794
11.313
0.077

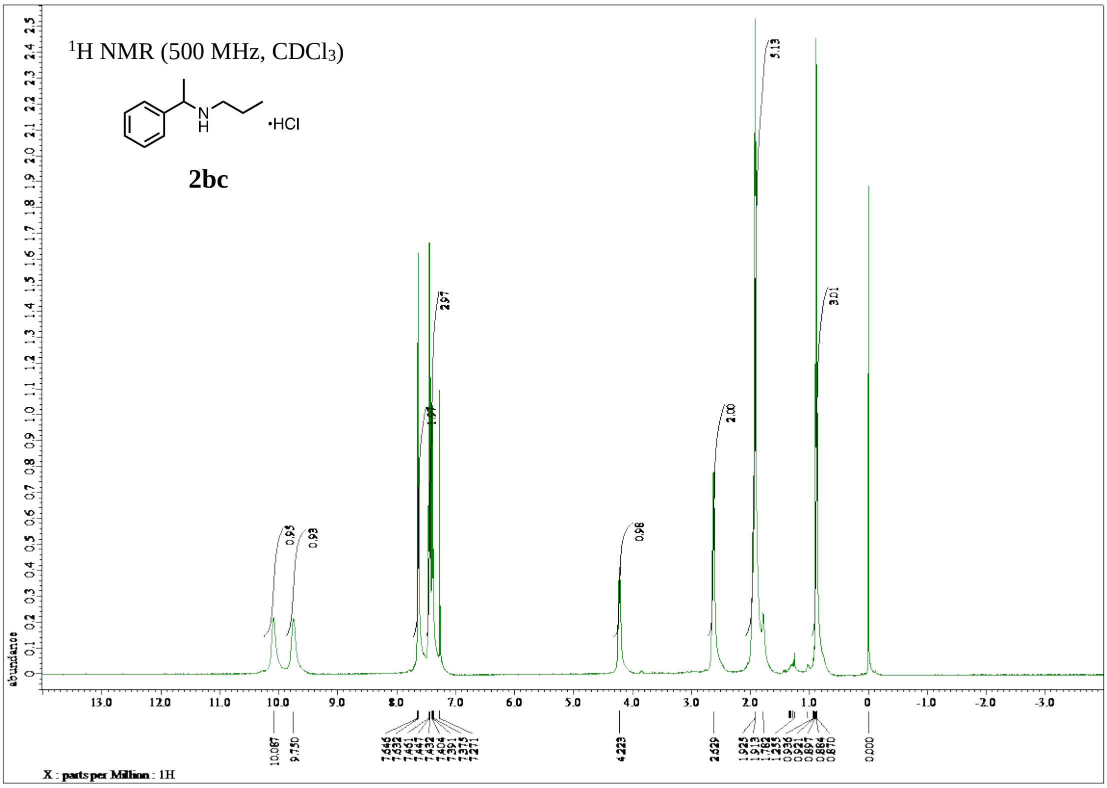

^{1}H NMR (500 MHz, CDCl$_3$)
•HCl
2bc
X : parts per Million : 1H

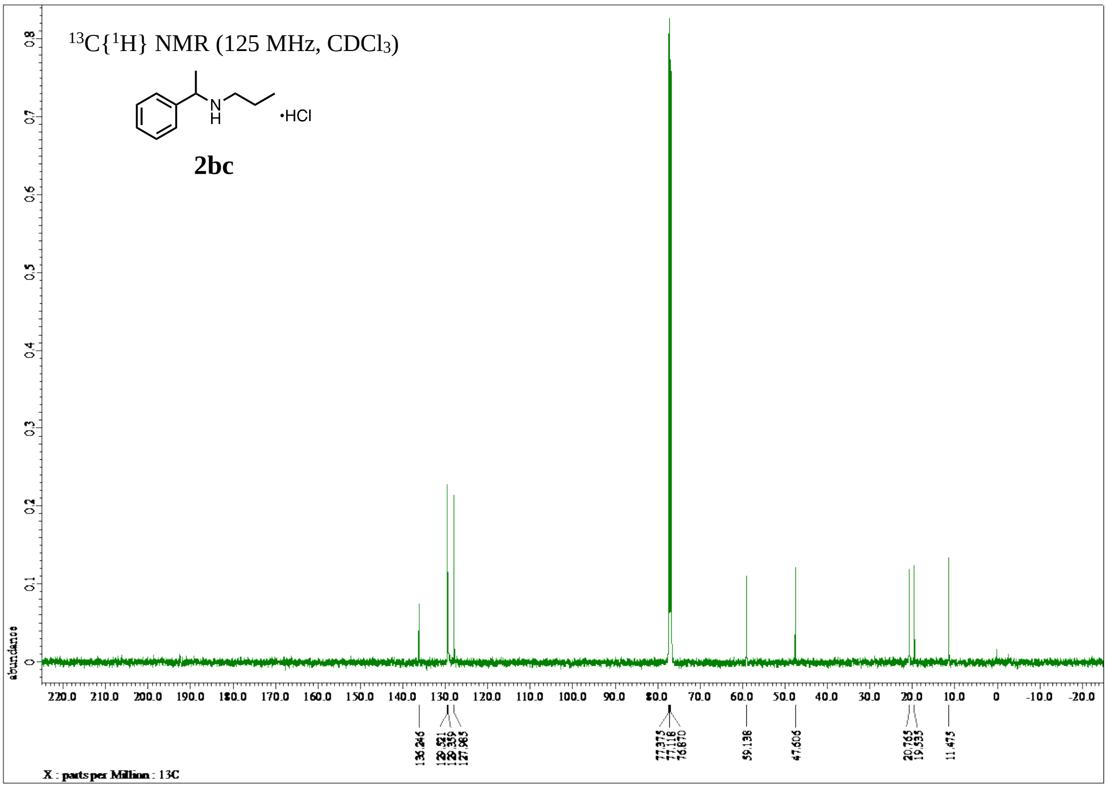

^{13}C{^{1}H} NMR (125 MHz, CDCl$_3$)
•HCl
2bc
X : parts per Million : 13C

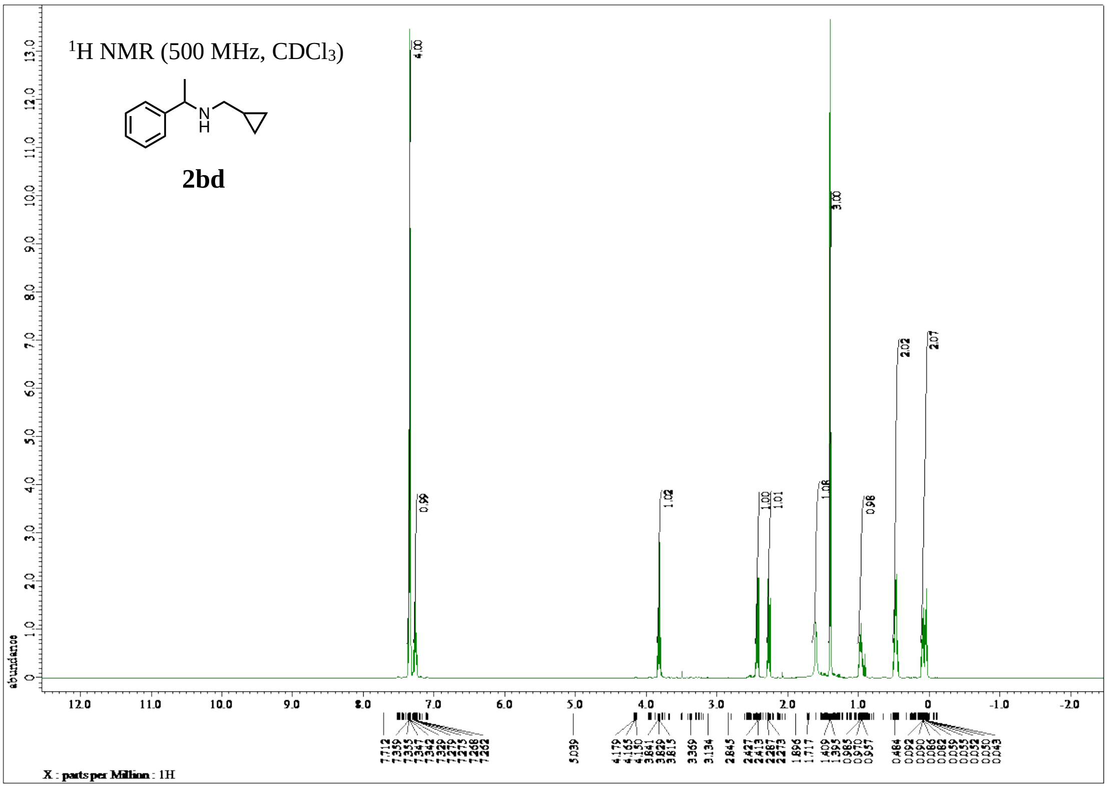

^1H NMR (500 MHz, $CDCl_3$)
2bd
X : parts per Million : 1H

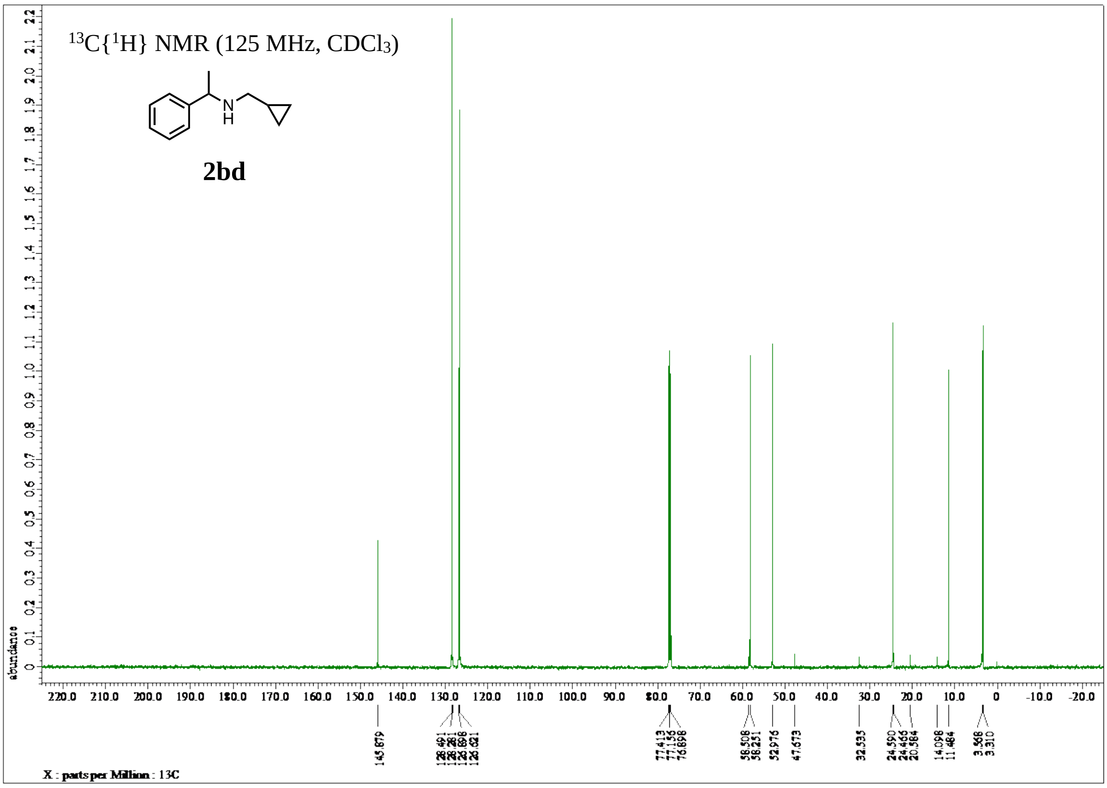

$^{13}C\{^1H\}$ NMR (125 MHz, $CDCl_3$)
2bd
X : parts per Million : 13C

^{1}H NMR (500 MHz, $CDCl_3$)

2be

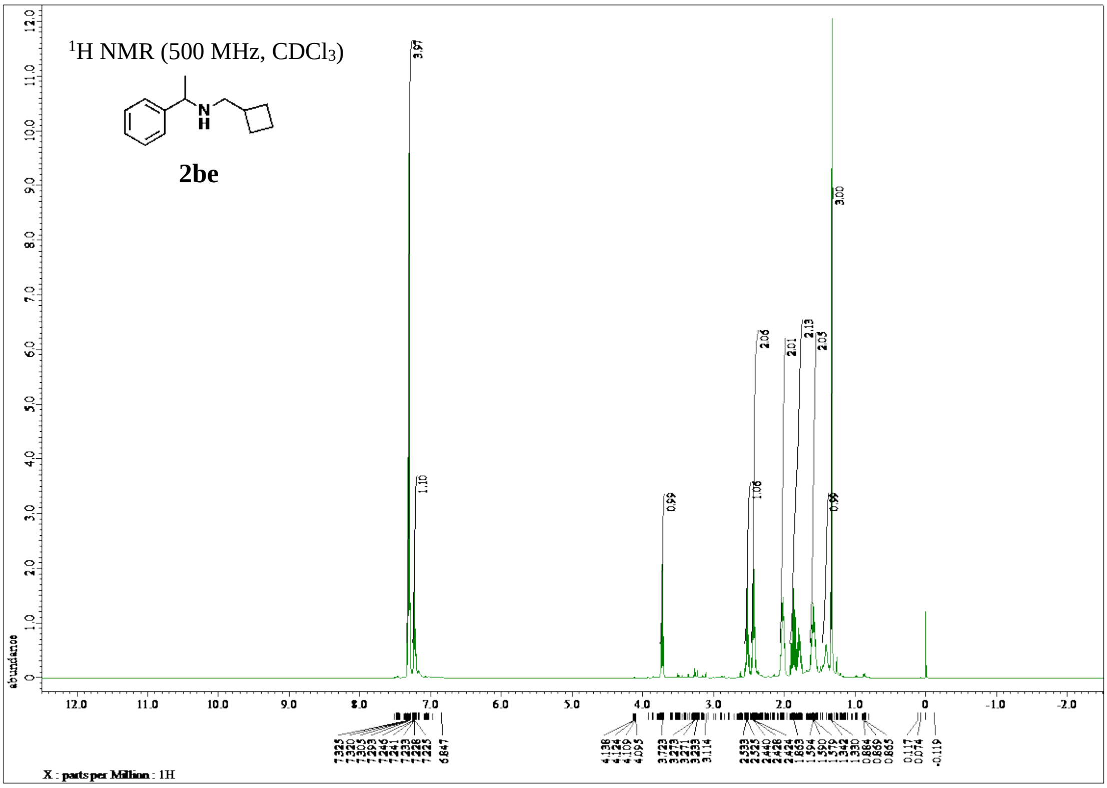

$^{13}C\{^{1}H\}$ NMR (125 MHz, $CDCl_3$)

2be

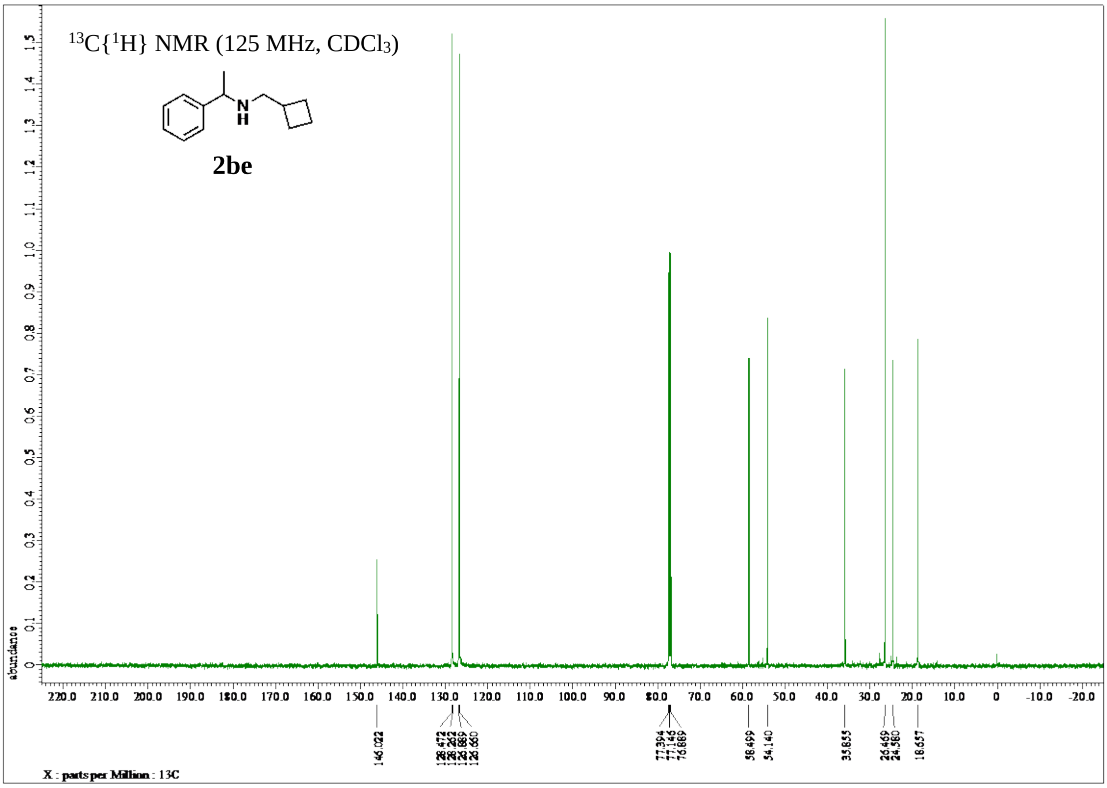

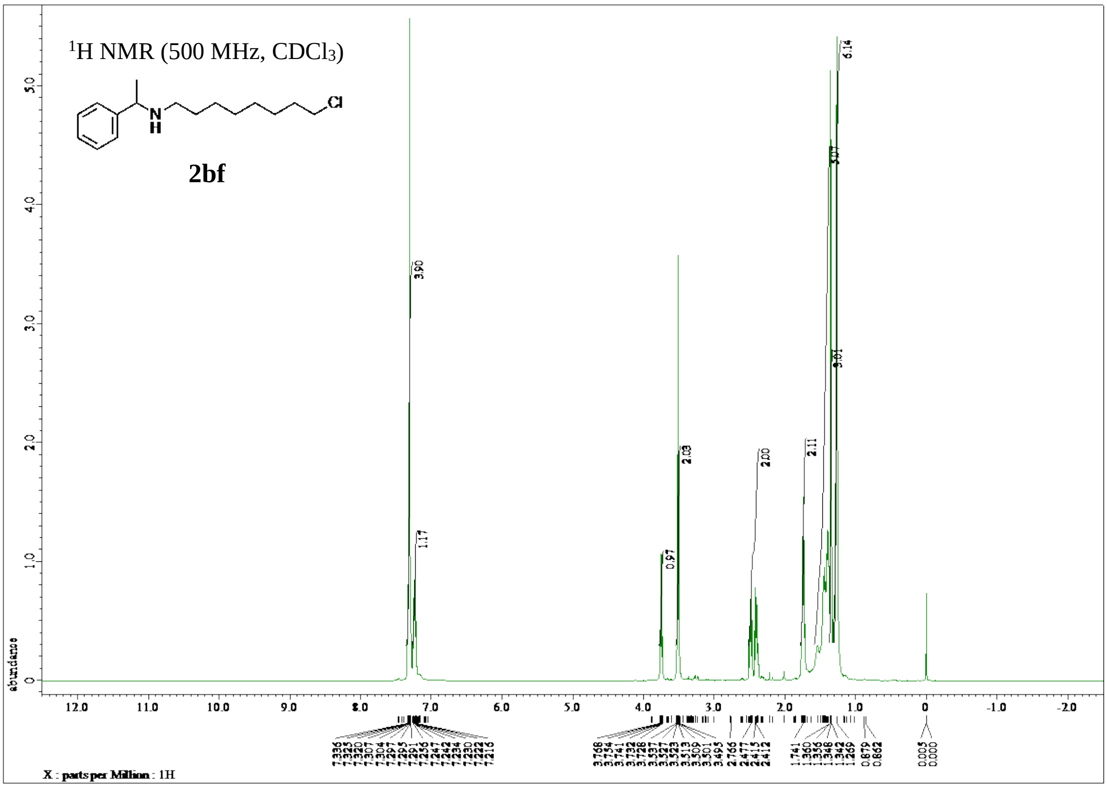
1H NMR (500 MHz, CDCl3)
2bf
X : parts per Million : 1H

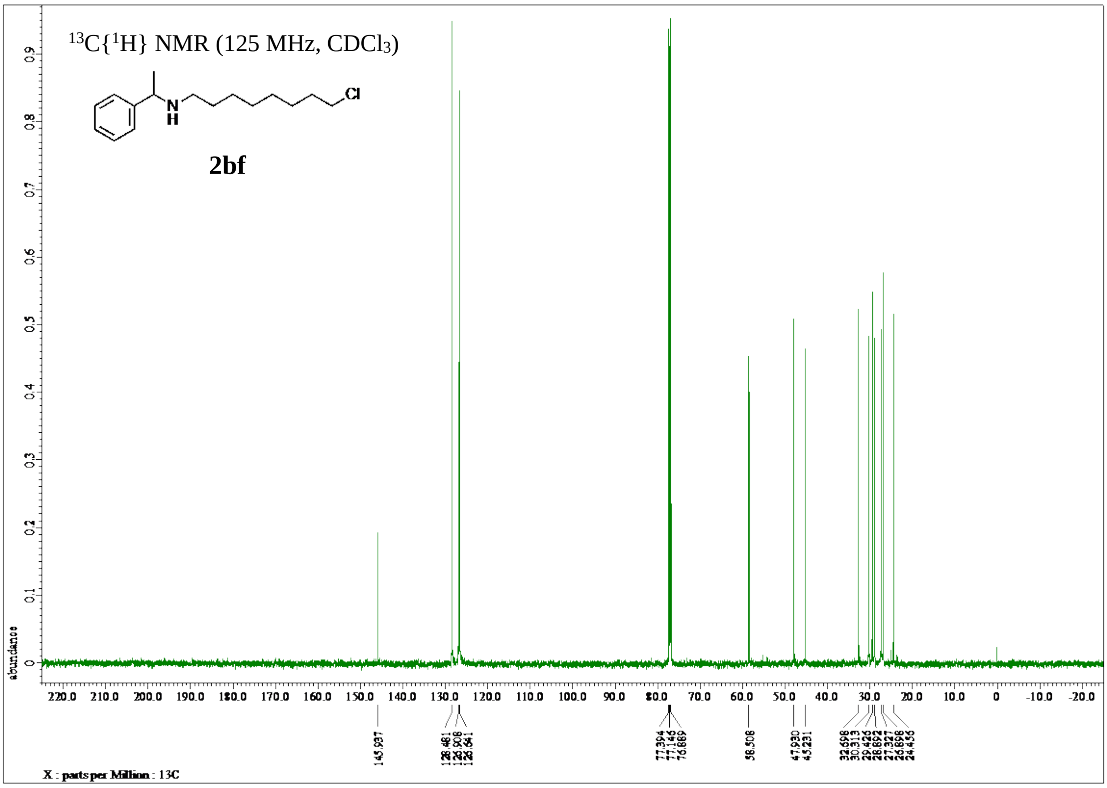
13C{1H} NMR (125 MHz, CDCl3)
2bf
X : parts per Million : 13C

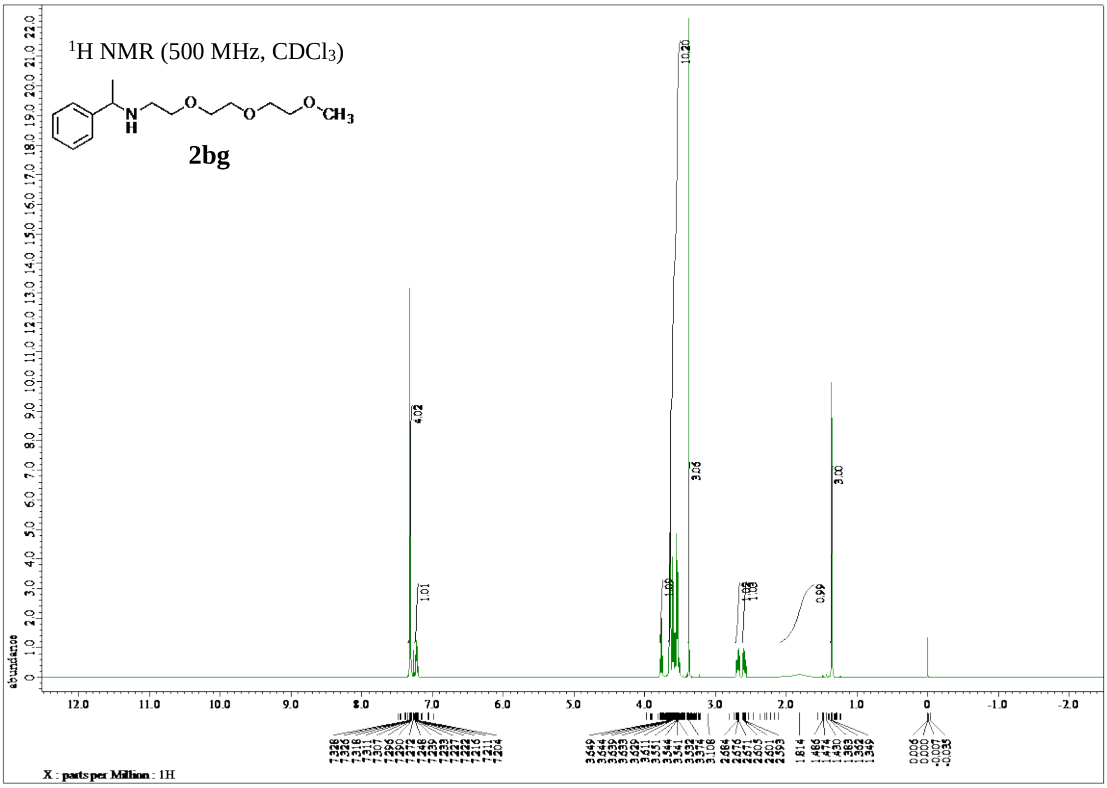
1H NMR (500 MHz, CDCl3)
2bg
X : parts per Million : 1H

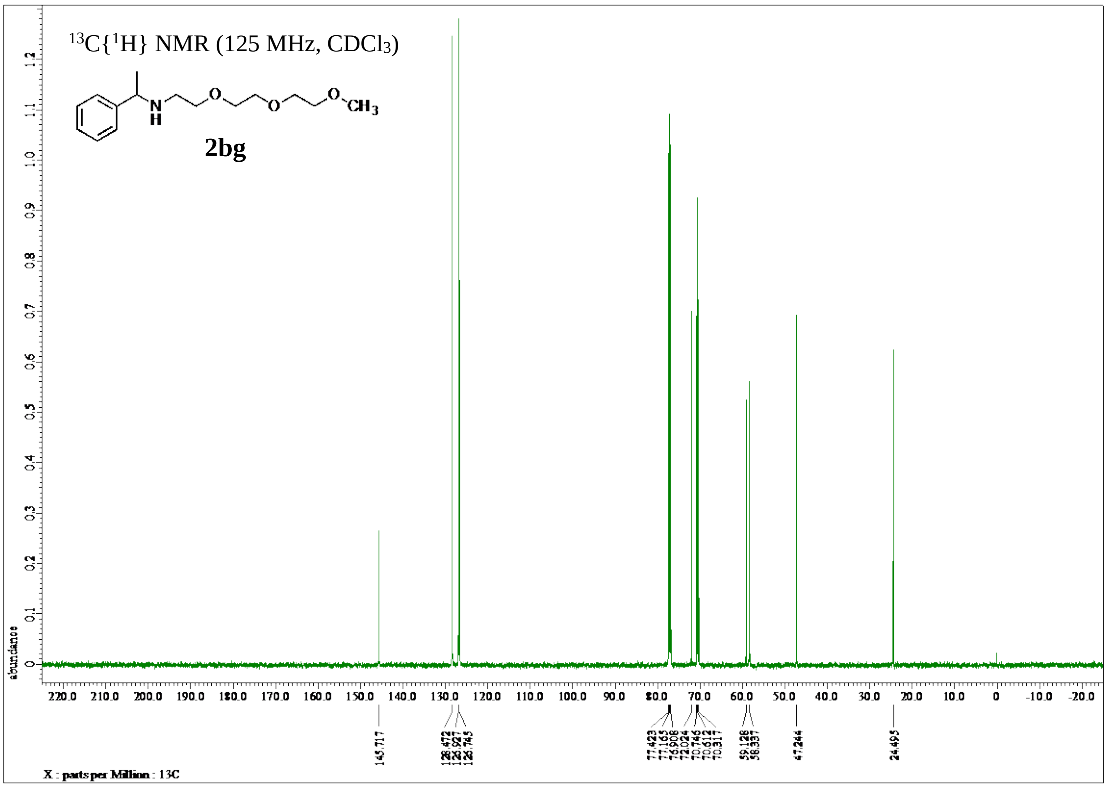
13C{1H} NMR (125 MHz, CDCl3)
2bg
X : parts per Million : 13C

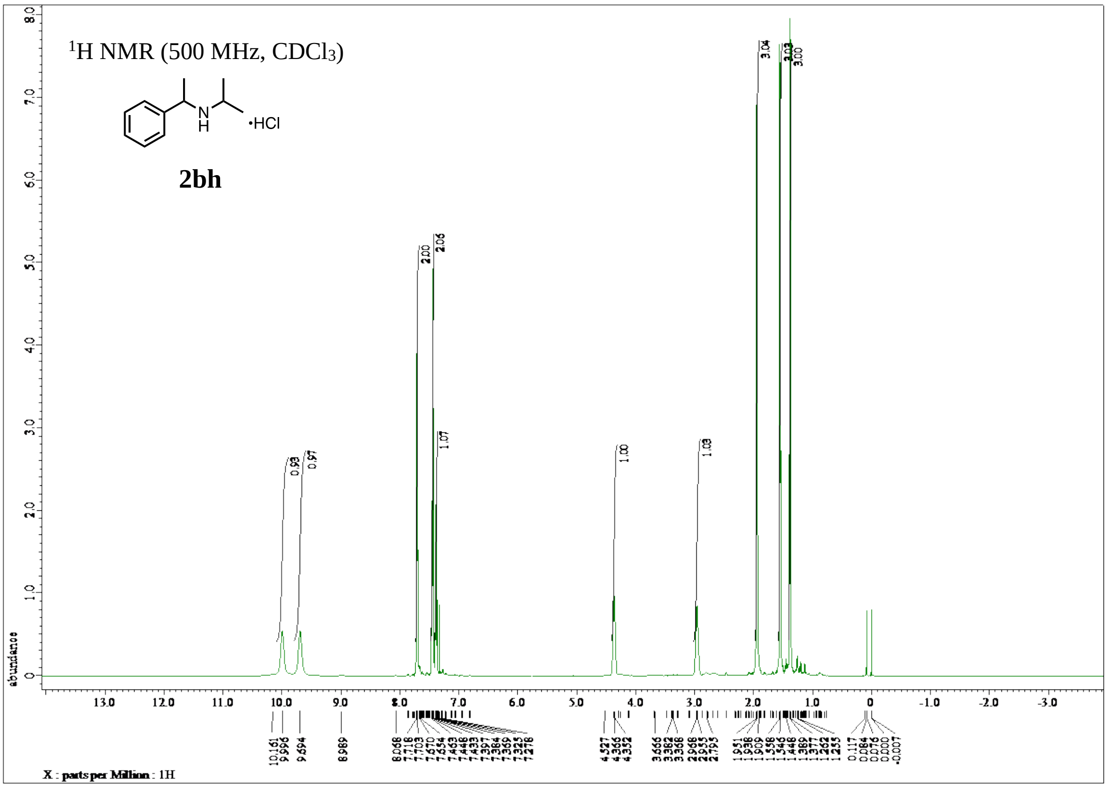

^{1}H NMR (500 MHz, CDCl$_3$)
•HCl
2bh
X : parts per Million : 1H
abundance

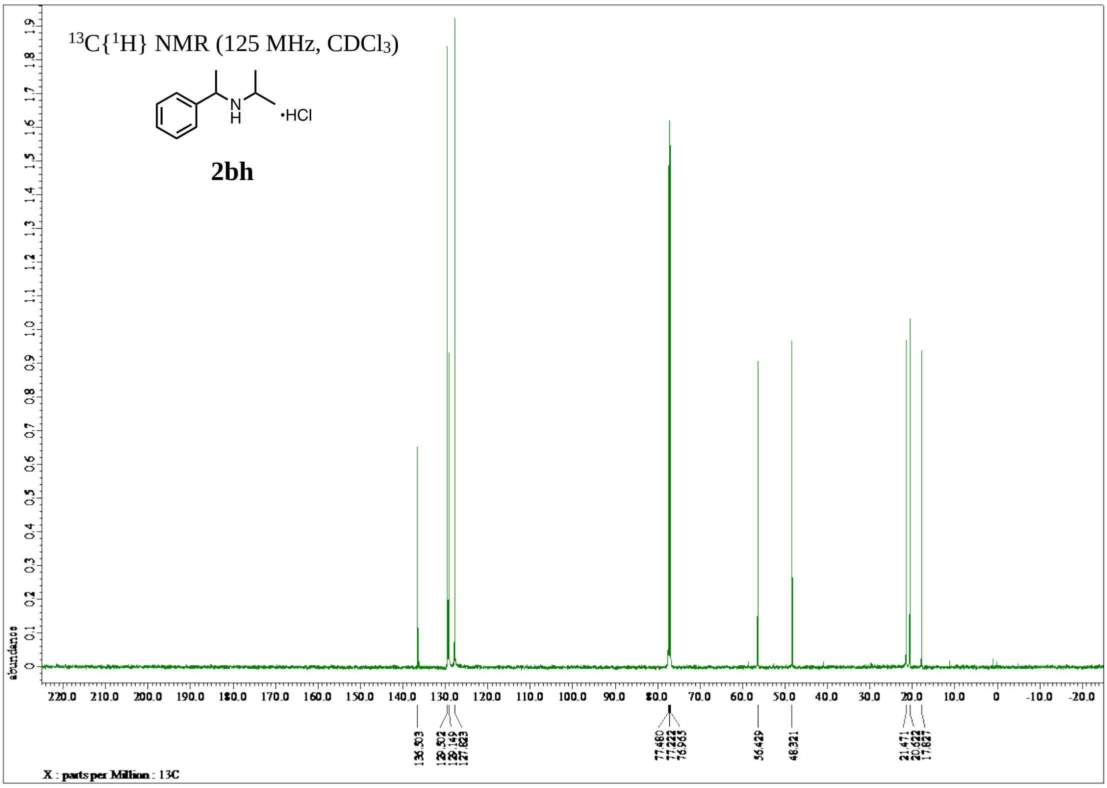

^{13}C{^{1}H} NMR (125 MHz, CDCl$_3$)
•HCl
2bh
X : parts per Million : 13C
abundance

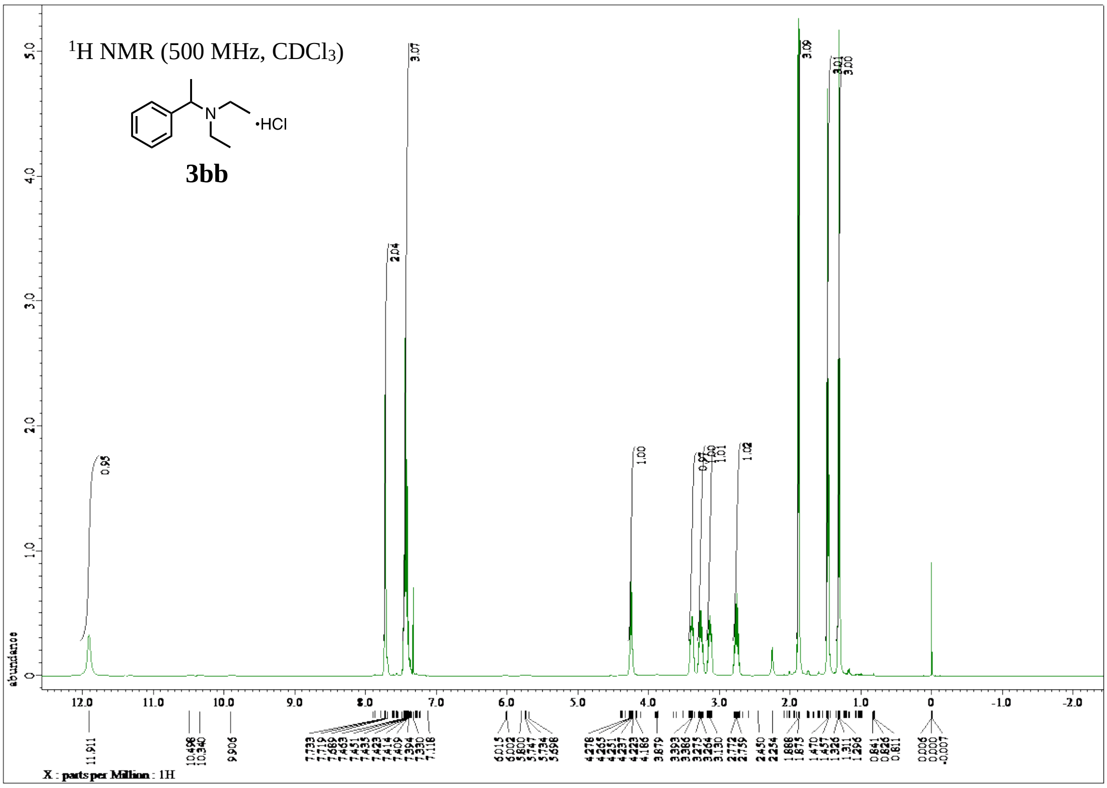
1H NMR (500 MHz, CDCl3)
•HCl
3bb
X : parts per Million : 1H

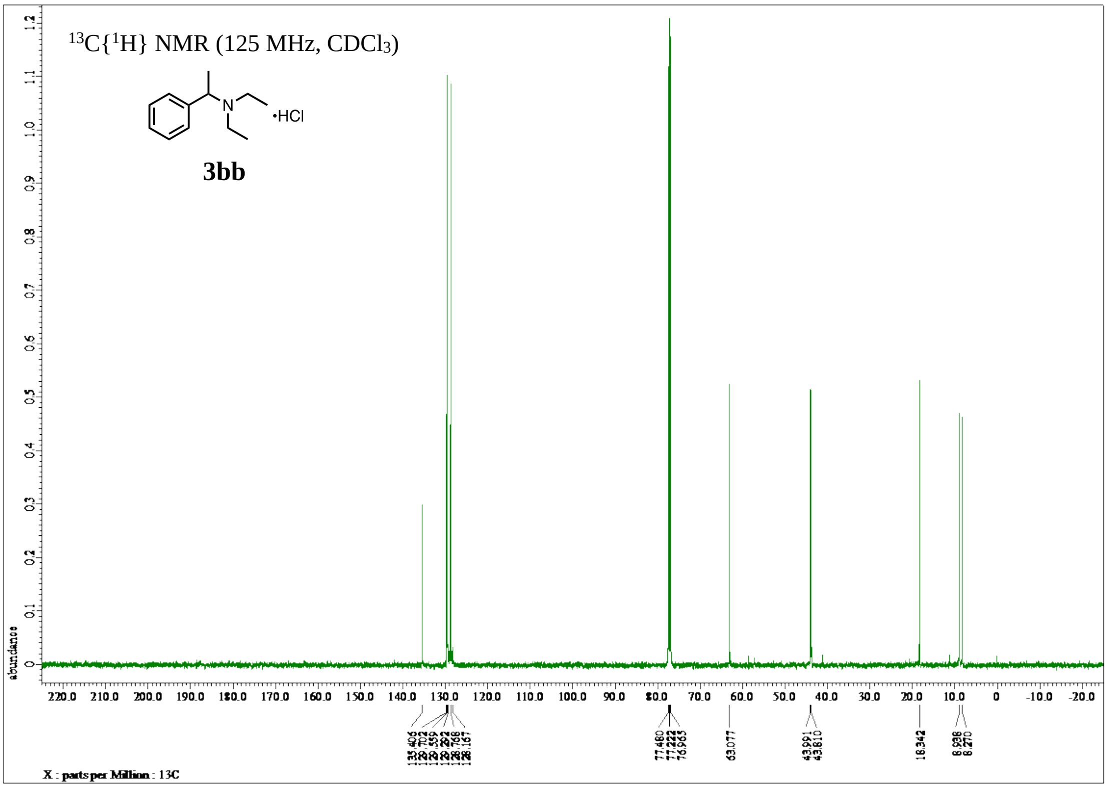
13C{1H} NMR (125 MHz, CDCl3)
•HCl
3bb
X : parts per Million : 13C

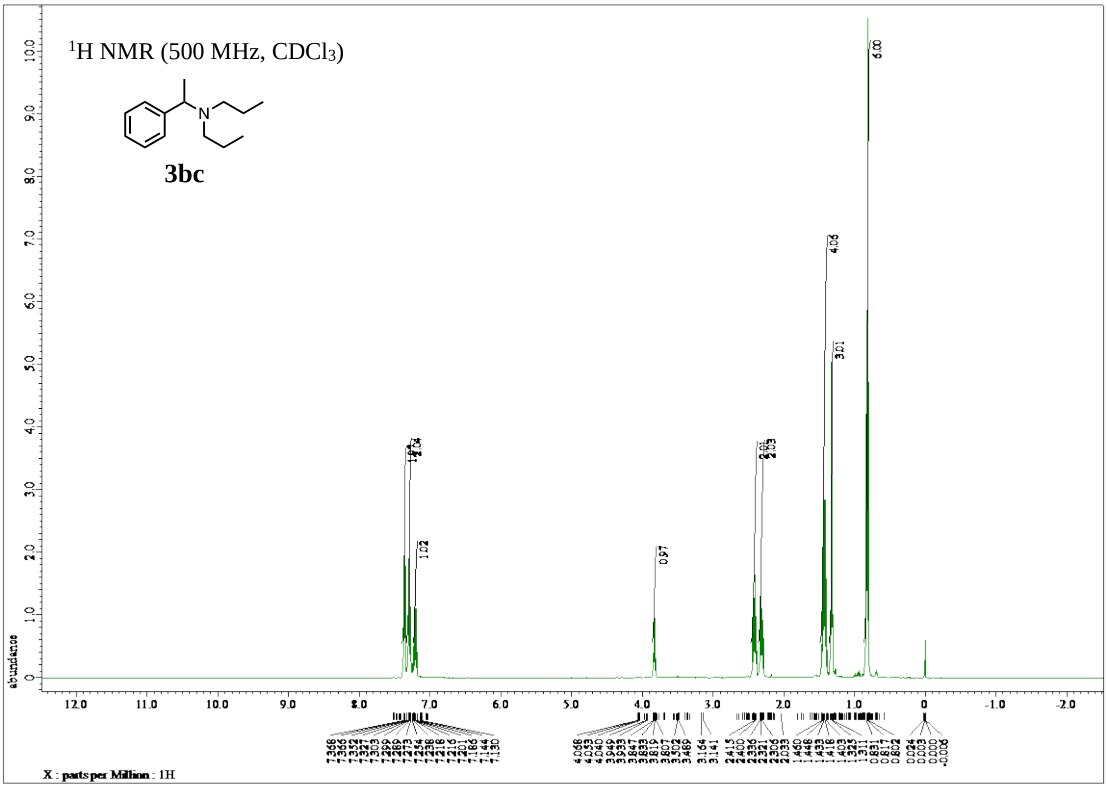

1H NMR (500 MHz, CDCl3)
3bc
abundance
X : parts per Million : 1H

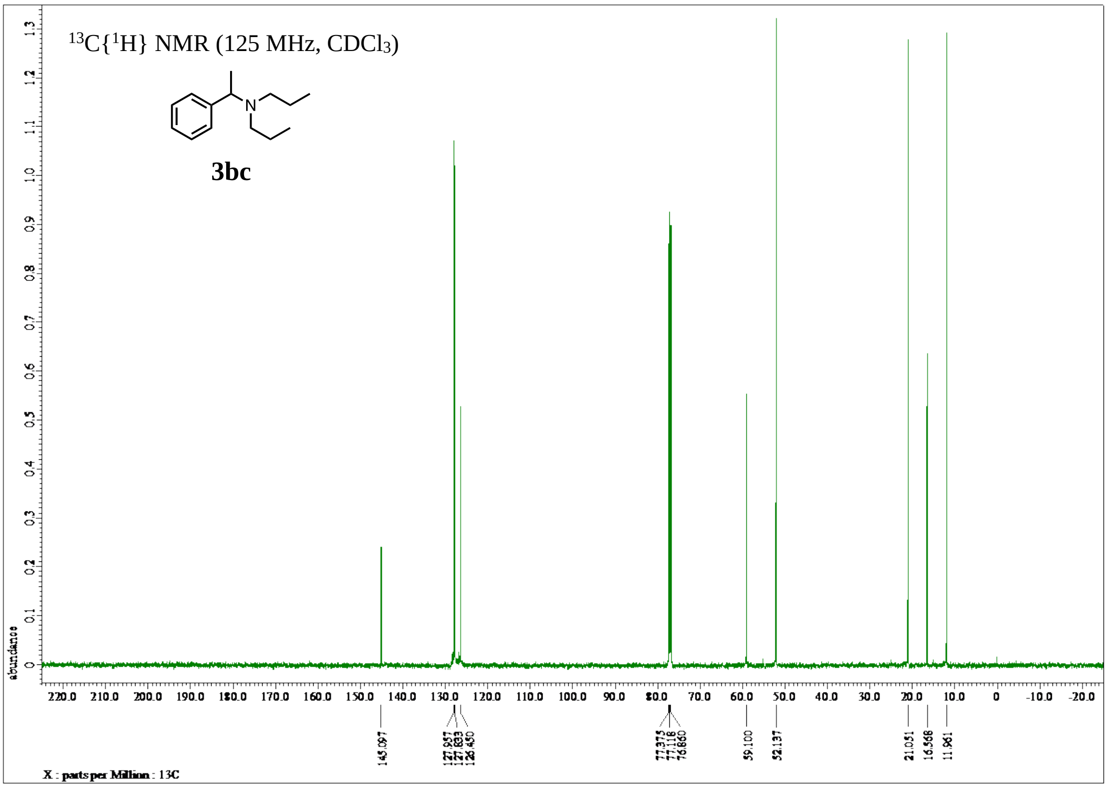

13C{1H} NMR (125 MHz, CDCl3)
3bc
abundance
X : parts per Million : 13C

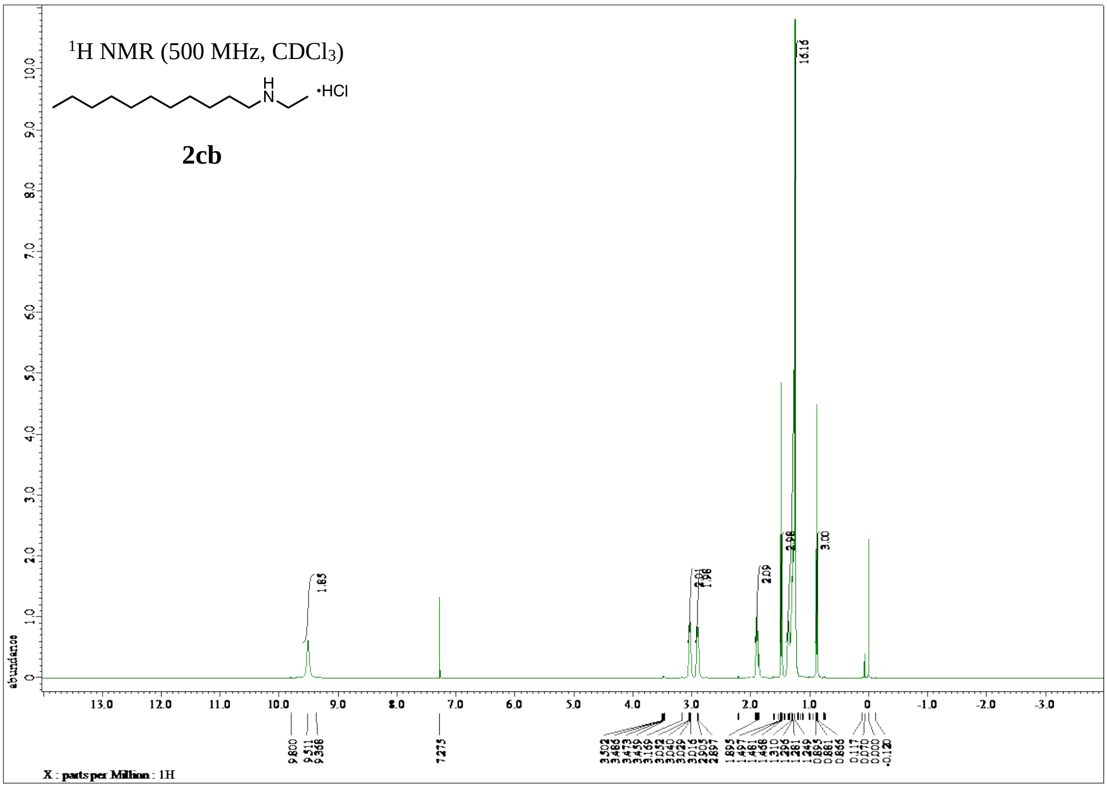
1H NMR (500 MHz, CDCl3)
•HCl
2cb
X : parts per Million : 1H
abundance

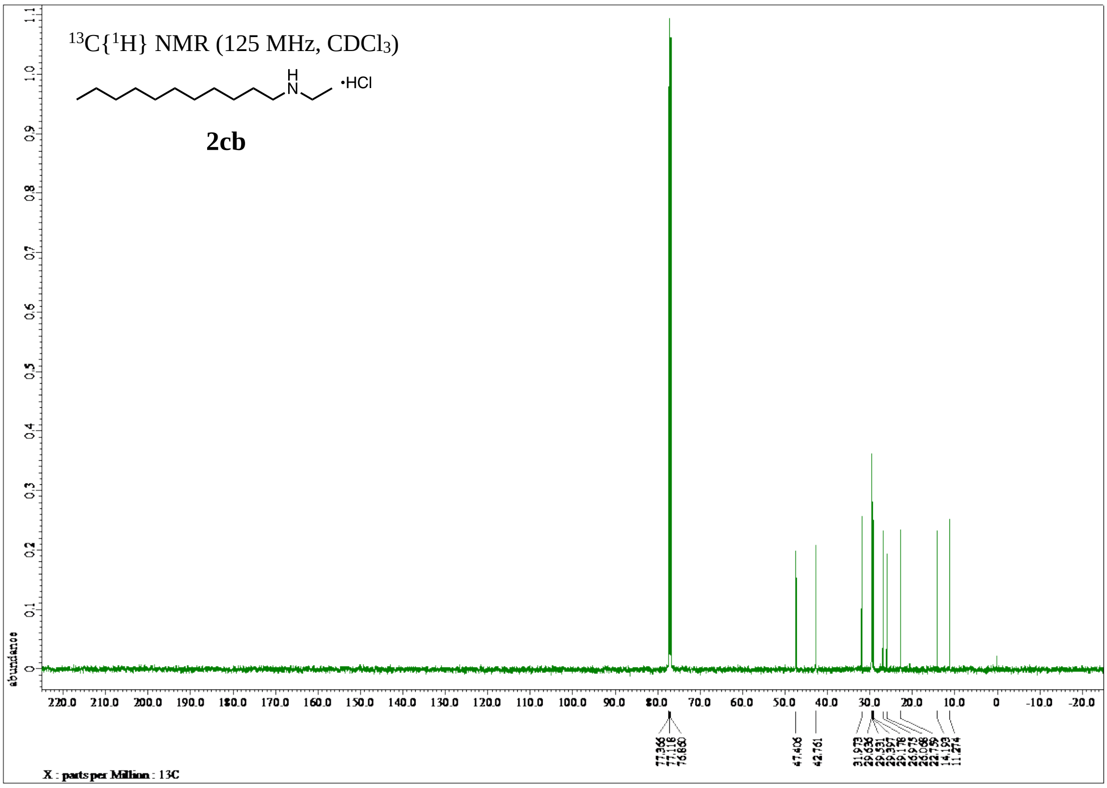
13C{1H} NMR (125 MHz, CDCl3)
•HCl
2cb
X : parts per Million : 13C
abundance

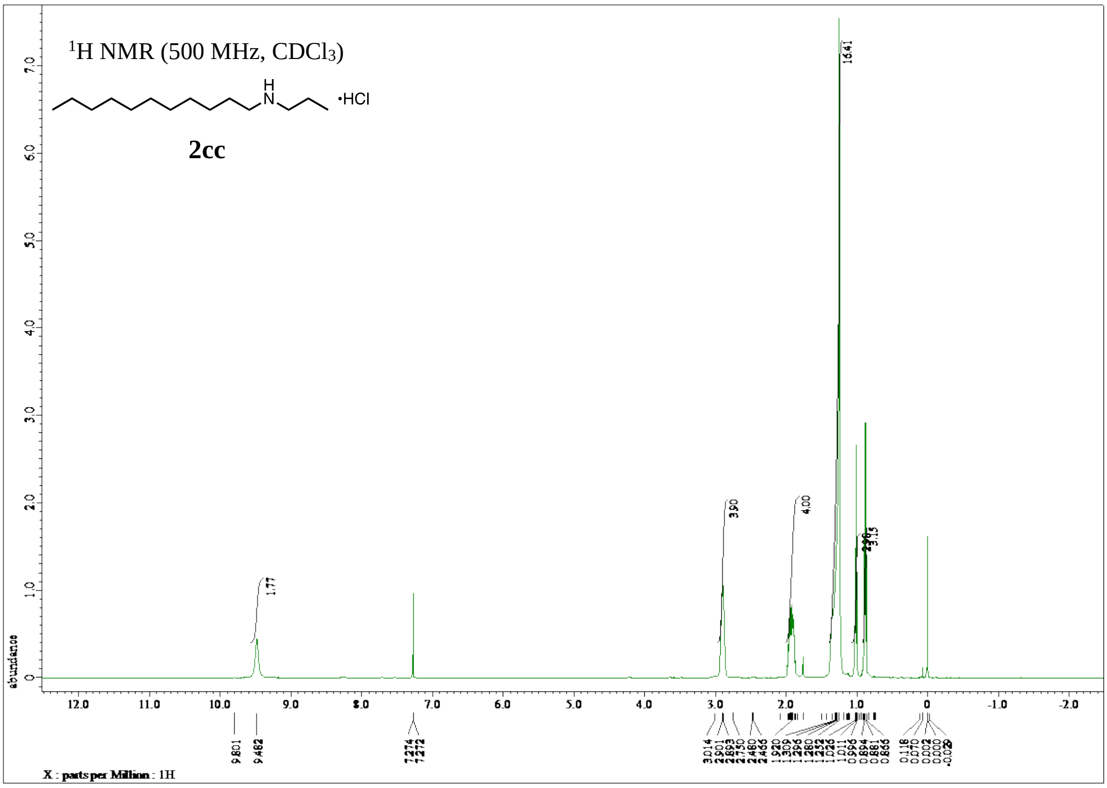

^{1}H NMR (500 MHz, CDCl$_3$)
H
N
•HCl
2cc
X : parts per Million : 1H
abundance

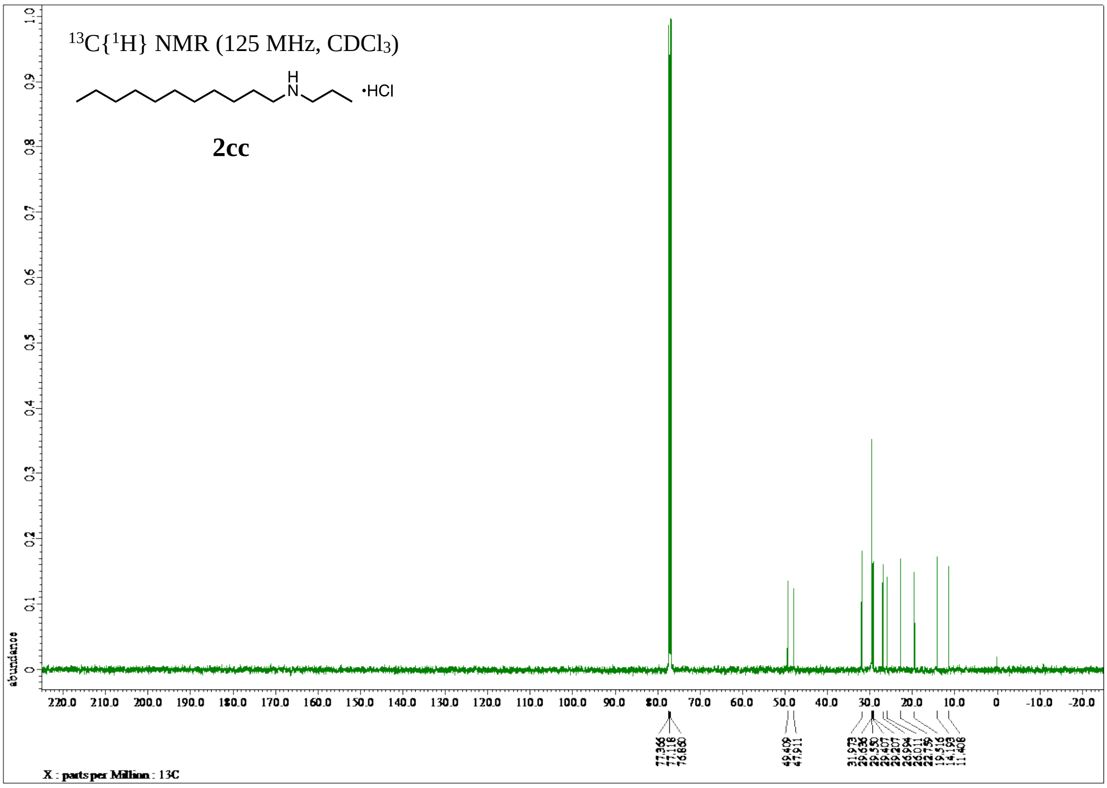

^{13}C{^{1}H} NMR (125 MHz, CDCl$_3$)
H
N
•HCl
2cc
X : parts per Million : 13C
abundance

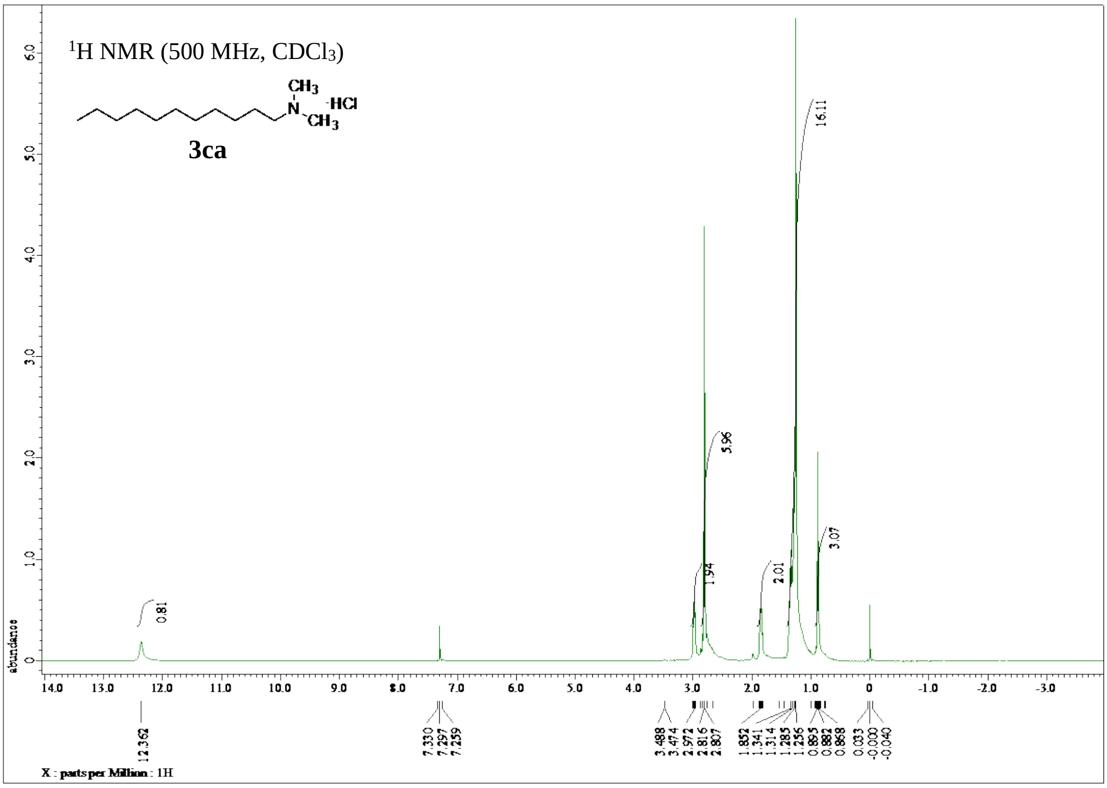

^{1}H NMR (500 MHz, CDCl3)
CH3
N
·HCl
CH3
3ca
X : parts per Million : 1H

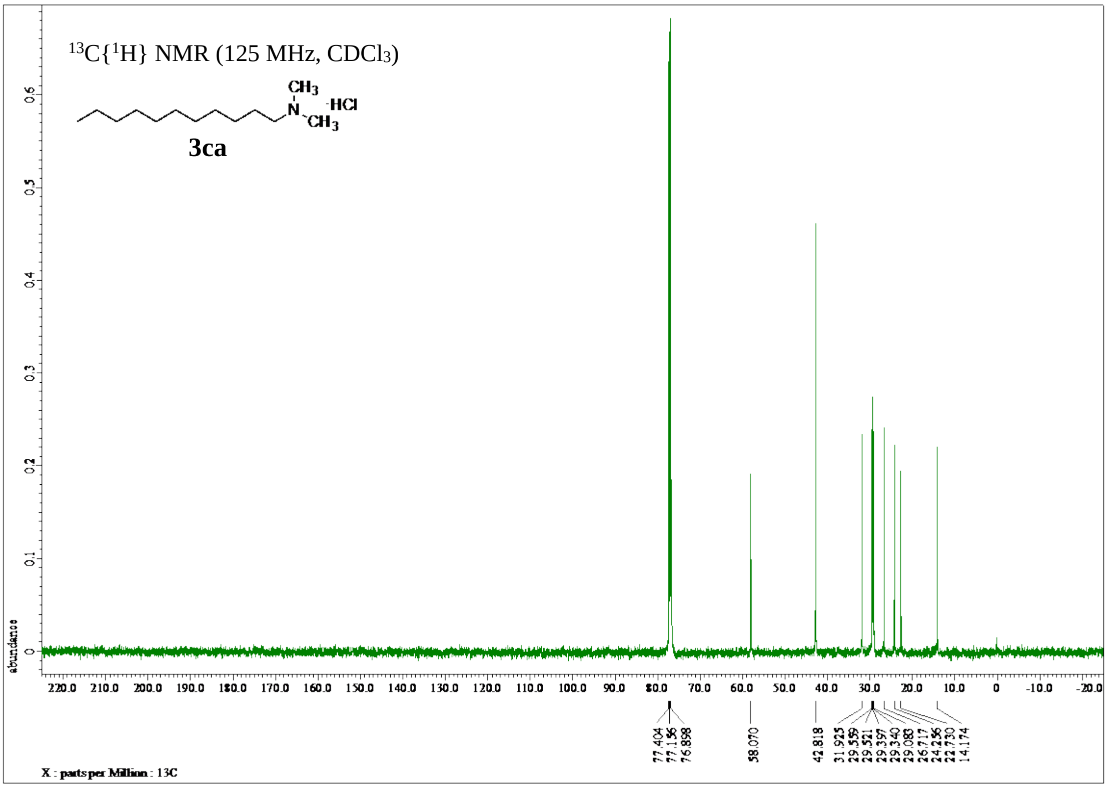

^{13}C{^{1}H} NMR (125 MHz, CDCl3)
CH3
N
·HCl
CH3
3ca
X : parts per Million : 13C

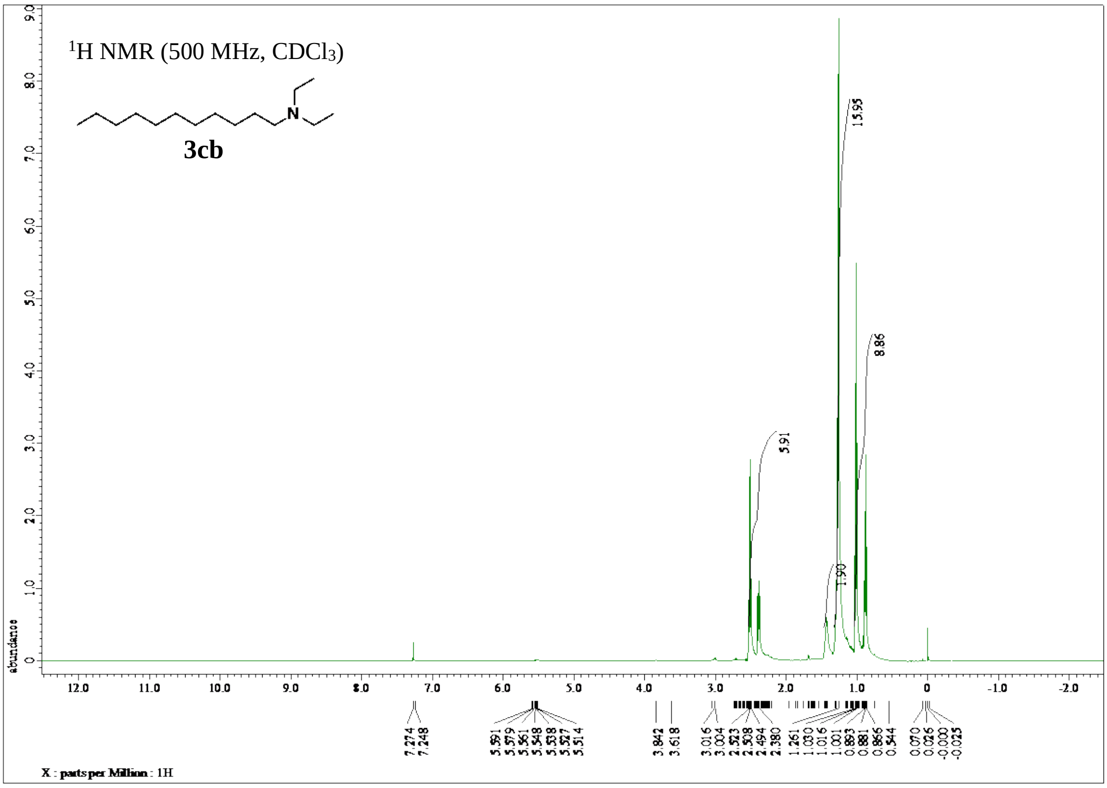

1H NMR (500 MHz, CDCl3)
N
3cb
X : parts per Million : 1H

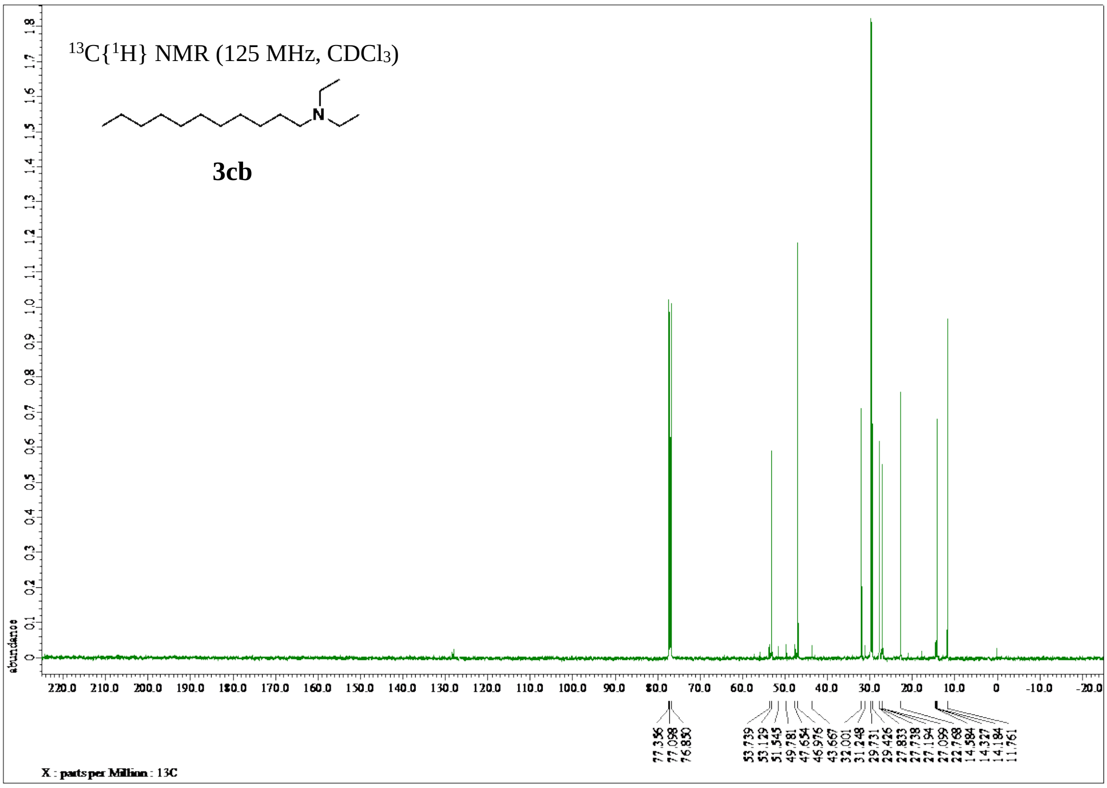

13C{1H} NMR (125 MHz, CDCl3)
N
3cb
X : parts per Million : 13C

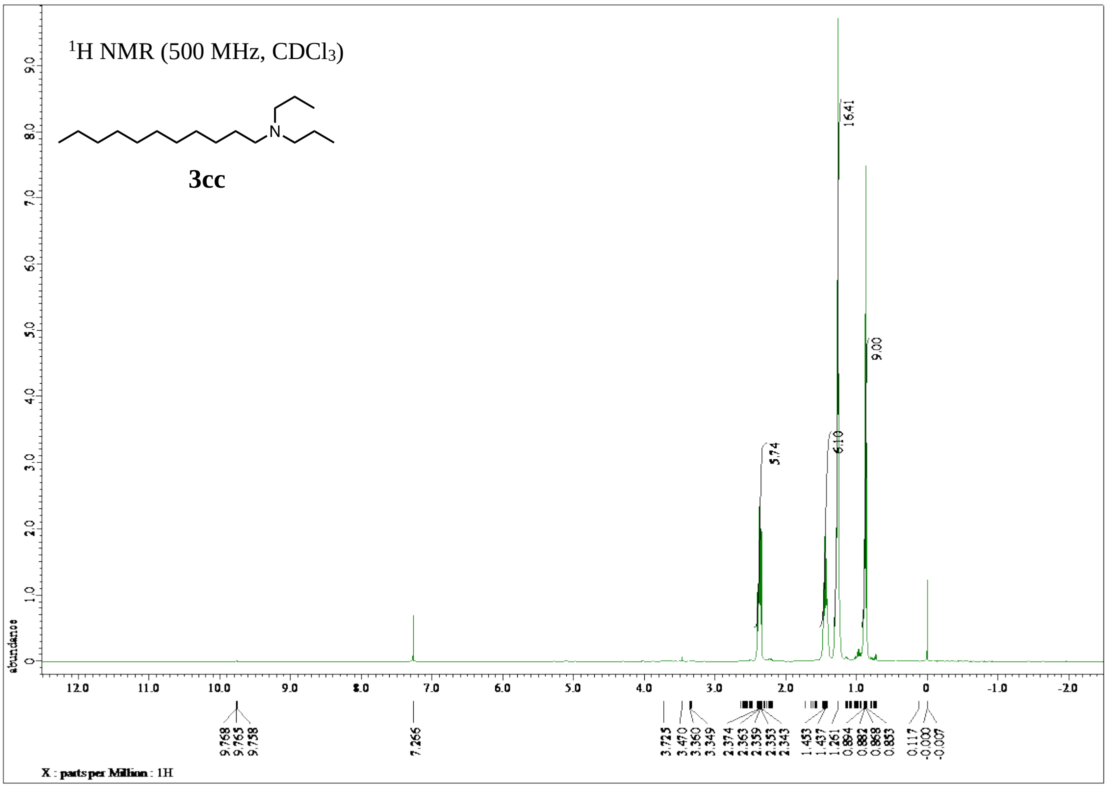

¹H NMR (500 MHz, CDCl3)
3cc
N
16.41
9.00
6.10
5.74
X : parts per Million : 1H

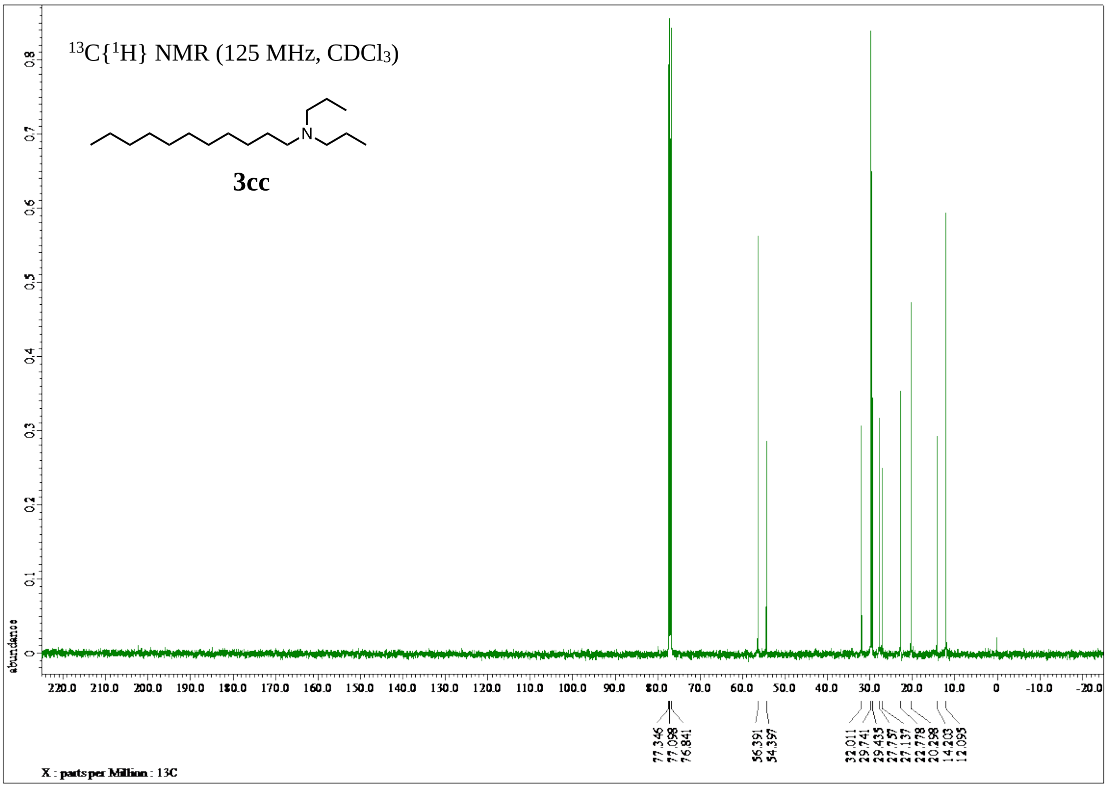

¹³C{¹H} NMR (125 MHz, CDCl3)
3cc
N
X : parts per Million : 13C

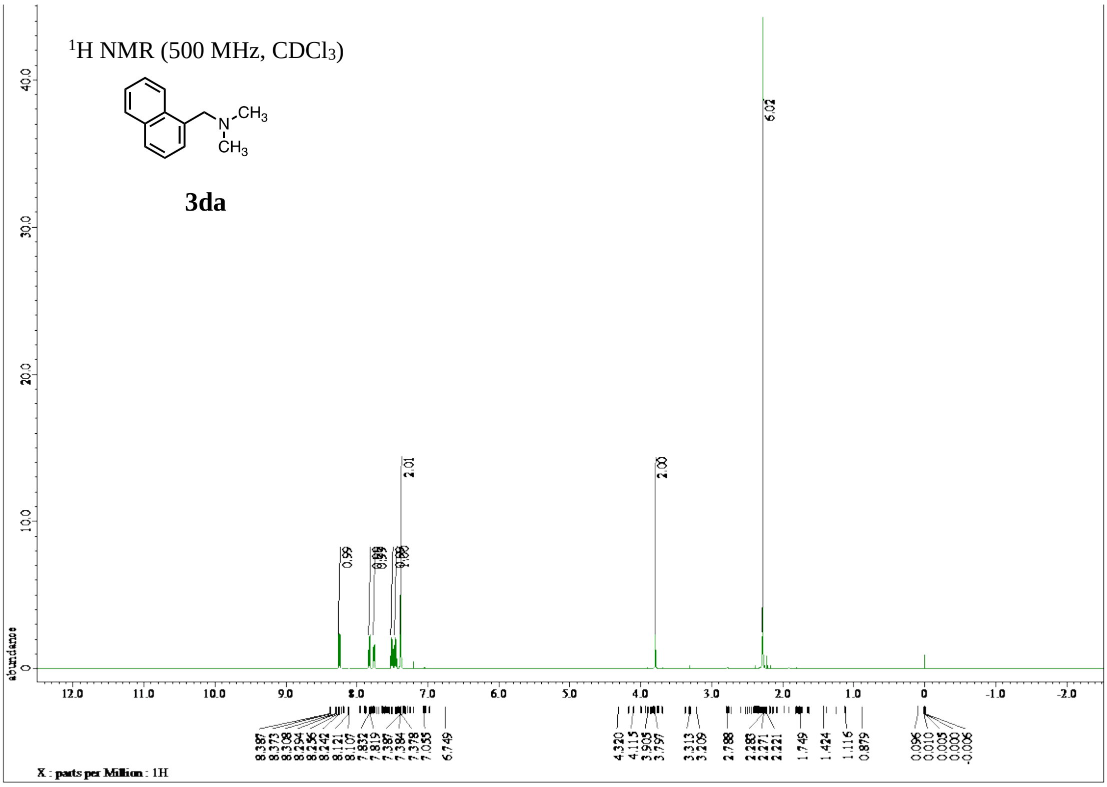
^{1}H NMR (500 MHz, $CDCl_3$)
CH_3
N
CH_3
3da
X : parts per Million : 1H

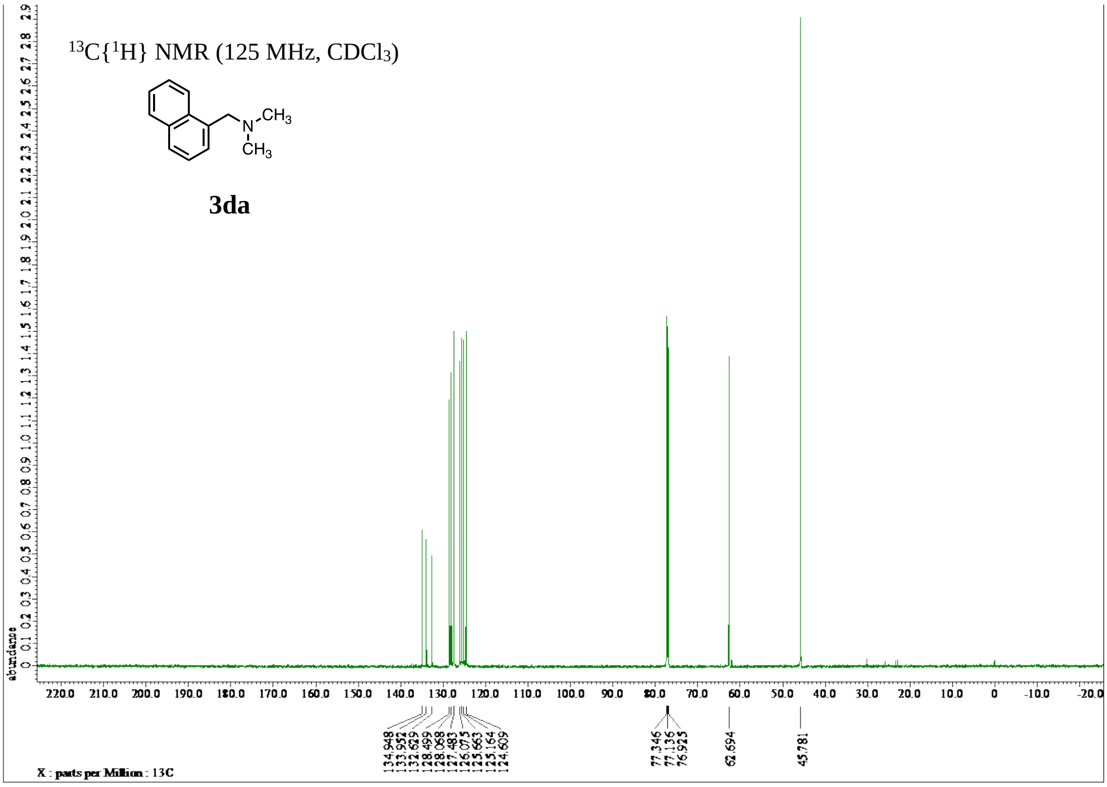
$^{13}C\{^{1}H\}$ NMR (125 MHz, $CDCl_3$)
CH_3
N
CH_3
3da
X : parts per Million : 13C

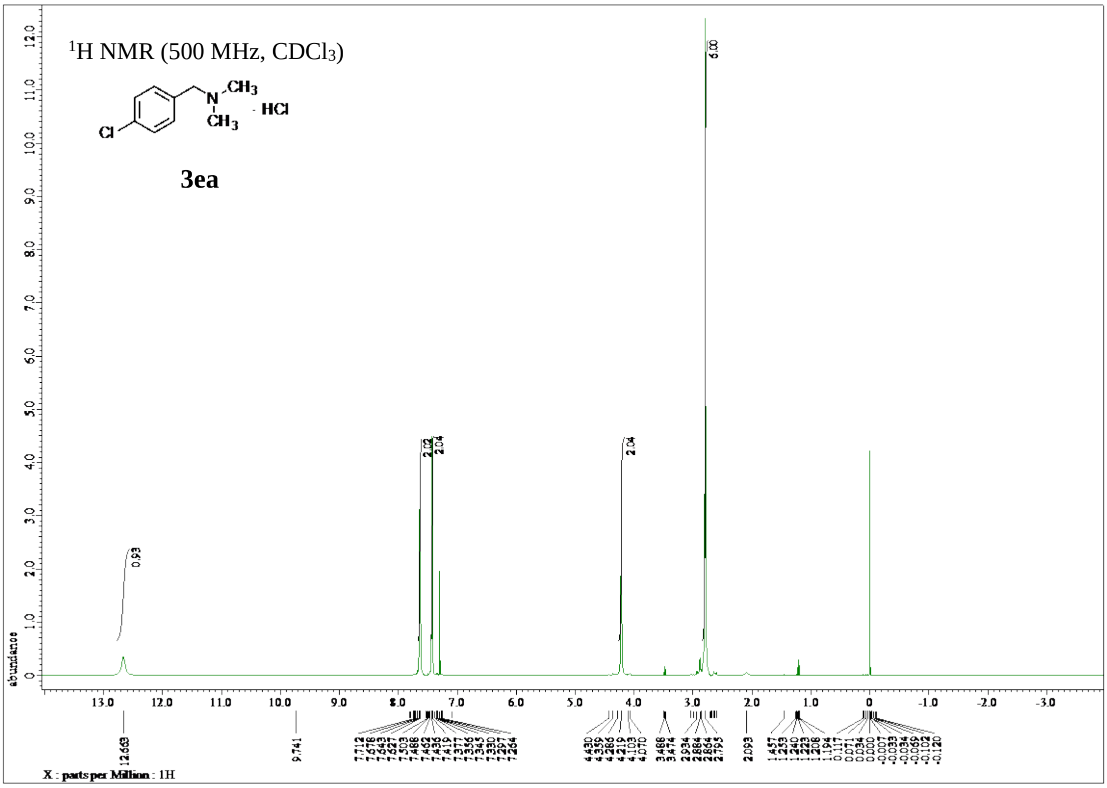

^{1}H NMR (500 MHz, CDCl$_3$)
3ea
X : parts per Million : 1H
abundance

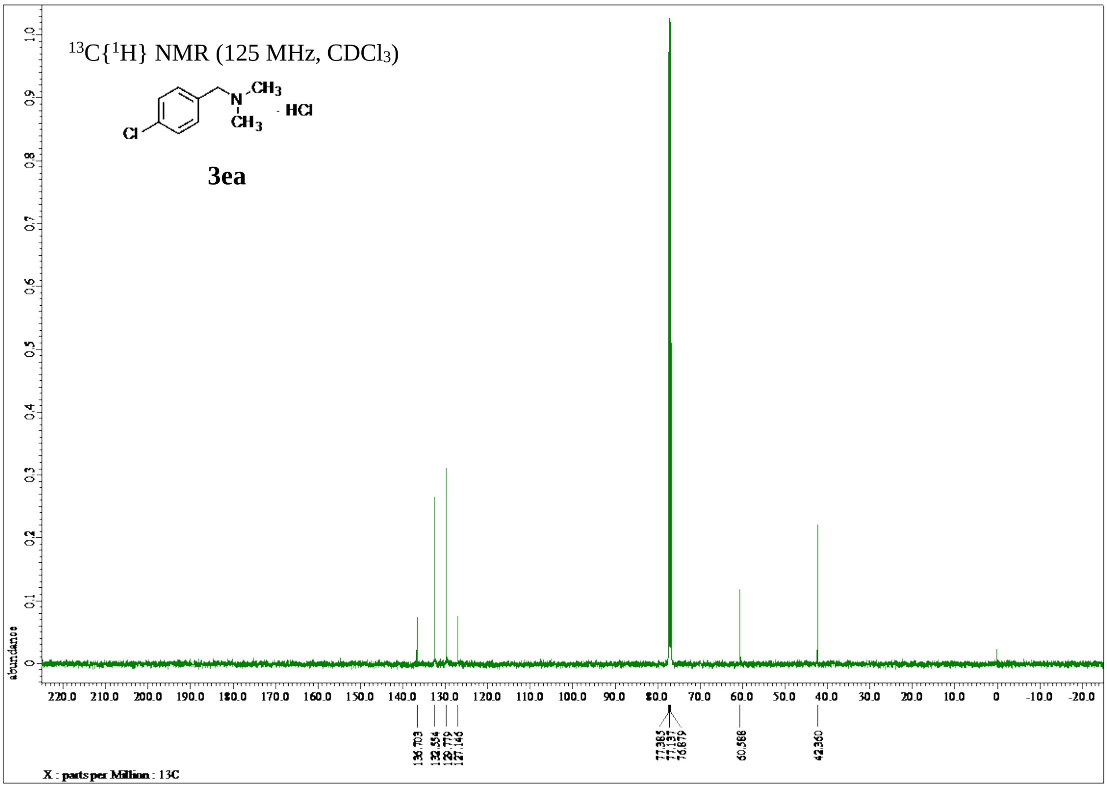

^{13}C{^{1}H} NMR (125 MHz, CDCl$_3$)
3ea
X : parts per Million : 13C
abundance

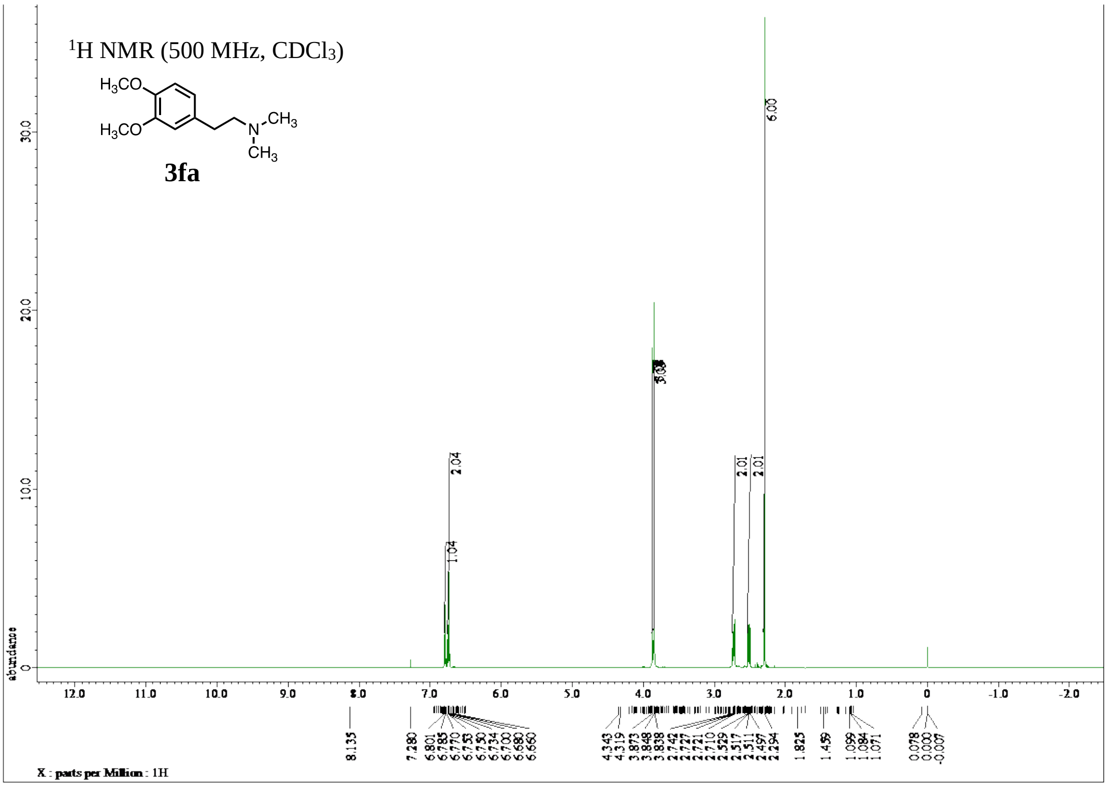

^{1}H NMR (500 MHz, $CDCl_3$)
3fa
X : parts per Million : 1H
abundance

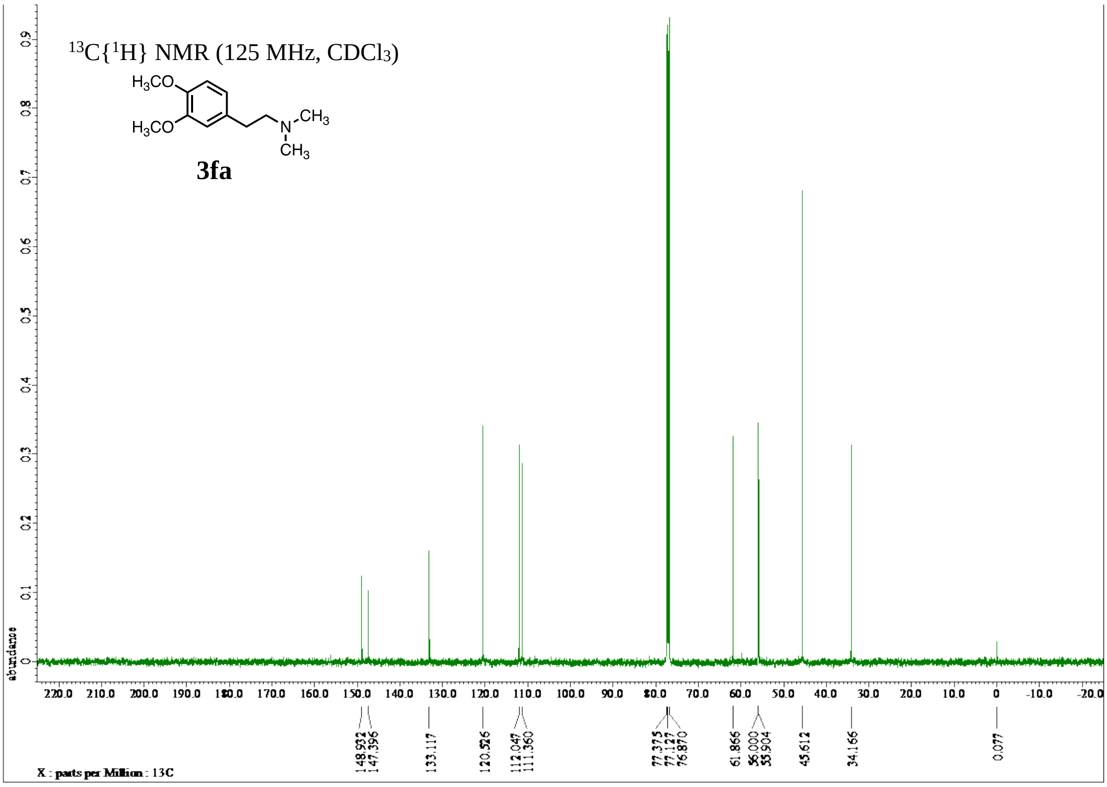

$^{13}C\{^{1}H\}$ NMR (125 MHz, $CDCl_3$)
3fa
X : parts per Million : 13C
abundance

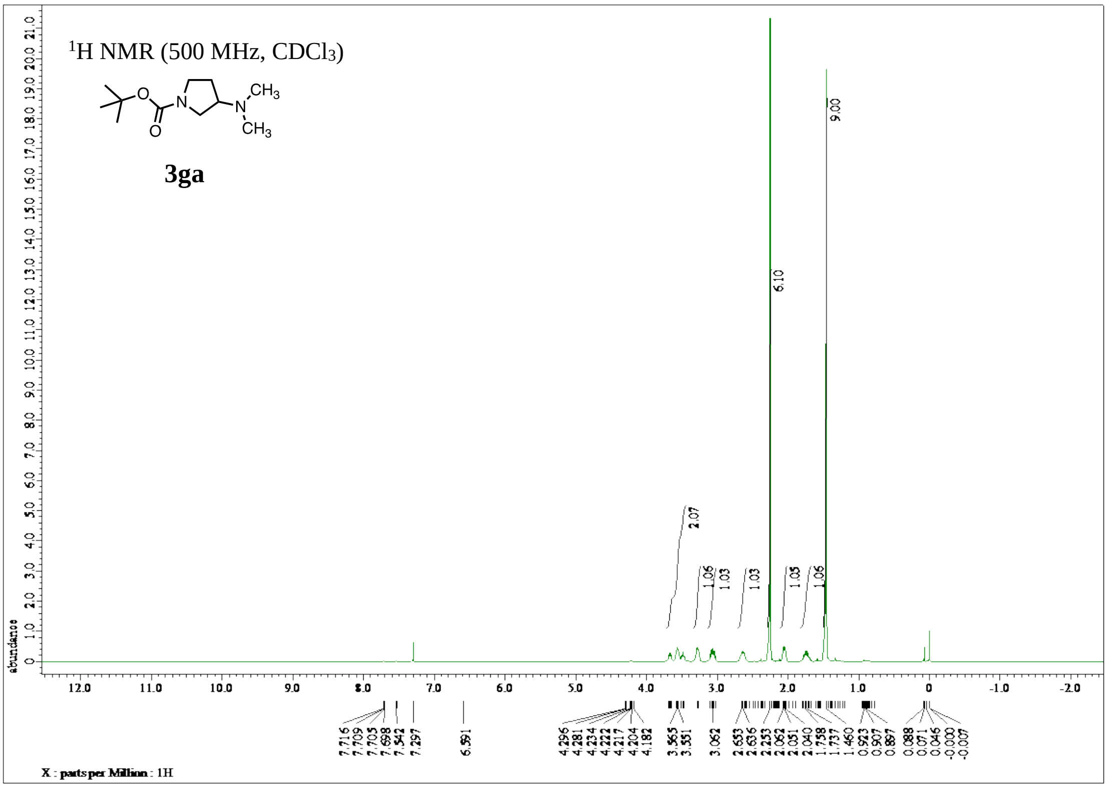

^{1}H NMR (500 MHz, CDCl3)
3ga
abundance
X : parts per Million : 1H

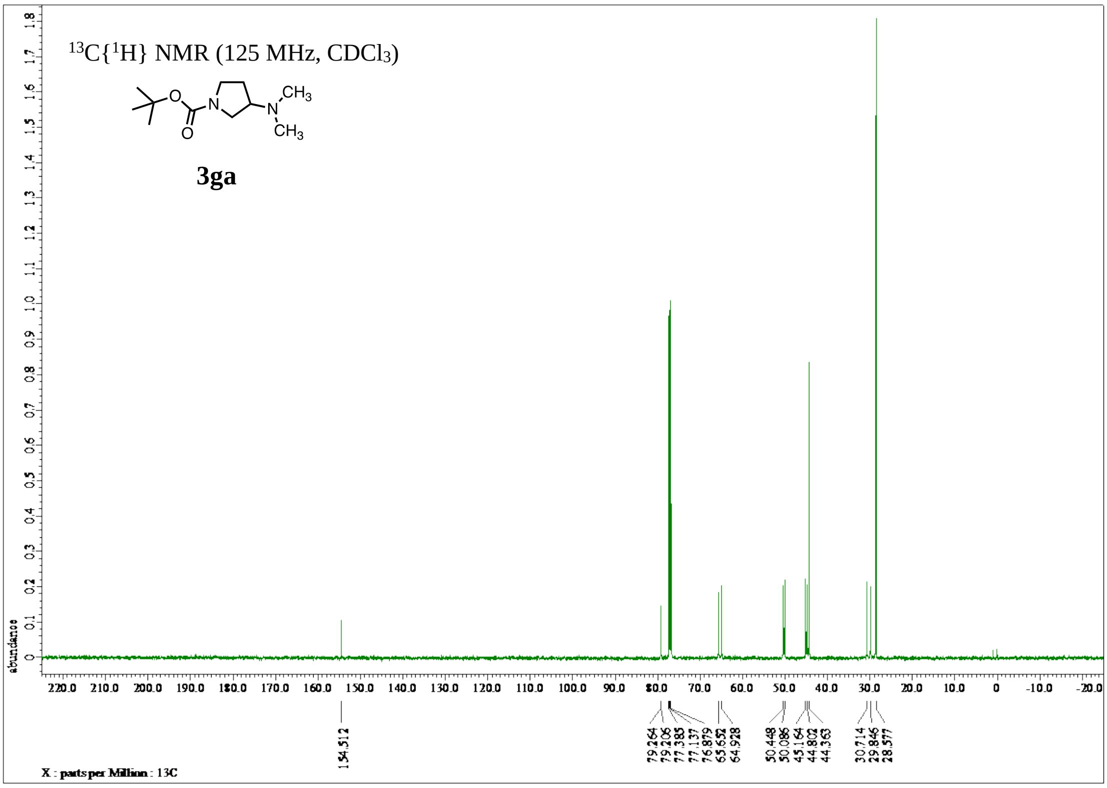

^{13}C{^{1}H} NMR (125 MHz, CDCl3)
3ga
abundance
X : parts per Million : 13C

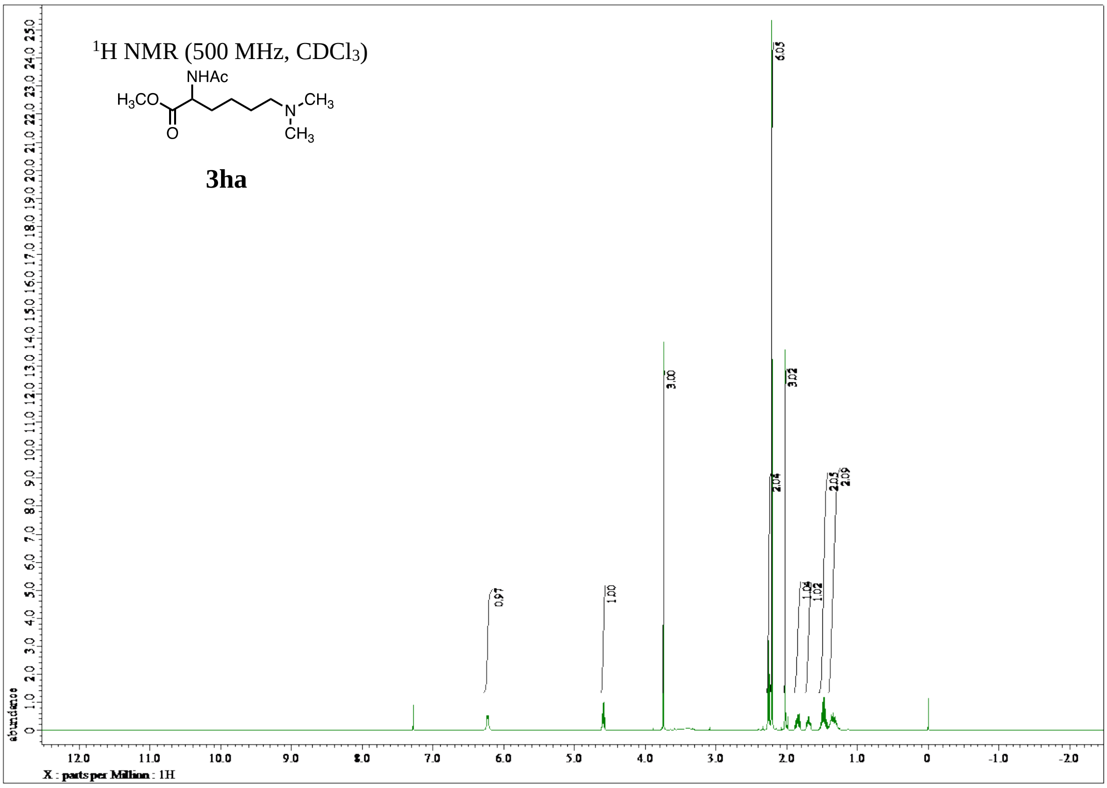

1H NMR (500 MHz, CDCl3)
NHAc
H3CO
N
CH3
O
CH3
3ha
abundance
X : parts per Million : 1H

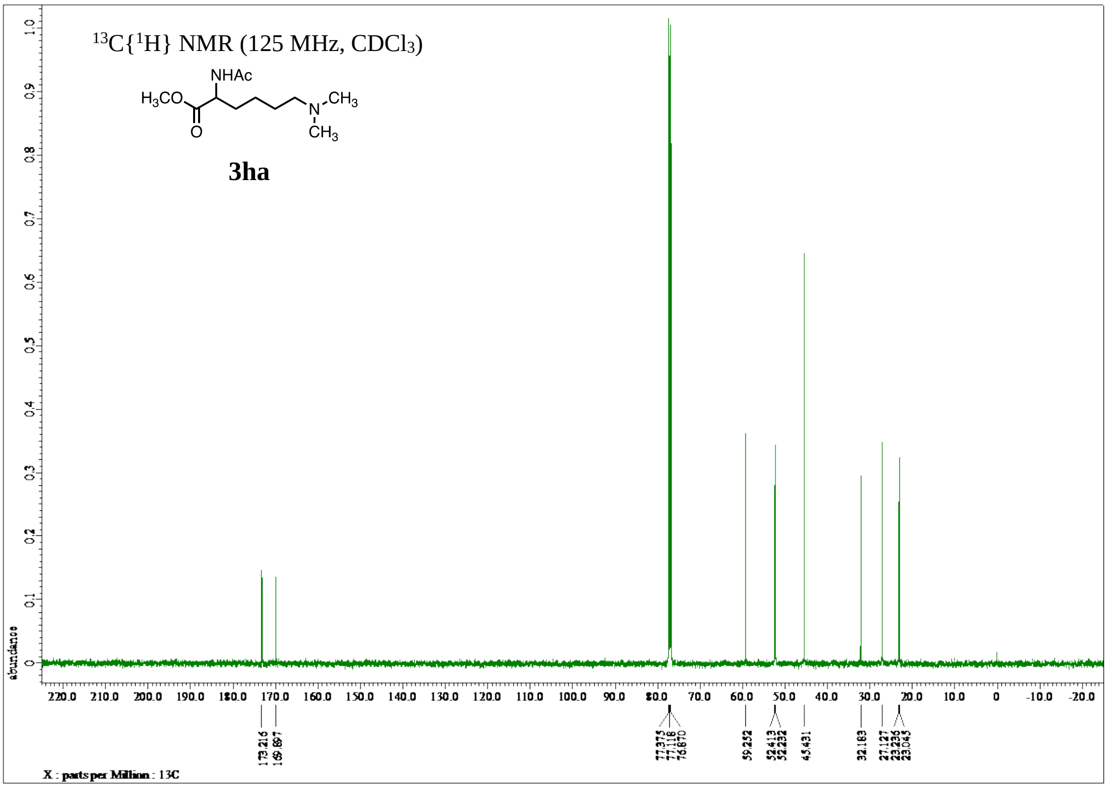

13C{1H} NMR (125 MHz, CDCl3)
NHAc
H3CO
N
CH3
O
CH3
3ha
abundance
X : parts per Million : 13C

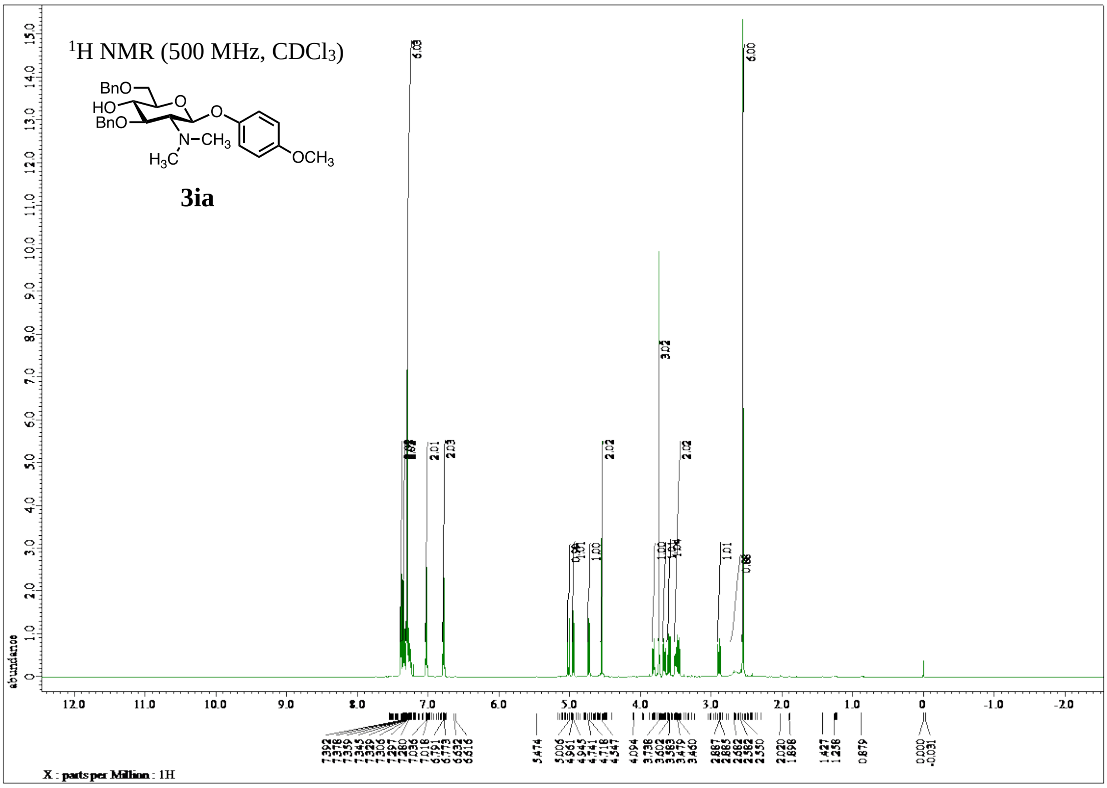

1H NMR (500 MHz, CDCl3)
3ia
X : parts per Million : 1H

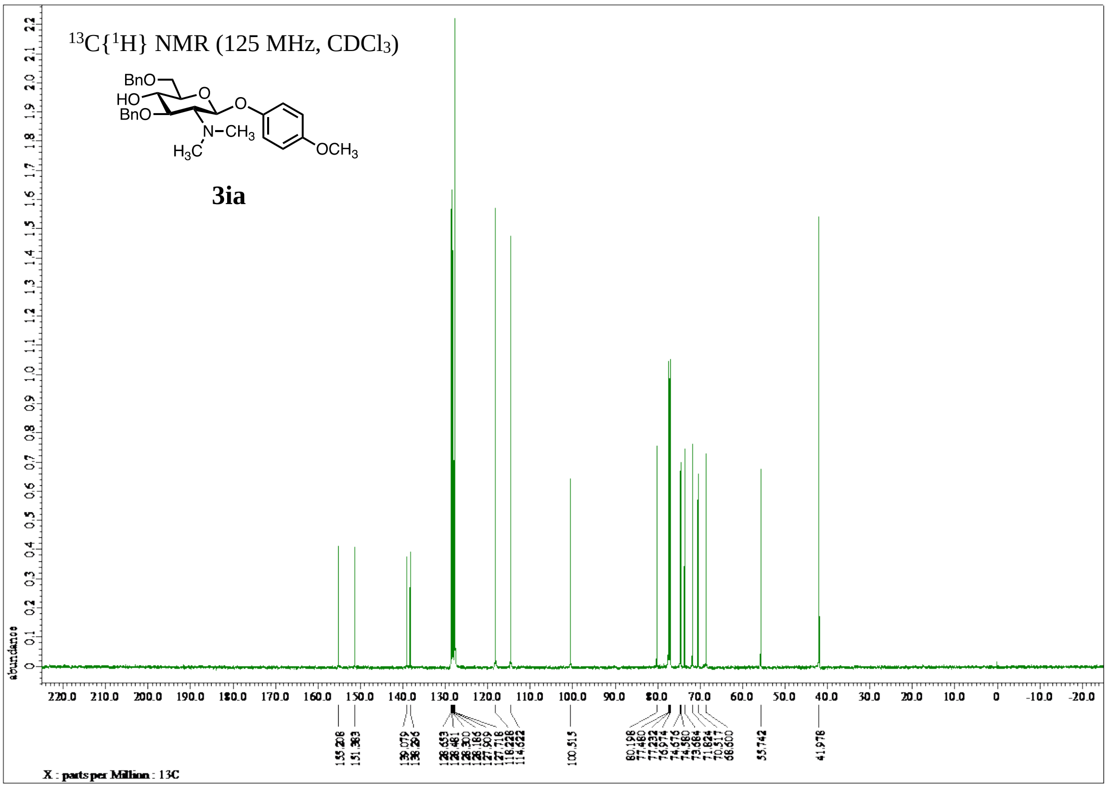

13C{1H} NMR (125 MHz, CDCl3)
3ia
X : parts per Million : 13C

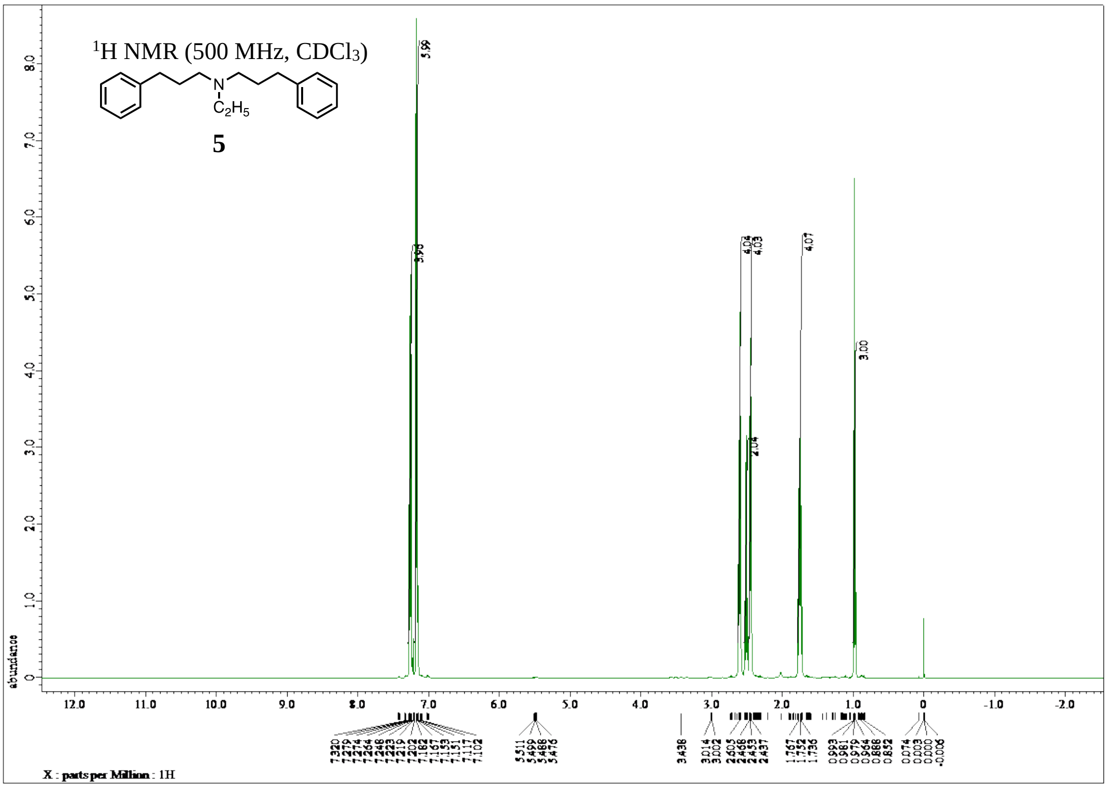

^{1}H NMR (500 MHz, CDCl$_3$)
N
C$_2$H$_5$
5
X : parts per Million : 1H
abundance

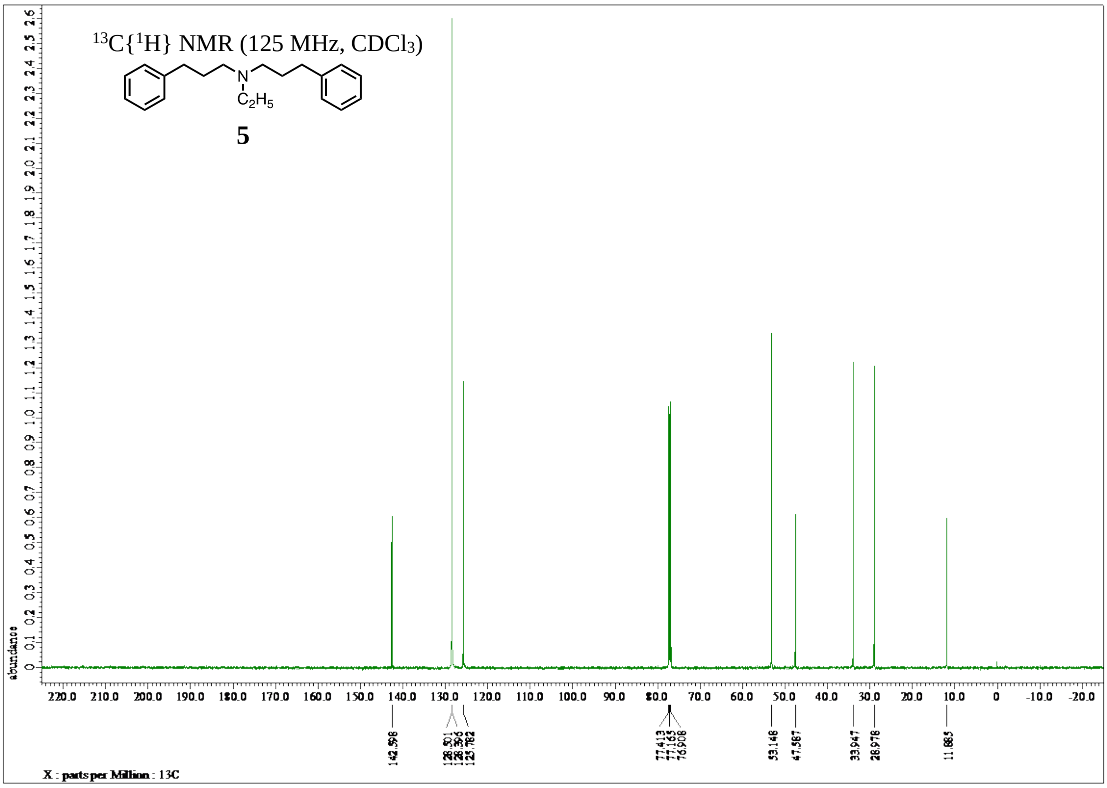

^{13}C{^{1}H} NMR (125 MHz, CDCl$_3$)
N
C$_2$H$_5$
5
X : parts per Million : 13C
abundance

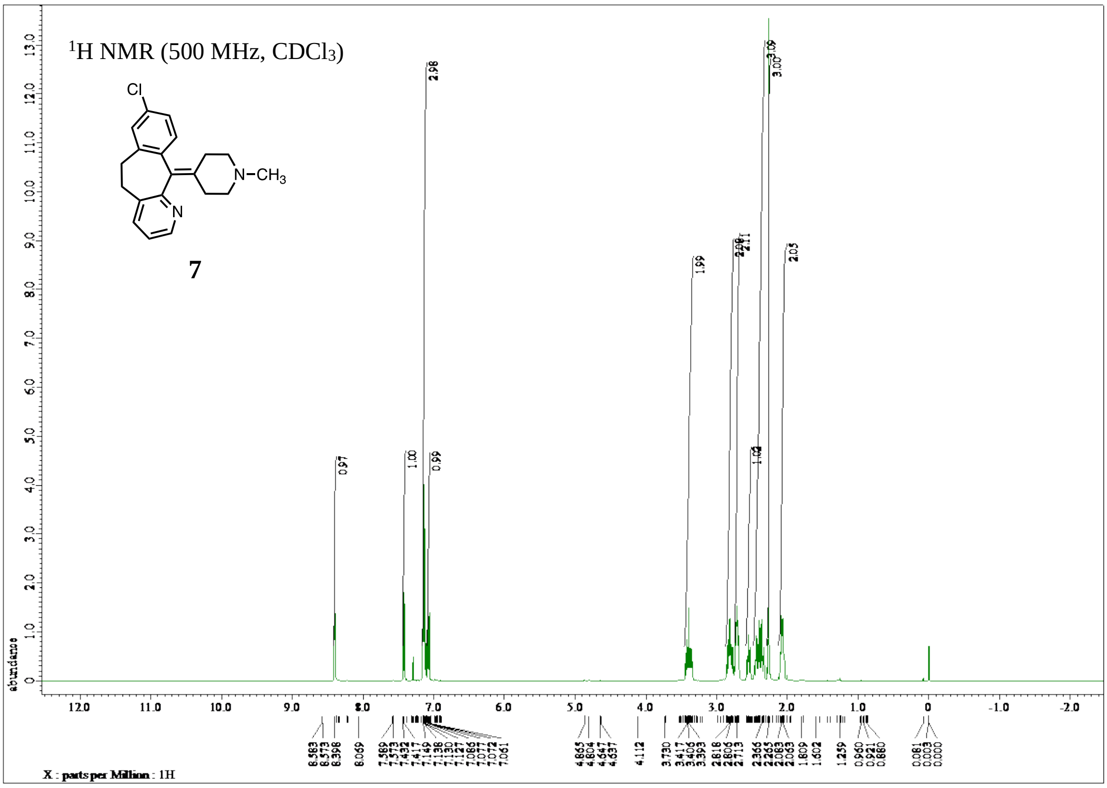

1H NMR (500 MHz, CDCl3)
Cl
N−CH3
N
7
X : parts per Million : 1H
abundance

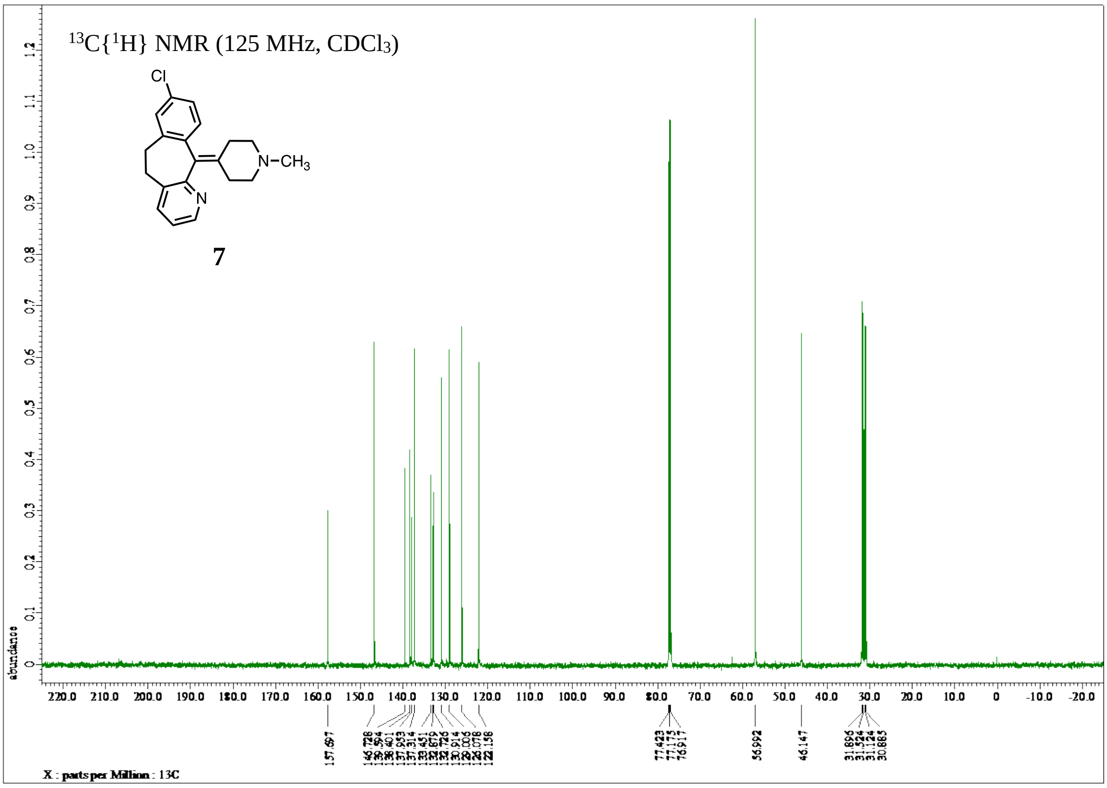

13C{1H} NMR (125 MHz, CDCl3)
Cl
N−CH3
N
7
X : parts per Million : 13C
abundance

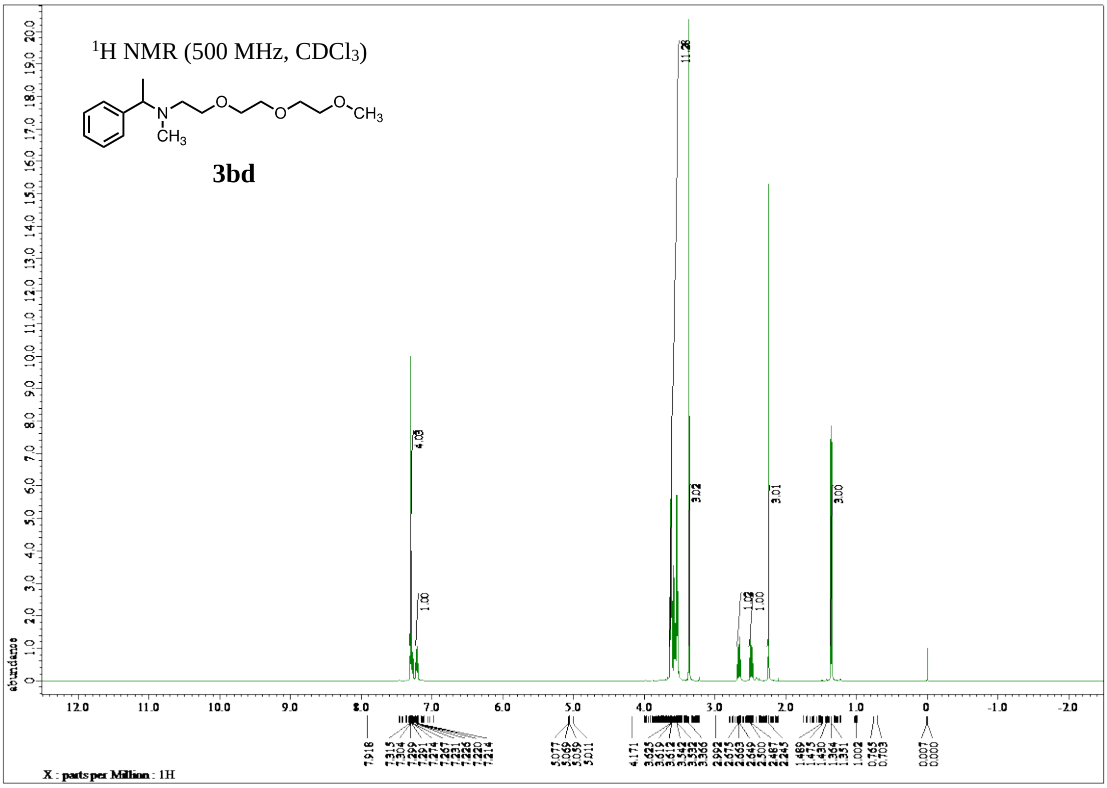
1H NMR (500 MHz, CDCl3)
CH3
N
O
O
O
CH3
3bd
abundance
X : parts per Million : 1H

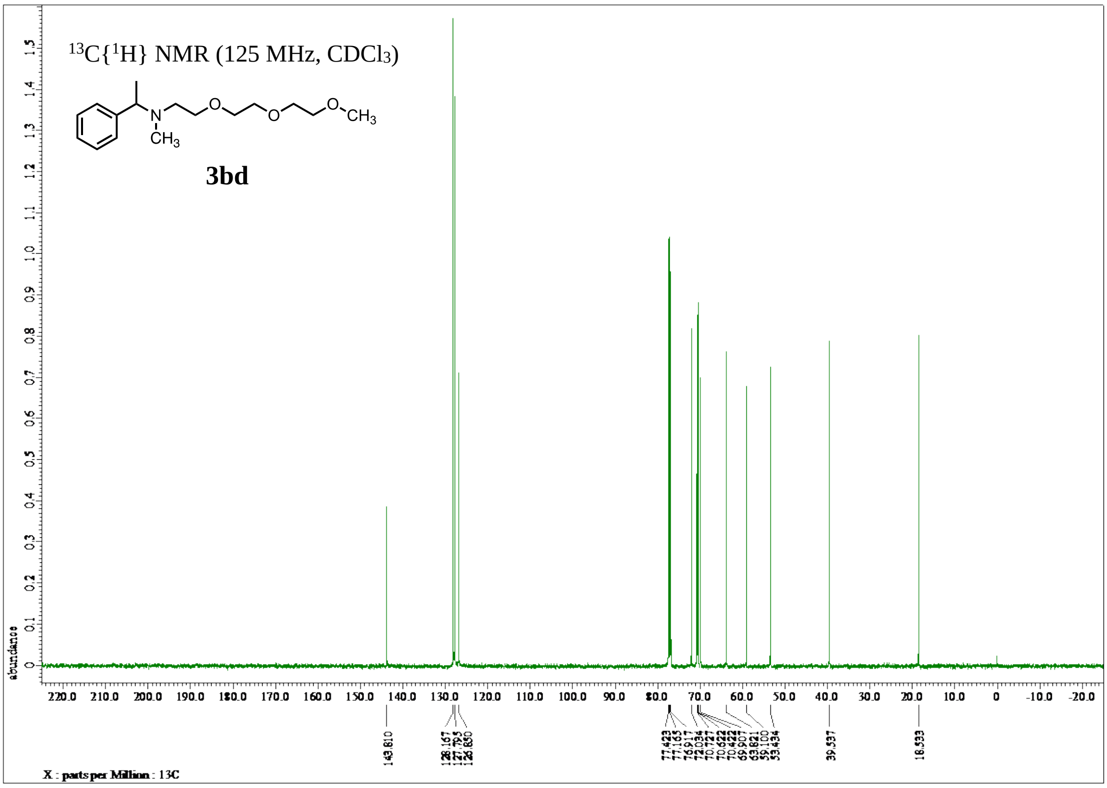
13C{1H} NMR (125 MHz, CDCl3)
CH3
N
O
O
O
CH3
3bd
abundance
X : parts per Million : 13C

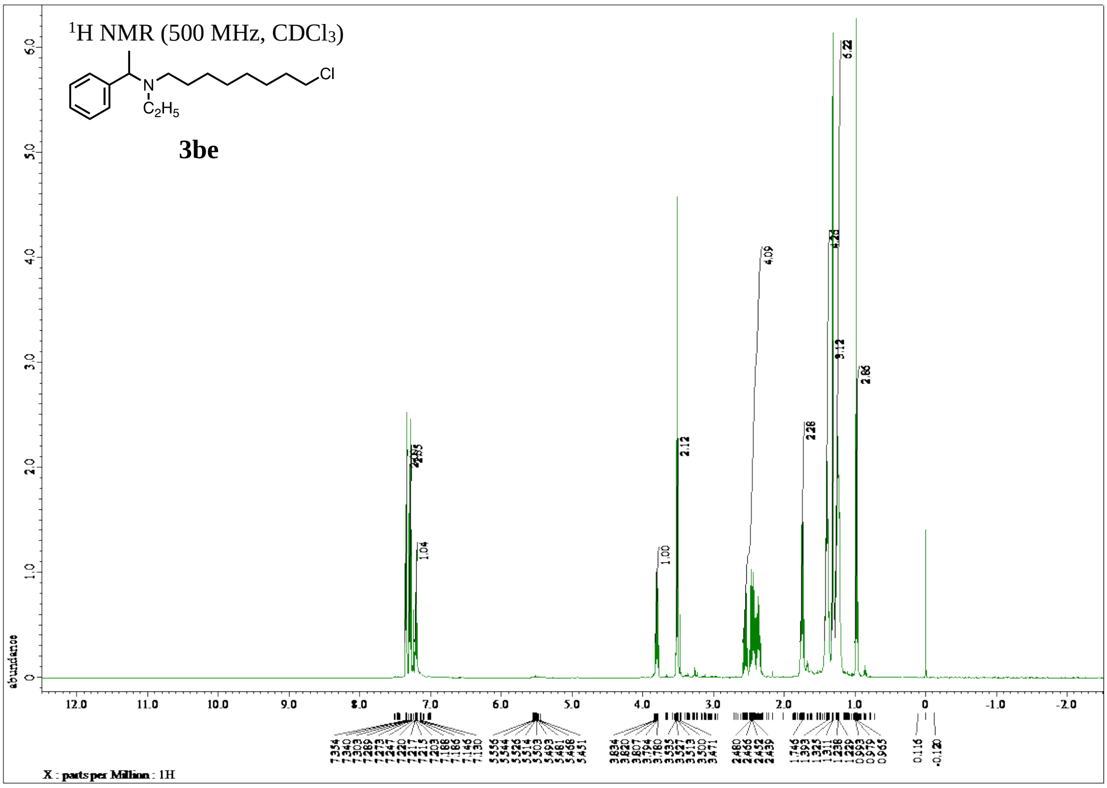
^{1}H NMR (500 MHz, CDCl$_3$)
3be
X : parts per Million : 1H
abundance

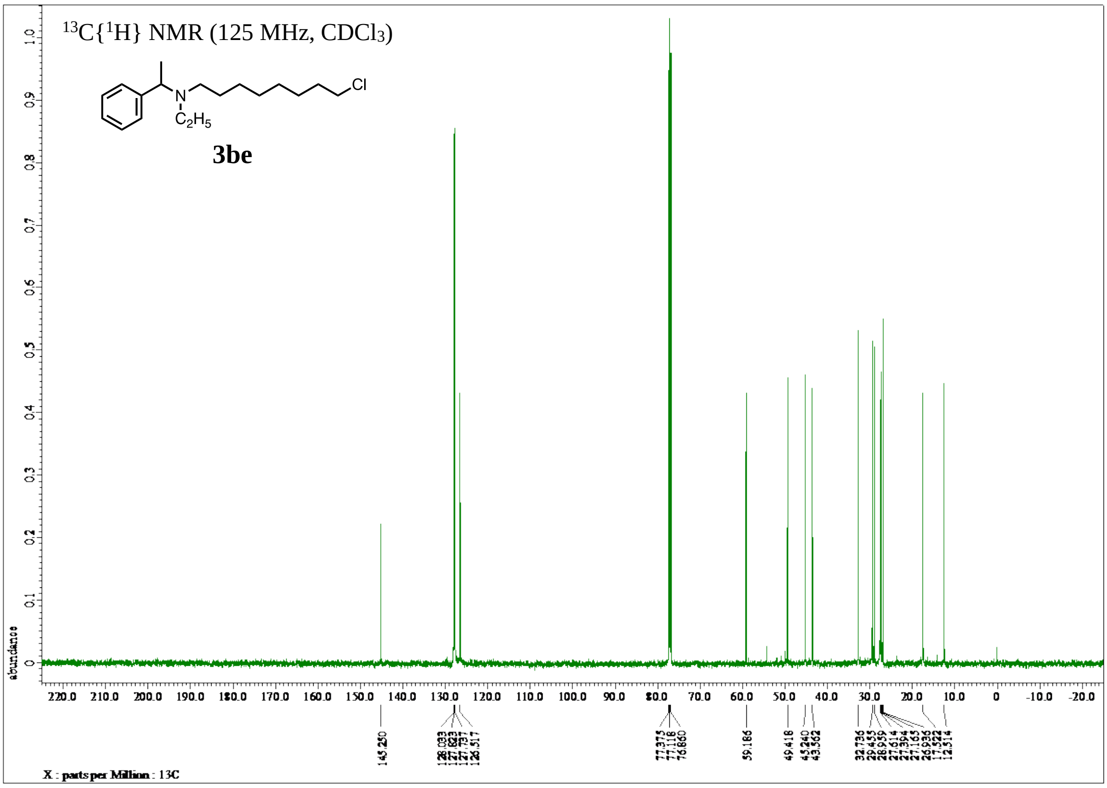
^{13}C{^{1}H} NMR (125 MHz, CDCl$_3$)
3be
X : parts per Million : 13C
abundance

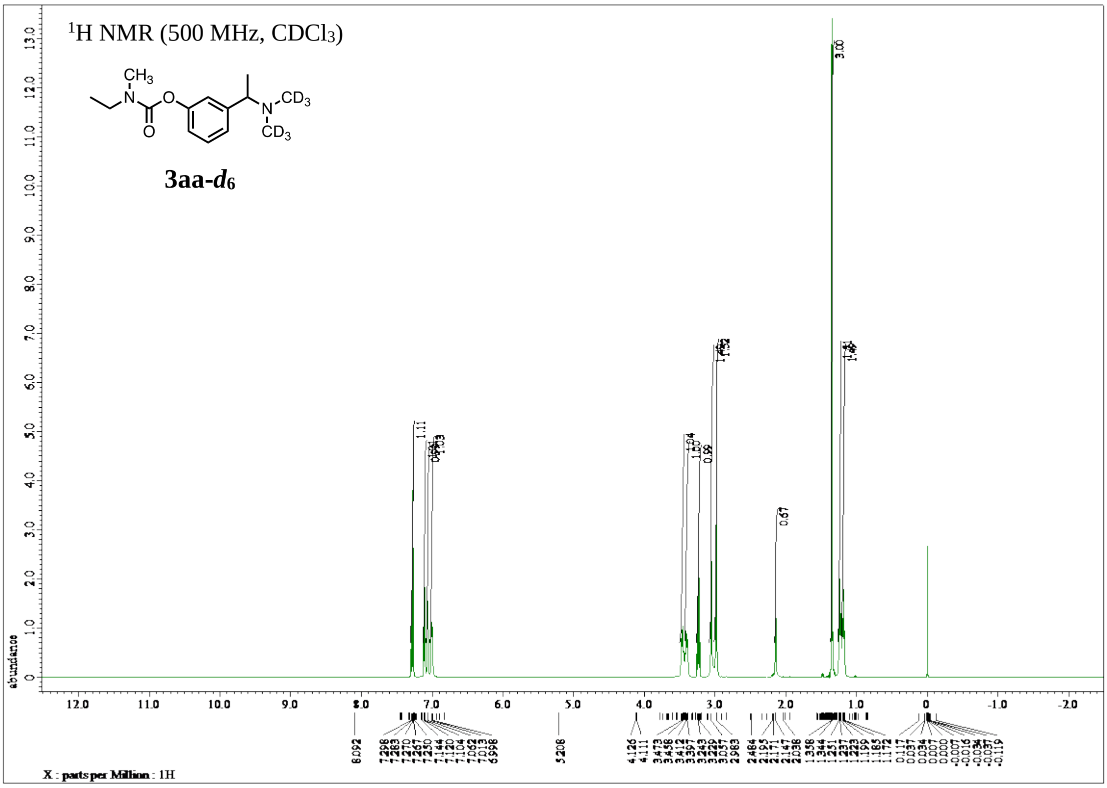

^{1}H NMR (500 MHz, CDCl3)
3aa-d6
X : parts per Million : 1H

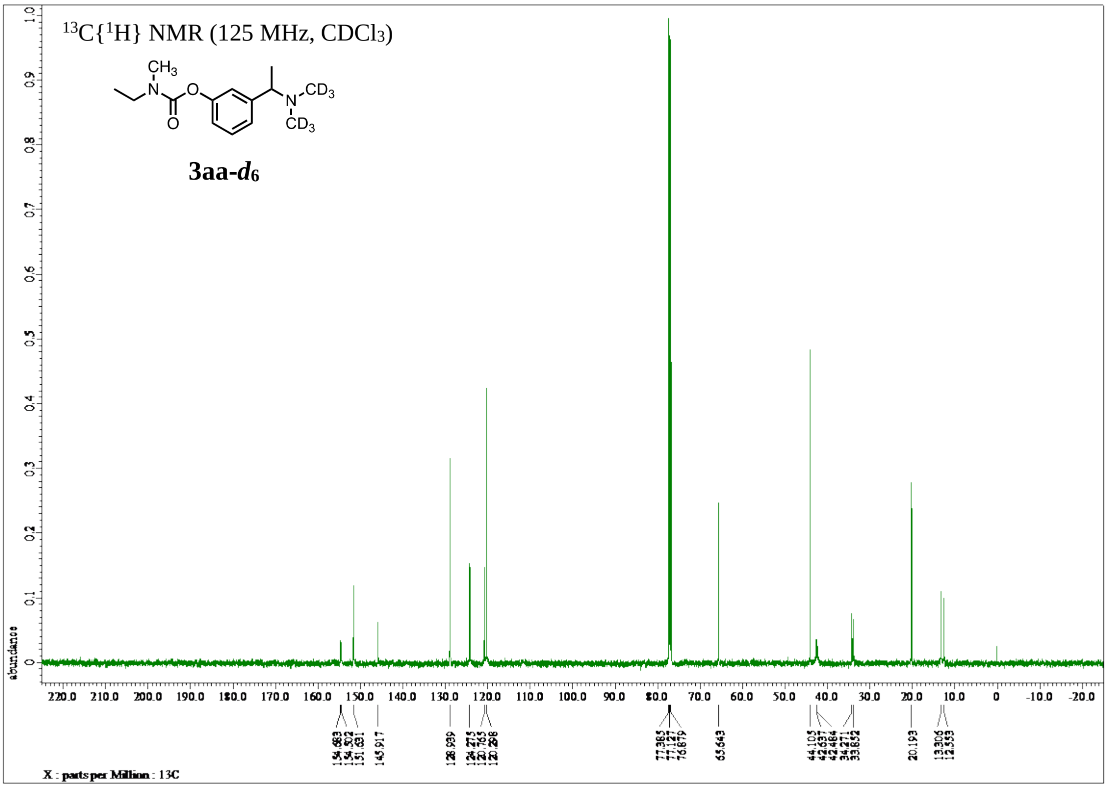

^{13}C{^{1}H} NMR (125 MHz, CDCl3)
3aa-d6
X : parts per Million : 13C

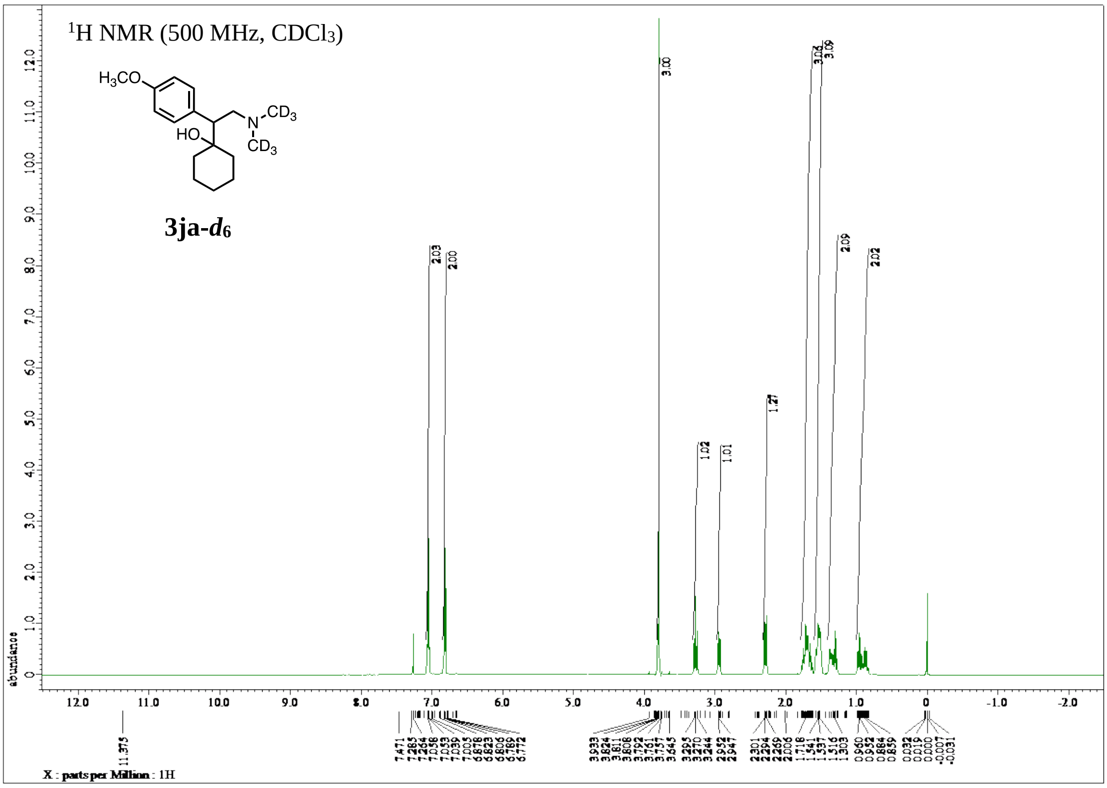
1H NMR (500 MHz, CDCl3)
3ja-d6
X : parts per Million : 1H
abundance

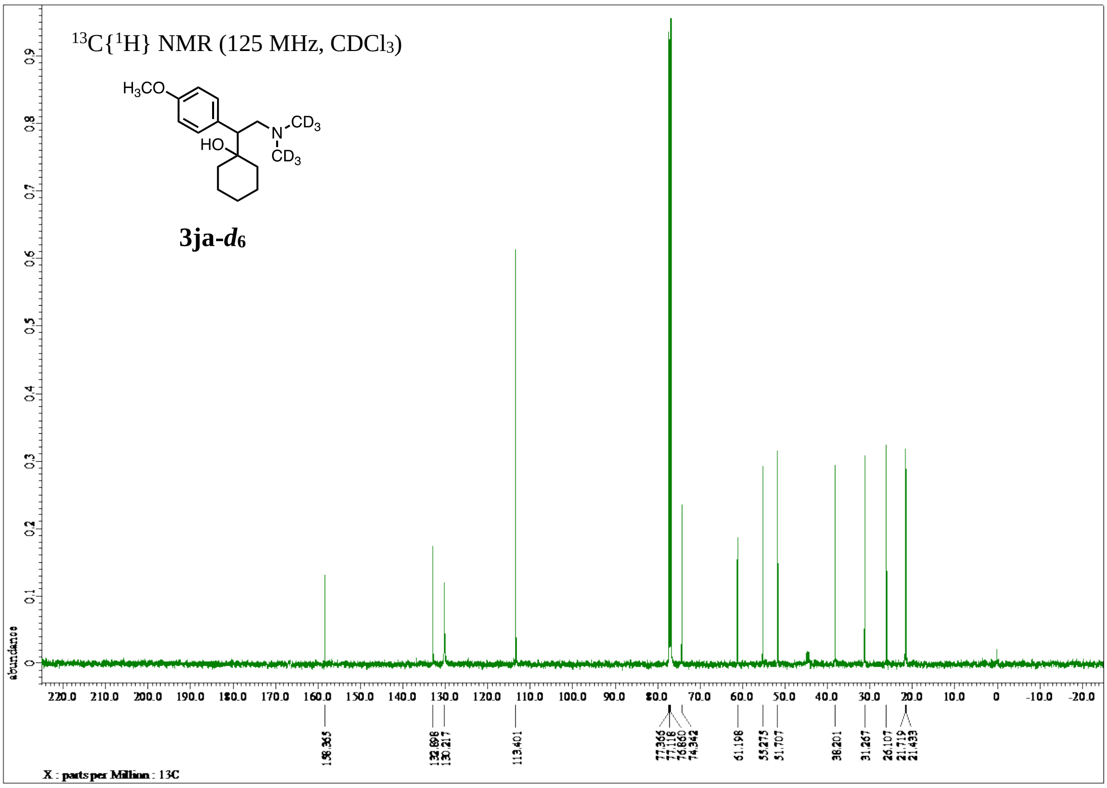
13C{1H} NMR (125 MHz, CDCl3)
3ja-d6
158.355
132.898
130.217
113.401
77.365
77.118
76.860
74.342
61.198
55.275
51.707
38.201
31.267
26.107
21.719
21.433
X : parts per Million : 13C
abundance

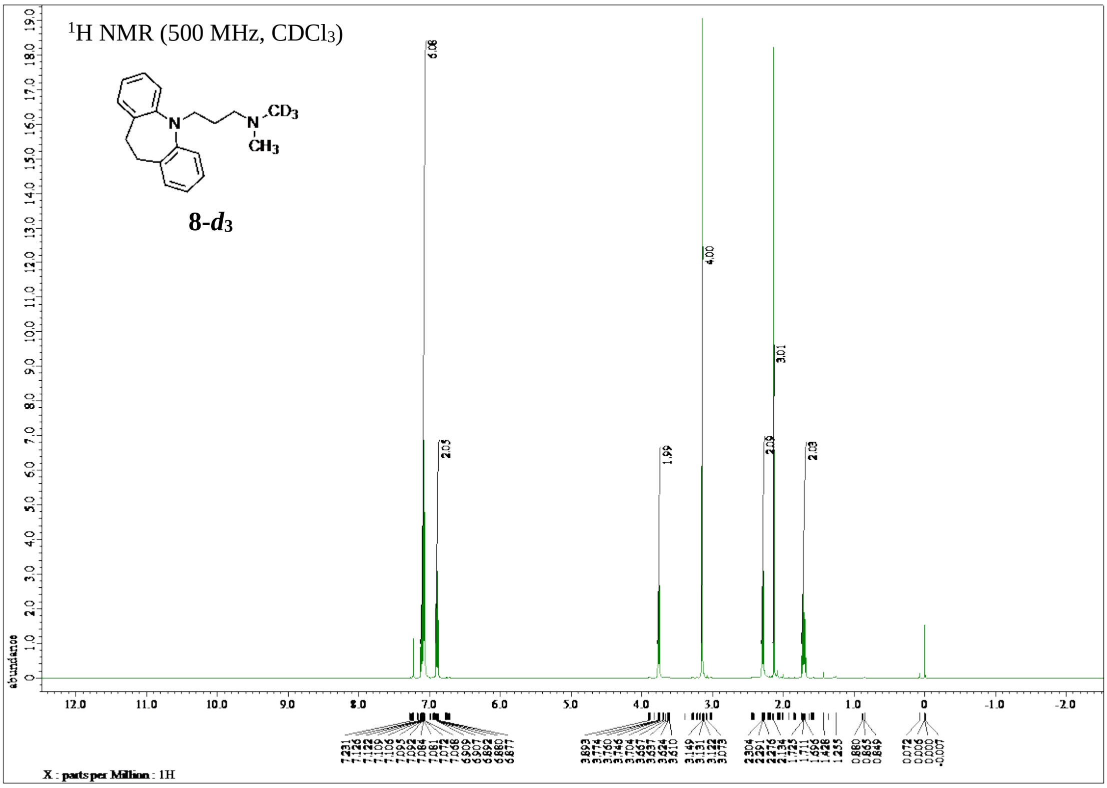

1H NMR (500 MHz, CDCl3)
CD3
N
CH3
8-d3
X : parts per Million : 1H
abundance

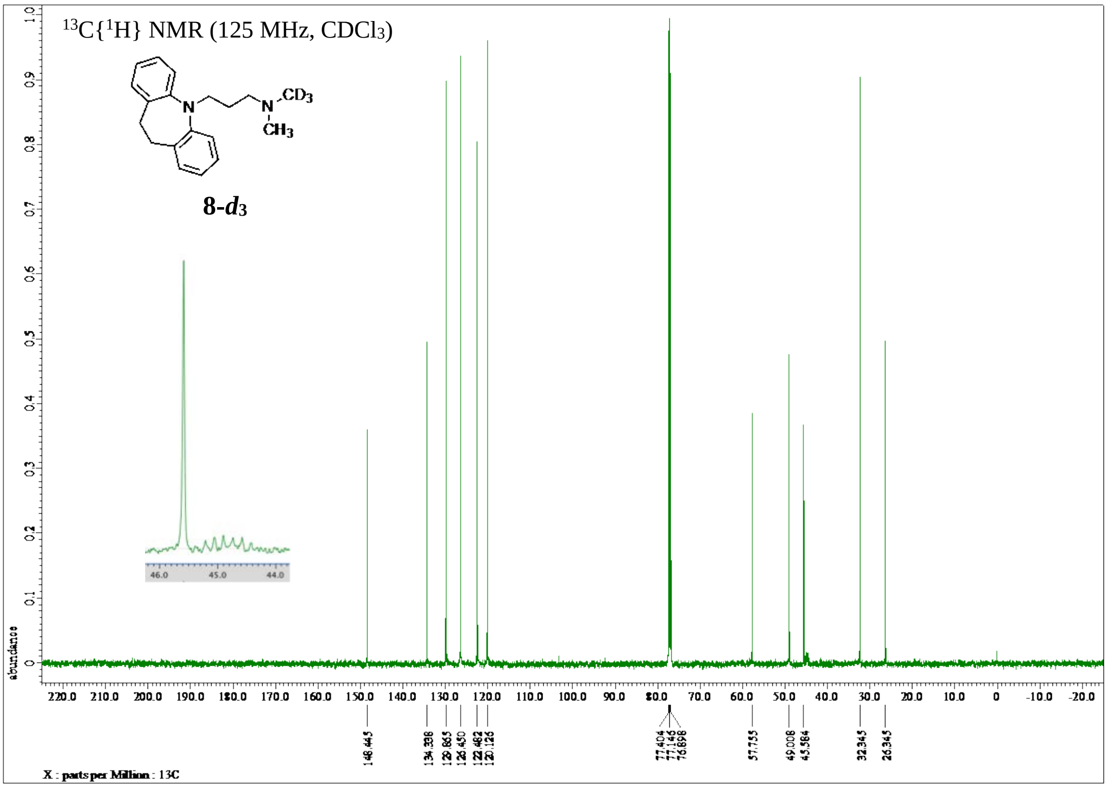

13C{1H} NMR (125 MHz, CDCl3)
CD3
N
CH3
8-d3
148.445
134.338
129.865
126.450
122.482
120.126
77.404
77.146
76.898
57.735
49.008
45.584
32.345
26.345
X : parts per Million : 13C
abundance
